# Genome-Wide Survey of the Soybean GATA Transcription Factor Gene Family and Expression Analysis under Low Nitrogen Stress

**DOI:** 10.1371/journal.pone.0125174

**Published:** 2015-04-17

**Authors:** Chanjuan Zhang, Yuqing Hou, Qingnan Hao, Haifeng Chen, Limiao Chen, Songli Yuan, Zhihui Shan, Xiaojuan Zhang, Zhonglu Yang, Dezhen Qiu, Xinan Zhou, Wenjun Huang

**Affiliations:** 1 Key Laboratory of Oil Crop Biology of the Ministry of Agriculture, Oil Crops Research Institute of the Chinese Academy of Agricultural Sciences, Wuhan, China; 2 Key Laboratory of Plant Germplasm Enhancement and Specialty Agriculture, Wuhan Botanical Garden, Chinese Academy of Sciences, Wuhan, China; National Institute of Plant Genome Research, INDIA

## Abstract

GATA transcription factors are transcriptional regulatory proteins that contain a characteristic type-IV zinc finger DNA-binding domain and recognize the conserved GATA motif in the promoter sequence of target genes. Previous studies demonstrated that plant GATA factors possess critical functions in developmental control and responses to the environment. To date, the GATA factors in soybean (*Glycine max*) have yet to be characterized. Thus, this study identified 64 putative GATA factors from the entire soybean genomic sequence. The chromosomal distributions, gene structures, duplication patterns, phylogenetic tree, tissue expression patterns, and response to low nitrogen stress of the 64 GATA factors in soybean were analyzed to further investigate the functions of these factors. Results indicated that segmental duplication predominantly contributed to the expansion of the GATA factor gene family in soybean. These GATA proteins were phylogenetically clustered into four distinct subfamilies, wherein their gene structure and motif compositions were considerably conserved. A comparative phylogenetic analysis of the GATA factor zinc finger domain sequences in soybean, Arabidopsis (*Arabidopsis thaliana*), and rice (*Oryza sativa*) revealed four major classes. The GATA factors in soybean exhibited expression diversity among different tissues; some of these factors showed tissue-specific expression patterns. Numerous GATA factors displayed upregulation or downregulation in soybean leaf in response to low nitrogen stress, and two GATA factors *GATA44* and *GATA58* were likely to be involved in the regulation of nitrogen metabolism in soybean. Overexpression of *GmGATA44* complemented the reduced chlorophyll phenotype of the *Arabidopsis* ortholog *AtGATA21* mutant, implying that *GmGATA44* played an important role in modulating chlorophyll biosynthesis. Overall, our study provides useful information for the further analysis of the biological functions of GATA factors in soybean and other crops.

## Introduction

GATA transcription factors are a group of regulators that contain the highly conserved type-IV zinc finger motif. These factors bind to the consensus DNA sequence (A/T)GATA(A/G) and are also designated as GATA factors [1]. They were originally identified and characterized in animals and fungi, and typically encoded by multi-gene families. Most proteins include one or two zinc fingers fitting the consensus sequence CX_2_CX_17–18_CX_2_C, followed by a basic region. Animal GATA factors typically contain two CX_2_CX_17_CX_2_C zinc finger domains, and only the C-terminal finger is involved in DNA binding [1–2]. Most fungal GATA factors contain a single CX_2_CX_17_CX_2_C or CX_2_CX_18_CX_2_C domain, which is highly similar to the carboxyl terminal finger of animal GATA factors [3–4]. The first plant GATA factor gene *NTL1* (NIT2-like) was identified from tobacco (*Nicotiana tabacum*) [5]. This finding revealed the presence of GATA factors in higher plants. Previous studies predicted 30 and 29 GATA transcription factors in the *Arabidopsis* and rice genomes, respectively [6–7]. Most plant GATA factors contain a single CX_2_CX_18_CX_2_C domain, but some also contain either zinc finger loops of 20 residues or more than two zinc finger domains [6].

The biological functions of GATA factors have been broadly studied in animals and fungi. Animal GATA factors have critical functions in development, differentiation, and cell proliferation [2]. Fungal GATA factors are involved in the regulation of nitrogen metabolism, light induction, siderophore biosynthesis, and mating-type switching [4]. Substantial evidence indicated that plant GATA factors are involved in different biological functions. In general, plant GATA factors regulate light-mediated and circadian-regulated gene expression [8–14]. Several *Arabidopsis* GATA factors are DNA-binding proteins that interact with light-responsive promoters [15–16]. GATA2 (At2g45050) has been identified as a key transcriptional regulator that mediates the crosstalk between brassinosteroid and light signaling pathways [17]. Some plant GATA factors also serve vital functions in some developmental processes. Several *Arabidopsis* GATA factors have been reported to regulate inflorescence and flower development [18–19], shoot apical meristem development [19], hypocotyl and petiole elongation [20], organ differentiation [21], and seed germination [22]. In addition, GATA factors are involved in the regulation of plant nitrogen metabolism. Previous experiments showed that NIT2, the major nitrogen regulatory protein of *Neurospora crassa* [23], specifically binds to two fragments of the nitrate reductase gene of tomato *in vitro* [24]. The regions of the spinach NiR (nitrite reductase) promoter are involved in nitrogen regulation, and footprinting results suggested that GATA factors function in NiR gene regulation [25]. Recent studies have proven that GNC (GATA factor, Nitrate-inducible, Carbon metabolism-involved) and CGA1/GNL (Cytokinin-responsive GATA1/GNC-Like) serve important functions in chlorophyll synthesis and potentially regulate carbon and nitrogen metabolism [7, 26]. Similarly, *Cga1* (*Cytokinin-responsive GATA transcription factor1*) reportedly regulates chloroplast development in rice. *OsCga1* overexpression maintains chloroplast development under reduced nitrogen conditions, leading to an increased harvest index despite reduced plant size [27]. Several GATA factors have been functionally characterized in *Arabidopsis* and rice. However, the biological functions of most GATA factor family members remain poorly understood.

Soybean (*Glycine max*) is an important food and oil crop that serves as an important protein source for both human consumption and animal feed [28]. To date, few data are available about the GATA factor gene family in soybean. To our knowledge, limited reports exist on the biological functions of soybean GATA factors; one GATA factor (Glyma03g27250) and two GATA factors (Glyma13g00200.1 and Glyma14g10830.1) are involved in soybean nodule development and seed development, respectively [29–30]. The complete soybean genomic sequence has been released and facilitated studies of gene discovery and function [31]. We initially conducted a genome-wide survey of GATA factor-related sequences in soybean to elucidate the functions of GATA proteins in soybean. We identified 64 soybean *GATA* genes. Detailed analyses of phylogenetic relationships, gene structures, chromosomal distribution, duplication patterns, and conserved motifs of all soybean GATA factors were performed. Subsequently, evolutionary relationships among the GATA family in soybean, *Arabidopsis*, and rice, and the expression profiles of all soybean *GATA* genes in various tissues were analyzed. The expression patterns of these *GATA* genes in response to different nitrate conditions were also conducted to investigate the potential functions of soybean GATA factors involved in the regulation of nitrogen metabolism. Our genome-wide systematic analysis of GATA factors in soybean provides a basis for further investigation on the evolution and functions of GATA factors.

## Materials and Methods

### Database searches for the identification of GATA factor family members in soybean

We conducted BLAST and keyword searches to collect all potential soybean proteins containing GATA zinc finger. BLASTP search against the soybean genome was carried out at the National Center for Biological Information (NCBI; http://blast.ncbi.nlm.nih.gov/Blast) using the amino acid sequence of four GATA factors from different origins [*Arabidopsis* AtGATA1 (CAA73999), *Aspergillus nidulans* AreA (P17429), *N*. *crassa* WC1 (Q01371), and chicken GATA1 (AAA49055)] as queries as previously described [6]. All sequences with an *E*-value below 1.0 were collected. A keyword search was conducted at the Phytozome (v9.0) database (http://www.phytozome.net) for putative soybean GATA factors by searching ontologies with the term (PF00320) of GATA domain. If more than one transcript existed, the primary transcript was selected as representative. These collected putative GATA factor genes were confirmed using the Pfam (http://pfam.sanger.ac.uk/) and InterPro (https://www.ebi.ac.uk/interpro/) databases. Soybean expressed sequence tag (EST) sequences were searched by blastn program in the Gene Indices at DFCI (http://compbio.dfci.harvard.edu/tgi/) using the transcript sequences of the identified putative soybean GATA factors as queries.

### Phylogenetic tree constructions

Phylogenetic analysis was performed using MEGA5 software [32]. ClustalW was used to conduct multiple alignments of the full-length deduced amino acid sequences of soybean GATA factors or the conserved GATA zinc finger domain sequences of the GATA factors in soybean, *Arabidopsis*, and rice. Then, a phylogenetic tree was constructed by the neighbor-joining method with the Poisson substitution model, uniform rates, and pairwise deletion. A total of 1000 bootstrap replicates were carried out to identify the phylogeny.

### Gene structure and chromosomal location

For exon/intron structural analysis, the genomic DNA and cDNA sequences corresponding to each predicted soybean GATA factor gene were downloaded from the Glyma (v1.1) of Phytozome or NCBI database. Their exon/intron structures were analyzed using the gene structure display server program (http://gsds.cbi.pku.edu.cn) [33]. The chromosomal location of soybean *GATA* genes was generated using Chromosome Visualization Tool (CViT) at the Legume Information System (http://comparative-legumes.org/) [34]. The presence of soybean GATA factor genes in segmental duplication blocks was investigated using CViT and synteny viewer as previously described [35].

### Identification of conserved motifs in soybean GATA proteins

The conserved motifs of 64 soybean GATA protein sequences were analyzed by the Multiple Em for Motif Elicitation (MEME) program (http://meme.nbcr.net/meme/cgi-bin/meme.cgi) [36]. We set the distribution of a single motif among the sequences as “any number of repetitions”, the maximum number of motifs as 30, and the width of each motif as 6 to 100. The functional annotation of the identified motifs was performed using the Pfam and InterPro databases.

### Plant materials and treatments

Soybean (*G*. *max* L.) low nitrogen-tolerant variety “No. 116” [37] was used as the plant material. Soybean seeds were germinated and grown in a greenhouse. Roots, stems, young leaves, mature flowers, and immature seeds were collected from adult plants for gene expression analysis. Low nitrogen stress treatment was performed at 10 d after germination as follows. Soybean seedlings with cut-off cotyledons were transferred to half Hoagland solution for 4 d and then transferred to low nitrogen (10% of the normal nitrogen concentration) half Hoagland solution when the primary leaves unfolded. The half Hoagland hydroponic solution (pH 6.0) contained 2 mM Ca(NO_3_)_2_·4H_2_O, 2.5 mM KNO_3_, 0.5 mM NH_4_NO_3_, 0.5 mM KH_2_PO_4_, 1 mM MgSO_4_·7H_2_O, 0.05 mM Fe-EDTA, 0.005 mM KI, 0.1 mM H_3_BO_3_, 0.1 mM MnSO_4_·H_2_O, 0.03 mM ZnSO_4_·7H_2_O, 0.0001 mM CuSO_4_·5H_2_O, 0.001 mM Na_2_MO_4_·2H_2_O, and 0.0001 mM CoCl_2_·6H_2_O. To compensate the concentration of Ca^2+^ and K^+^, the low nitrogen solution was prepared by replacing Ca(NO_3_)_2_·4H_2_O and KNO_3_ with CaSO_4_ and K_2_SO_4_, respectively. The culture solution was changed every 3 d. After 4 h, 3 d, and 6 d of low nitrogen stress treatment, the leaves and roots were harvested separately, with three biological replicates per sample. Untreated seedlings in half Hoagland solution were used as controls for all samples. The collected plant materials were immediately frozen in liquid nitrogen and stored at −80°C for RNA isolation.

The *Arabidopsis thaliana* seeds of Columbia ecotype and a mutant were surface-sterilized with 10% (w/v) NaClO and thoroughly washed three times with sterile water. After stratification at 4°C for 3 days in darkness, seeds were sown on Murashige and Skoog (MS) medium containing 3% sucrose and 0.8% agar in the illuminated incubator. Seedlings were transplanted to soil 10 days after germination in the growth chamber. The illuminated incubator and growth chamber were both controlled at 23°C with 16/8 h (light/dark) photoperiod. The mutant of *AtGATA21* (*gnc*, SALK_001778) was obtained from the Arabidopsis Biological Resource Center (ABRC).

### Vector construction and *Arabidopsis* transformation

To generate the 35S::GmGATA44 overexpression construct, the coding sequence of *GmGATA44* was amplified using the primers 5′-ATGATTCCAGCCTATCGCC-3′ and 5′-TCAATGAACAAGGCCATAAGATA-3′. Then it was cloned into the pGWC vector and recombined into the pB2GW7 using the LR recombinase reaction (Invitrogen, USA). The recombinant construct containing the 35S::GmGATA44 cassette was introduced into *Agrobacterium tumefaciens* strain GV3101 by freeze-thaw method and then transformed into the *Arabidopsis* homozygous mutant *gnc* via floral dip method [38]. The *gnc* mutant has a T-DNA insertion in the gene locus *At5g56860*, encoding a GATA protein AtGATA21. The transgenic plants were screened on MS medium containing 3% (w/v) sucrose and 20 mg/L Basta and confirmed by PCR analyses. The transcript levels of *GmGATA44* and *AtGNC* were determined by semi-quantitative reverse transcriptase (RT)-PCR, and *UBQ10* (*At4g05320*) was used as a reference control. In addition, chlorophyll contents in transgenic *Arabidopsis* leaves were measured as previously described [39].

### RNA extraction, semi-quantitative RT-PCR and quantitative real-time PCR

Total RNA was extracted from the roots, stems, leaves, flowers, and seeds of soybean plants using Trizol reagent (Invitrogen, USA) according to the manufacturer’s instruction. The quality of the RNA was assessed by agarose gel electrophoresis, and the concentration was measured by an Epoch microplate spectrophotometer (BioTek, USA). RNA samples were treated with RNase-free DNase I (Thermo Scientific, USA) to avoid DNA contamination. First-strand cDNA was synthesized from 2 μg RNA using M-MLV reverse transcriptase (Promega, USA) according to the supplier’s protocol. Semi-quantitative RT-PCR for gene expression in *Arabidopsis* plants was carried out using the following program: an initial denaturation of 94°C for 5 min, followed by 31 cycles of 94°C for 30 s, 56°C for 30s, and 72°C for 30s, and a final extension at 72°C for 10 min. PCR products were detected by 1% agarose gel. Quantitative real-time PCR for gene expression in soybean and *Arabidopsis* plants was performed on the Rotor-Gene Q (Qiagen, Germany) using SYBR Green SuperReal Premix (Tiangen, China). Real-time PCR primers were designed using Primer 5.0 software. Primer specificity was verified using the BLAST tool from the NCBI database. The housekeeping genes *ACT11* (*Glyma18g52780*) and *GAPDH* (*At3g26650*) were used as the endogenous control to normalize the samples of soybean and *Arabidopsis*, respectively. The thermal cycling conditions were as follows: 95°C for 15 min; 40 cycles of 95°C for 10 s, 60°C for 15s, and 72°C for 20s. All reactions were performed at least in triplicate. Relative gene expression was analyzed using the 2^−ddCt^ method. All primers for semi-quantitative RT-PCR and quantitative real-time PCR were listed in S1–S3 Tables.

## Results and Discussion

### GATA factor family in soybean

BLASTP searches in the soybean database of NCBI using *Arabidopsis* full-length GATA1 protein sequences, as well as sequences from *A*. *nidulans* AreA, *N*. *crassa* WC1, and chicken GATA1, yielded 56 sequences. Keyword search in the phytozome soybean genome database using the GATA domain (PF00320) yielded 63 candidate sequences. Finally, 64 different soybean loci encoding GATA proteins were identified by removing redundant sequences and different transcripts of the same gene. All these putative GATA protein sequences contained the conserved GATA zinc finger domain, which was confirmed by Pfam and InterPro. Soybean had relatively more GATA factors than *Arabidopsis* and rice, with 30 and 29, respectively [6–7]. The members of the GATA factor family in soybean were 2.1- and 2.2-times those in *Arabidopsis* and rice, respectively.

The 64 soybean GATA factors were named *GmGATA1* to *GmGATA64* according to their chromosomal positions. Table 1 provides detailed information on soybean *GATA* genes. The nucleotide and amino acid sequences of these soybean GATA factors are available in S1 Text. The identified soybean GATA factors encoded peptides ranging from 80 to 551 amino acids with the isoelectric point (pI) varying from 4.63 to 9.66 and the molecular weight (Mw) varying from 9.1 kD to 60.8 kD. All *GmGATA* genes contained the full-length coding sequence (CDS), except for *GmGATA48*. Analysis of the soybean EST databases indicated that partial cDNA sequences were reported for 53 of the 64 *GmGATA* factor genes (Table 1).

**Table 1:** GATA gene family in soybean.

Gene name	Locus name	GenBank Accession No	Genome Location	Peptide Length	Subfamily	Number of EST	pI	Mw (KD)
GmGATA1	Glyma01g10390	XM_006573963	1 (13444507–13445181)	154	I	10	8.16	17.9
GmGATA2	Glyma01g37450	NM_001255324	1 (49804698–49806831)	352	I	16	5.58	39.3
GmGATA3	Glyma01g41370	XM_003517352	1 (52875919–52881926)	551	IV	6	6.38	60.7
GmGATA4	Glyma02g05710	XM_003519830	2 (4571075–4572829)	351	I	5	6.97	38.8
GmGATA5	Glyma02g06320	XM_003519850	2 (5032247–5034308)	252	II	0	7.66	28.2
GmGATA6	Glyma02g07850	XM_003518166	2 (6203860–6206352)	280	I	11	8.3	31.3
GmGATA7	Glyma02g08145	XM_006574699	2 (6375823–6381916)	333	I	0	5.67	36.1
GmGATA8	Glyma02g37980	XM_003518220	2 (43271674–43276335)	310	III	10	6.08	33.3
GmGATA9	Glyma03g27250	XM_003521041	3 (34875366–34876853)	226	I	4	7.23	25.5
GmGATA10	Glyma03g39220	XM_006577154	3 (45384711–45385677)	80	II	2	9.54	9.1
GmGATA11	Glyma04g01090	XM_003522812	4 (648001–649711)	305	I	13	6.61	33.9
GmGATA12	Glyma04g05431	XM_006578015	4 (4112757–4114427)	292	II	0	9.15	32.5
GmGATA13	Glyma04g08990	XM_003523698	4 (7123655–7125290)	305	I	3	5.82	33.9
GmGATA14	Glyma04g10330	XM_003523746	4 (8555417–8560793)	309	III	0	5.75	33.8
GmGATA15	Glyma04g10340	XM_003522723	4 (8563510–8577349)	350	III	8	4.63	37.9
GmGATA16	Glyma05g05320	XM_003524080	5 (4651466–4657300)	542	IV	2	6.31	60.2
GmGATA17	Glyma05g30385	XM_006580167	5 (35775012–35778754)	164	IV	0	8.21	18.7
GmGATA18	Glyma05g30390		5 (35782101–35784464)	151	IV	0	9.63	17.4
GmGATA19	Glyma06g01110	XM_003527087	6 (662860–664553)	294	I	23	6.67	32.9
GmGATA20	Glyma06g09080	XM_003527795	6 (6665494–6667093)	326	I	0	5.93	36.0
GmGATA21	Glyma06g10280	XM_006580925	6 (7764041–7769818)	304	III	13	5.7	33.4
GmGATA22	Glyma06g10290	NM_001255457	6 (7772054–7779091)	351	III	12	4.67	38.1
GmGATA23	Glyma07g01960	XM_003530066	7 (1354648–1356366)	409	I	1	7.06	45.2
GmGATA24	Glyma07g14750	XM_003530126	7 (14558214–14560706)	237	I	5	6.75	26.5
GmGATA25	Glyma07g30140	XM_006583711	7 (35186721–35192803)	355	III	0	4.91	39.6
GmGATA26	Glyma07g37190	NM_001248912	7 (42333282–42334247)	130	II	28	9.59	13.7
GmGATA27	Glyma08g07170	XM_003532550	8 (5156192–5161740)	358	III	1	5.24	39.9
GmGATA28	Glyma08g15061	XM_006585246	8 (10938066–10939051)	136	II	1	6.42	15.5
GmGATA29	Glyma08g19681	XM_006585408	8 (14863972–14869093)	192	IV	0	9.24	21.5
GmGATA30	Glyma08g21630	XM_003531606	8 (16457753–164593338)	347	I	2	6.7	38.2
GmGATA31	Glyma08g23720	XM_003531711	8 (18071626–18076907)	300	III	10	6.19	33.0
GmGATA32	Glyma08g45835	XM_003530797	8 (45105002–45109921)	282	IV	1	5.94	31.2
GmGATA33	Glyma09g07090	XM_003534865	9 (5932308–5935017)	337	II	6	9.73	38.2
GmGATA34	Glyma10g25480	NM_001255531	10 (33374598–33378291)	245	I	1	8.1	27.0
GmGATA35	Glyma10g35470	XM_003536302	10 (43685975–43689694)	347	I	2	5.65	37.3
GmGATA36	Glyma11g04060	XM_003539037	11 (2712320–2718194)	551	IV	1	6.23	60.8
GmGATA37	Glyma11g07350	XM_006590627	11 (5150063–5151830)	245	II	11	8.3	27.3
GmGATA38	Glyma11g11930	XM_003538957	11 (8506932–8508989)	299	I	1	6.03	33.5
GmGATA39	Glyma11g20480	XM_003538144	11 (17278618–17283515)	305	I	26	9.25	34.5
GmGATA40	Glyma11g25375	XM_003539243	11 (24001667–24002360)	156	II	0	9.01	17.6
GmGATA41	Glyma12g04180	XM_003540620	12 (2731228–2733212)	289	I	1	5.32	31.9
GmGATA42	Glyma12g08131	XM_003539719	12 (5856841–5863863)	304	I	2	8.56	34.6
GmGATA43	Glyma12g29730	XM_003540138	12 (33187595–33192408)	326	I	1	8.7	36.1
GmGATA44	Glyma13g00200	XM_003543677	13 (8613–10819)	314	II	3	9.26	35.3
GmGATA45	Glyma13g40020	XM_003543431	13 (40579059–40583715)	327	I	0	8.74	36.1
GmGATA46	Glyma14g10830	XM_003545327	14 (9019823–9023050)	306	II	3	9.54	34.3
GmGATA47	Glyma14g22460	XM_003545560	14 (26583812–26586274)	383	I	3	6.18	42.5
GmGATA48	Glyma14g24201		14 (28955340–28956098)	183*	I	0		
GmGATA49	Glyma14g36150	XM_003544786	14 (45351002–45356038)	307	III	22	6.23	33.3
GmGATA50	Glyma15g05065	XM_006598234	15 (3636636–3637215)	193	II	0	6.43	21.4
GmGATA51	Glyma15g18380	XM_003546407	15 (15112589–15114772)	315	II	4	9.23	35.4
GmGATA52	Glyma16g04670	XM_003548710	16 (3926537–3928215)	281	I	0	6.25	31.5
GmGATA53	Glyma16g24381	XM_006599914	16 (28259082–28263005)	225	I	0	9.62	24.8
GmGATA54	Glyma16g25370	XM_003548840	16 (29320916–29322986)	251	II	1	6.66	28.1
GmGATA55	Glyma16g26870	XM_003548012	16 (30961775–30963809)	279	I	23	8.29	31.1
GmGATA56	Glyma16g27171	XM_003548024	16 (31186968–31192884)	333	I	5	5.62	35.9
GmGATA57	Glyma17g03410	XM_003550478	17 (2280211–2281440)	140	II	5	9.62	14.4
GmGATA58	Glyma17g06290	XM_003550586	17 (4473612–4475846)	322	II	6	9.49	36.2
GmGATA59	Glyma17g15610	XM_003549894	17 (12356334–12362270)	544	IV	0	6.59	60.3
GmGATA60	Glyma17g27110	XM_003550072	17 (28534119–28536348)	366	I	5	6.13	40.8
GmGATA61	Glyma17g34670	XM_006601169	17 (38656842–38660298)	306	II	2	9.39	34.0
GmGATA62	Glyma19g28650	XM_003553957	19 (36220313–36221912)	274	I	18	6.01	30.6
GmGATA63	Glyma19g41780	XM_003554576	19 (47959000–47959480)	96	II	0	9.66	11.0
GmGATA64	Glyma20g32050	XM_003556186	20 (40684758–40688188)	348	I	9	5.39	37.6

Asterisk indicates that the sequence is partial.

All soybean GATA factors contain a single zinc finger. To further investigate the features of the GATA zinc finger domain, the conserved GATA zinc finger domains consisting of approximately 55 residues from 64 soybean GATA factors were aligned (S1 Fig). Except the two pairs of Cys residues, Thr-15, Pro-16, Arg-19, Gly-21, Pro-22, and the amino acid around the second pair of Cys residues (LCNACG) were conserved in almost all the sequences. These highly conserved residues are similar to the GATA factors of *Arabidopsis* and rice [6]. Most *GmGATA* genes encode GATA factors with 18 residues in the zinc finger loop (CX_2_CX_18_CX_2_C), and nine *GmGATA* genes encode GATA factors with 20 residues in the zinc finger loop (CX_2_CX_20_CX_2_C). Similar to *Arabidopsis* and rice, soybean does not contain the animal- and fungal-type CX_2_CX_17_CX_2_C zinc finger domains.

Notably, three *GmGATA* genes have an atypical GATA zinc finger. GmGATA50 presented four rather than two residues between the first and the second Cys residues of the zinc finger (CTNFYC). A similar irregularity has been found in the *Caenorhabditis elegans* GATA factor END-1 and *Arabidopsis* GATA factor AtGATA29, which may function in recognizing GATA DNA motifs [40]. Meanwhile, the GATA factors GmGATA28 and GmGATA48 only have half GATA motif (CANCDTTSTPLWRNAP for GmGATA28 and TPQWRVKPLGPKTLCKAC for GmGATA48). These sequences may be the remains of an ancestral entire zinc finger. The half GATA motif has also been found in the rice GATA factor OsGATA24 [6].

### Phylogenetic relationships and gene structures of the GATA factor family genes in soybean

**Fig 1:**
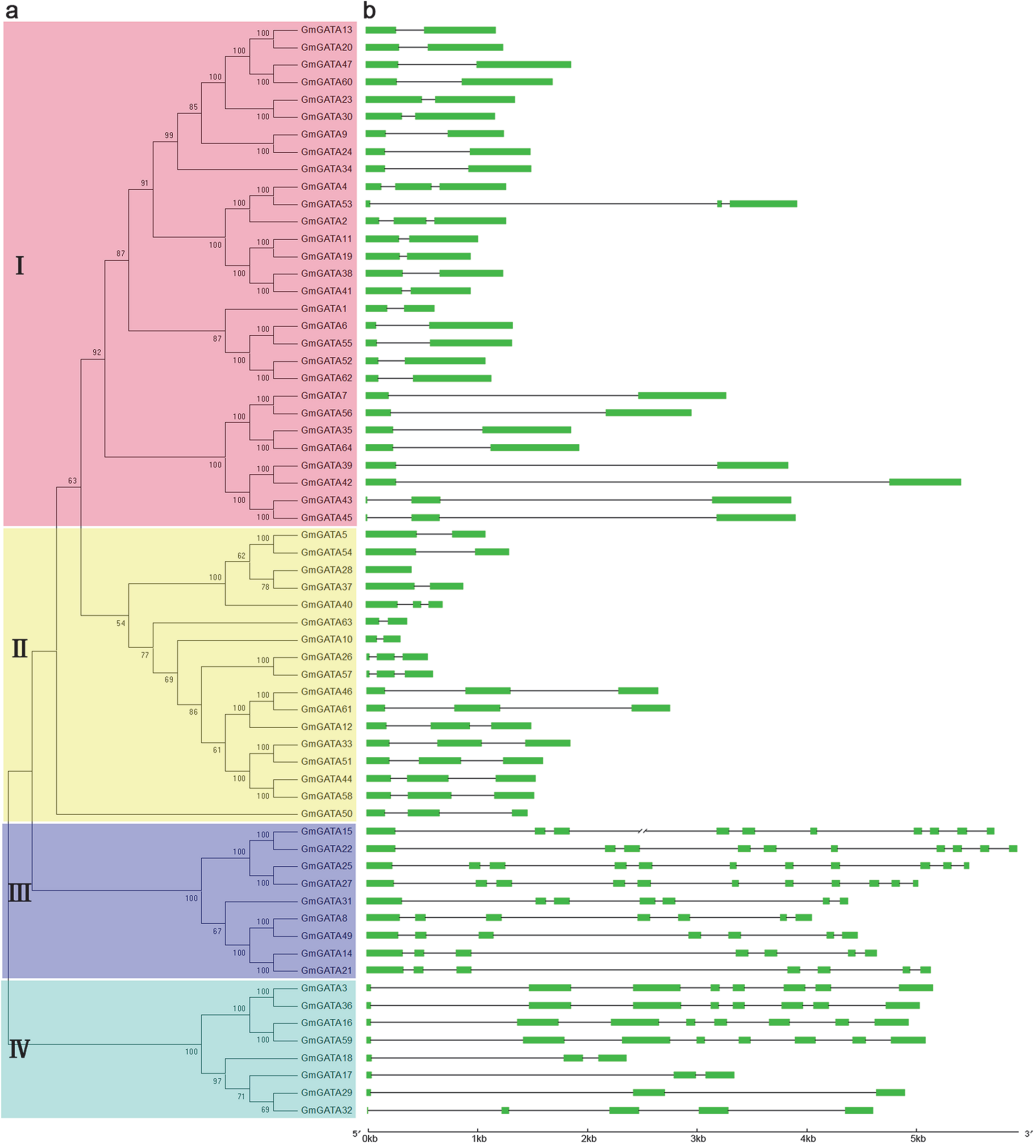
Phylogenetic analysis and gene structure of soybean GATA factors. (a) Phylogenetic tree construction of soybean GATA factors based on the full-length deduced amino acid sequences using MEGA 5.0 by the neighbor-joining method with 1000 bootstrap replicates. Bootstrap values are shown as percentages (>50%) on the branches. GmGATA48 was not presented in this tree because its sequence is partial. The tree showed four major phylogenetic subfamilies (subfamilies I to IV) indicated with different colored backgrounds.(b) Exon/intron structures of *GmGATA* genes. Green boxes represent exons, and black lines indicate introns. *GmGATA48* was not displayed in this figure because its sequence is partial. The 7 kb length base pair was represented with slash–slash. The sizes of exons and introns can be estimated using the scale at the bottom.

To determine the phylogenetic relationships among the different members of the GATA factor family in soybean, a phylogenetic analysis based on alignments of the 63 full-length GATA protein sequences was performed, except GmGATA48. As shown in Fig 1A, the neighbor-joining phylogenetic tree divided 63 *GmGATA* genes into four clades. Previous reports classified seven subfamilies (I, II, III, IV, V, VI, and VII) of GATA factors from *Arabidopsis* and rice GATA factor gene families [6]. Subfamilies I, II, III, and IV were present in soybean. The gene structures of the corresponding genes are shown in Fig 1B. The members within each subfamily showed similar exon/intron structures.

Subfamily I comprised 29 members (the largest number of members) with two or three exons. Subfamily II was formed by 17 members with two or three exons, except *GmGATA28*, which has one exon. Subfamily III was formed by 9 members with seven, ten, or eleven exons. Subfamily IV constituted of eight members with three, five, or eight exons. These gene structures of GATA factors are similar to those of *Arabidopsis* and rice [6]. *GmGATA* genes contained exons ranging from two to eleven in their CDS. The large variation in structures of soybean GATA factor family members could indicate that the soybean genome has changed significantly during its long evolutionary history. Several pairs of GATA proteins have a high degree of homology in the terminal nodes of each subfamily, suggesting that they are putative paralogous pairs. A total of 25 putative paralogous pairs were identified, with sequence identity ranging from 73% to 96% (S4 Table).

For the number of residues in the GATA zinc finger loop, most *GmGATA* genes encoded GATA factors with 18 residues (CX_2_CX_18_CX_2_C) that belonged to subfamilies I, II, and IV, whereas some encoded GATA factors with 20 residues (CX_2_CX_20_CX_2_C) that belonged to subfamily III. In addition, the zinc finger of the *GmGATA* genes of subfamilies I, II, and III was located at the carboxyl-terminal end of the protein, whereas that of subfamily IV was located at the amino-terminal end. These results are consistent with those in *Arabidopsis* and rice [6].

Similar to *Arabidopsis*, soybean contains subfamilies I, II, III, and IV but not rice-specific subfamilies V, VI, and VII. This result further confirmed the hypothesis proposed by [6] that subfamilies I, II, III, and IV appeared before the divergence between monocot and dicot, and that subfamilies V, VI, and VII evolved after the divergence between monocot and dicot or disappeared in dicot.

### Genome distribution and duplication of soybean GATA genes

**Fig 2:**
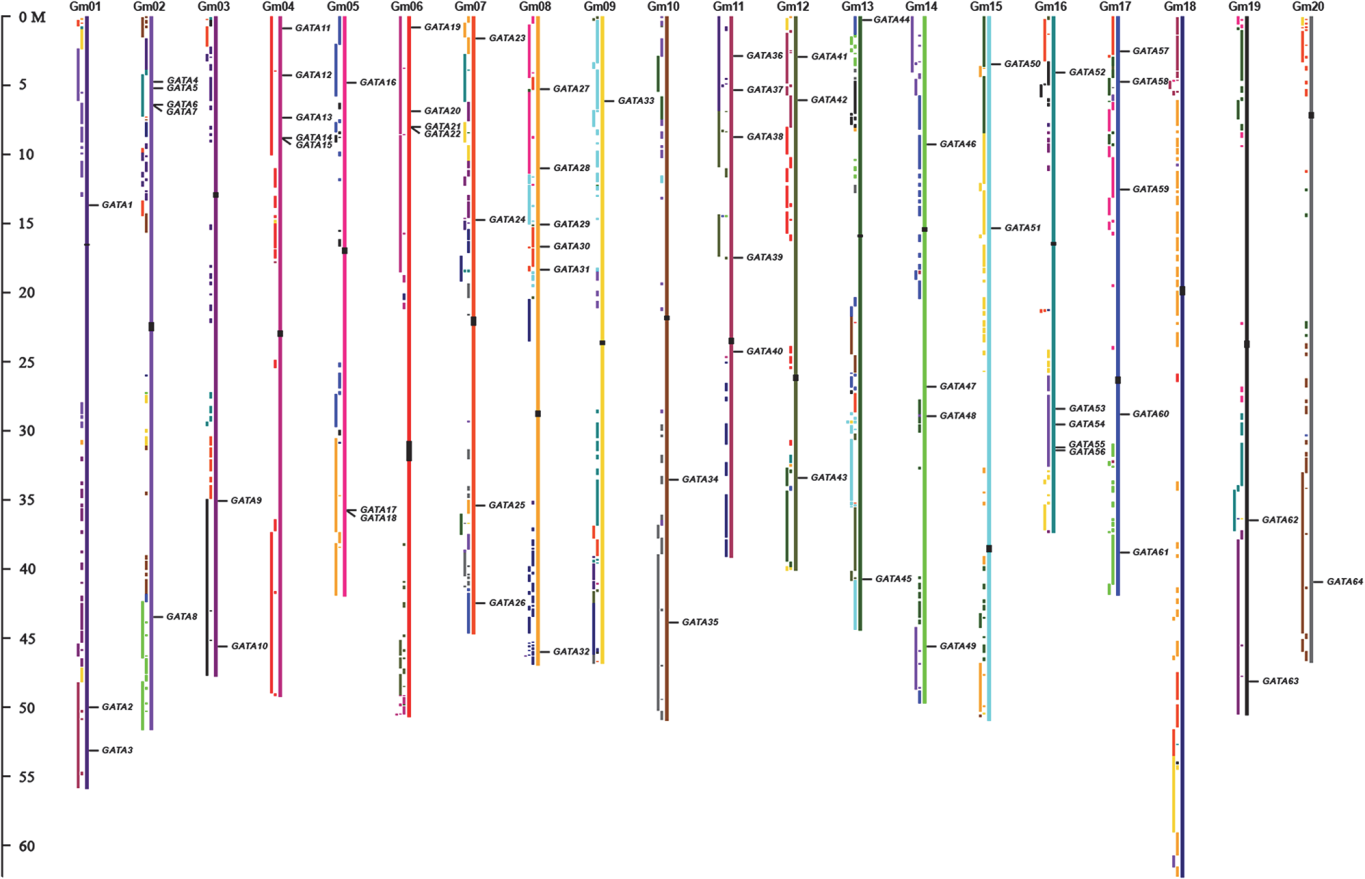
Chromosomal location and region duplication of soybean GATA factor genes. The schematic diagram of genome-wide chromosome organization and segmental duplication was made from the CViT genome search and synteny viewer at the Legume Information System (http://comparative-legumes.org). Colored blocks to the left of each chromosome show duplications with chromosomes of the same color. For example, the black blocks at the bottom of Gm03 correspond with regions on the black Gm19, and vice versa. Locations of centromeric repeats are shown as black rectangles over the chromosomes. The scale on the left represents the length of the chromosome.

The physical locations of the *GATA* genes on soybean chromosomes are shown in Fig 2. Sixty-four soybean *GATA* genes were unevenly distributed on all 20 chromosomes, except for chromosome 18. Among these chromosomes, chromosome 8 had the largest number of *GATA* genes with six, followed by chromosomes 2, 4, 11, 16, and 17 with five. By contrast, chromosomes 3, 10, 13, 15, and 19 had two *GATA* genes, and chromosomes 9 and 20 only contained one. Some clustering of *GATA* genes occurred on several chromosomes. For example, *GmGATA14* and *GmGATA15* were located in a 2.7-kb segment on chromosome 4, *GmGATA17* and *GmGATA18* were located in a 3.6-kb segment on chromosome 5, and *GmGATA21* and *GmGATA22* were located in a 2.2-kb segment on chromosome 6.

Gene duplication events are important for gene family expansion. Gene duplication may arise through several patterns, including segmental duplication, tandem duplication, retroposition, and transposition events [41]. Paralogous pairs located on the same chromosome either adjacent or separated by five or fewer genes were considered to be duplicated by tandem duplication. Paralogous pairs within known genomic duplication blocks were assigned as duplicates through segmental duplication [35]. A previous study showed that the soybean genome has undergone two rounds of whole genome duplication, including an ancient duplication prior to the divergence of papilionoid (58 Mya to 60 Mya) and a Glycine-specific duplication (13 Mya) [31]. The *GmGATA* genes were mapped to the duplicated blocks through CViT and synteny viewer at the Legume Information System (http://comparative-legumes.org/) to analyze the potential duplicate patterns of these genes during genome evolution. The distributions of soybean *GATA* genes relative to the corresponding duplicated genomic blocks are shown in Fig 2. Of the 25 putative paralogous pairs of *GmGATA* genes, 23 were located in segmental duplication blocks. Another two putative paralogous pairs (*GmGATA9*/*24* and *GmGATA47*/*60*) lacked the corresponding duplicates and were not located in the same chromosome. Therefore, no tandem duplication was found in the identified *GmGATA* genes. Nearly 72% of the 64 *GmGATA* genes were involved in the segmental duplication. This result suggested that segmental duplication significantly contributed to the expansion of the soybean GATA factor gene family.

### Conserved motifs outside the GATA domain

**Fig 3:**
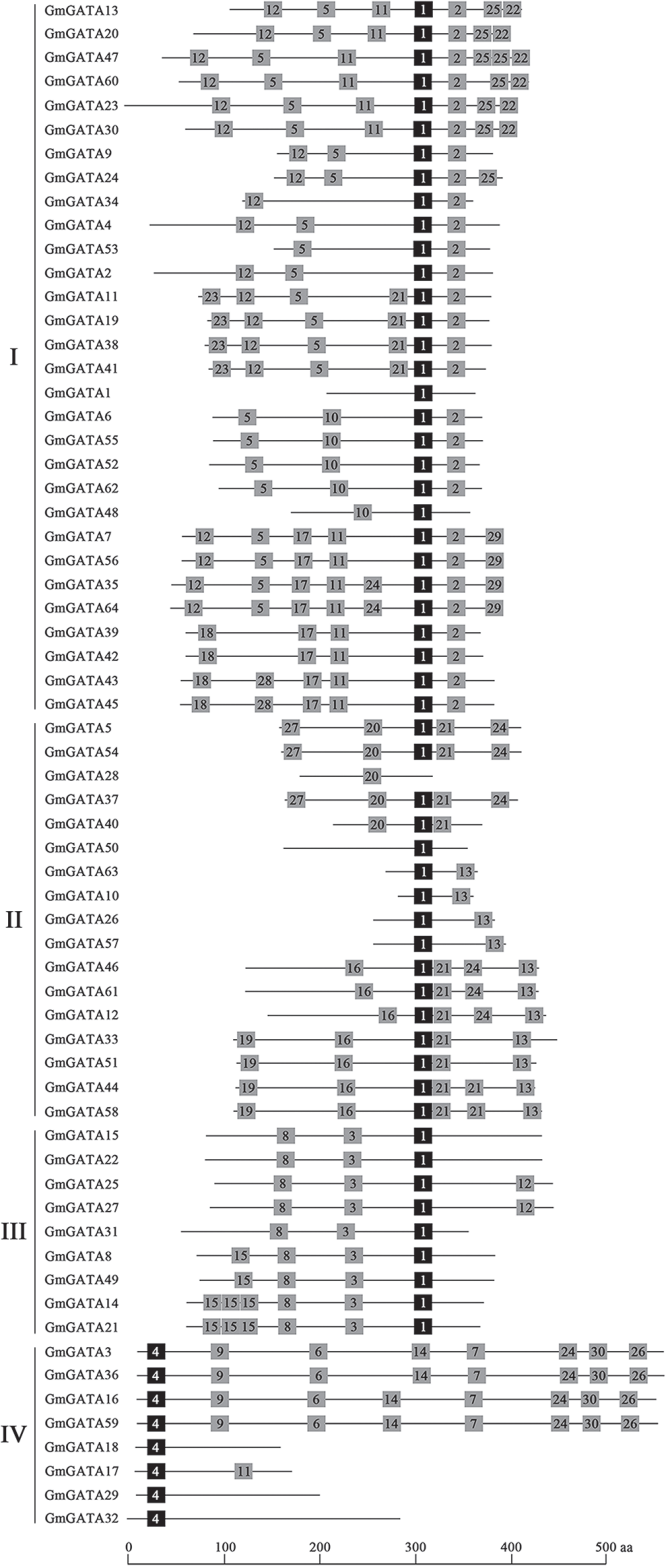
Schematic distribution of the conserved motifs in soybean GATA factors by MEME. Each numbered box represents a conserved motif in the protein. Motifs 1 and 4 represent the conserved GATA zinc finger motifs CX_2_CX_18_CX_2_ and CX_2_CX_20_CX_2_, respectively. Multilevel consensus sequences for the MEME-defined motifs are listed in S5 Table. The length of the protein can be estimated using the scale at the bottom.

To further reveal the diversification of *GATA* genes in soybean, putative conserved motifs were predicted by the program MEME, and 30 distinct motifs were identified in all 64 GATA proteins. The schematic distribution of the 30 motifs among the different gene subfamilies is shown in Fig 3, and the identified multilevel consensus sequence for the motifs is shown in S5 Table. Motif 1 present in 54 GmGATA proteins and motif 4 present in the other nine GmGATA proteins were the conserved GATA zinc finger domains CX_2_CX_18_CX_2_C and CX_2_CX_20_CX_2_C, respectively. The conserved GATA zinc finger domain was not found in GmGATA28 by MEME, which may be attributed to the small half GATA motif in GmGATA28. As expected, most of the closely related members in the same subfamily had common motif compositions. Motifs 2 and 5 appeared in nearly all members of subfamily I. Motif 21 was the conserved motif in subfamily II. Motifs 3 and 8 were specific to subfamily III. Motif 3 was annotated as the CCT domain. It was first discovered in transcription factor TOC1 and CONSTANS proteins, which are involved in plant photoperiodic signaling, and the CCT domain was implicated in mediating protein-protein interactions [42–43]. Motif 8 was annotated as the TIFY domain, which may be involved in jasmonic acid-related stress response and developmental processes [44]. The CCT and TIFY motifs are also conserved in the GATA factor members of subfamily III in *Arabidopsis* and rice. In subfamily IV, four closely related members contain motifs 9, 6, 14, 24, 30, 26, and 7. These similarities in motif patterns suggest the similar functions of the GATA factors in the same subfamily. The differences in motif distribution in the different subfamilies of GATA factors indicated the functional divergence of the GATA factors over evolutionary history.

### Evolutionary relationships among the GATA family in *Arabidopsis*, rice, and soybean

Given the high degree of diversity among the full-length GATA protein sequences, we analyzed the phylogenetic relationship of the GATA proteins in soybean, *Arabidopsis*, and rice on the alignment of the conserved GATA zinc finger domain, a region of approximately 55 residues (from amino acid −2 to residue +53 with respect to the first Cys) [45]. The amino acid sequences and subfamily information of *Arabidopsis* and rice GATA factors are available in S6 Table. For rice GATA factors OsGATA25 and OsGATA26 with two GATA domains, the N-domain is denoted by OsGATA25-N or OsGATA26-N, and the C-domain is denoted by OsGATA25-C or OsGATA26-C as previously described [6]. For rice GATA factors OsGATA24 with four GATA domains, the different domains are numbered from the amino- to the carboxy terminus (OsGATA24-1, OsGATA24-2, OsGATA24-3, and OsGATA24-4) [6]. GmGATA28, GmGATA48, OsGATA25-N, OsGATA26-N, OsGATA24-2, and OsGATA24-3 were excluded in the phylogenetic relationship analysis in this study because of the divergent domain.

**Fig 4:**
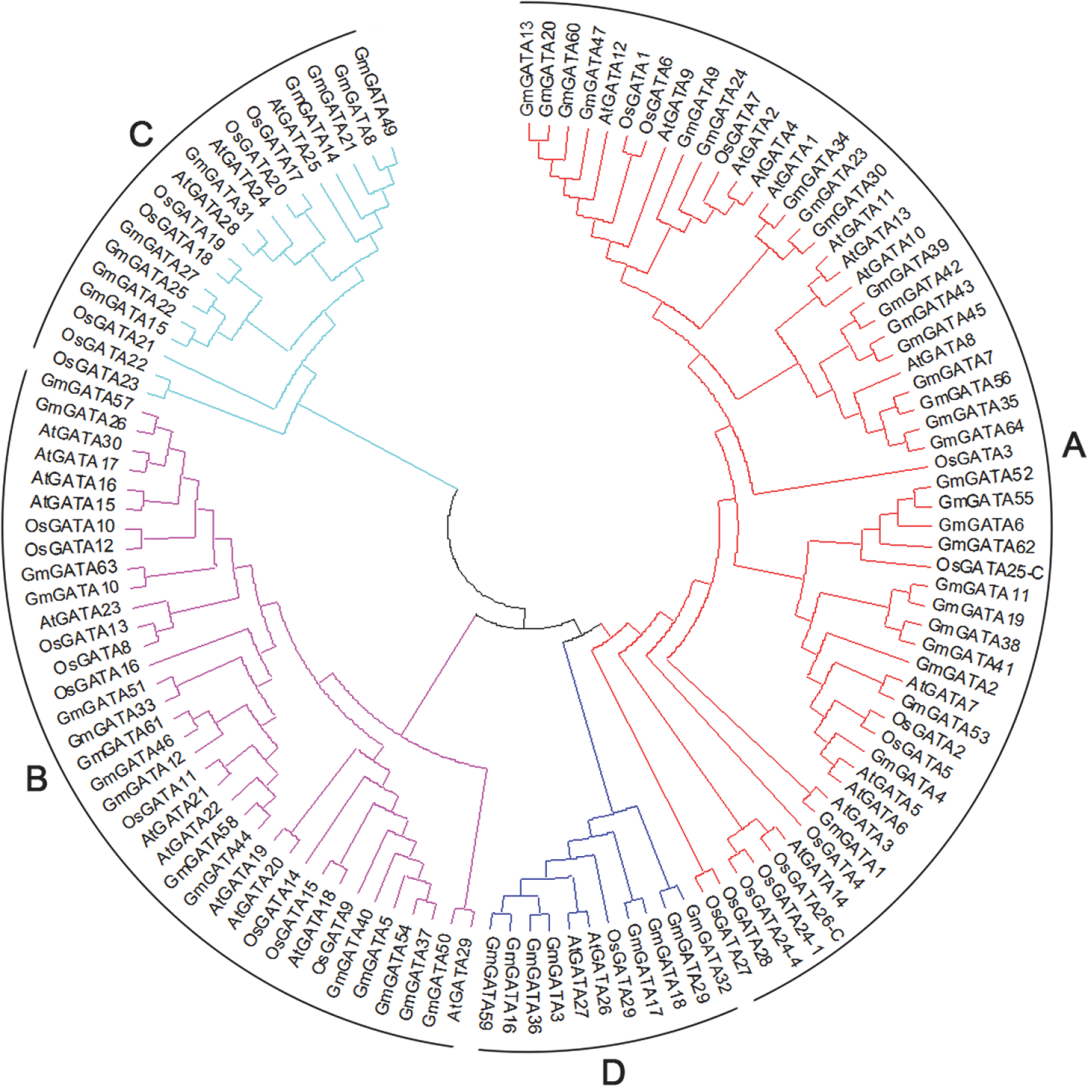
Phylogenetic tree of the amino acid sequences of zinc finger domains from soybean, *Arabidopsis*, and rice. The tree was conducted based on the zinc finger amino acid sequences using MEGA 5.0 by the neighbor-joining method with 1000 bootstrap replicates. The tree shows four major phylogenetic classes (Classes A to D) indicated with different colors.

The phylogenetic tree showed that all the GATA zinc finger sequences from the three higher plants were divided into four major clades (Classes A, B, C, and D) (Fig 4). This result is similar to that previously reported for *Arabidopsis* and rice [6]. Among these classes, Class A constituted the largest clade, containing 56 members and accounting for 46% of the total GATA zinc finger sequences, Class B formed the second largest clade containing 36 members and accounting for 29% of the total GATA zinc finger sequences, and the other two clades contained 19 (Class C) and 11 (Class D) members, respectively. The zinc fingers of the soybean GATA proteins from subfamilies I, II, III, and IV belonged to Classes A, B, C, and D, respectively. Similar results were obtained in *Arabidopsis* [6]. The GATA zinc fingers from three higher plants distributed interspersedly in all classes, suggesting that the expansion of GATA zinc fingers occurred before the divergence of soybean, *Arabidopsis*, and rice. Some putative orthologs, namely, *AtGATA1*/*GmGATA34*, *AtGATA7*/*GmGATA53*, *GmGATA1*/*AtGATA3*, *GmGATA31*/*AtGATA28*, and *OsGATA11*/*AtGATA21*, were proposed based on the phylogenetic tree.

In general, the GATA factors in the same clade may have similar functions. In Class A, nine soybean GATA factors (*GmGATA13*/*20*/*60*/*47*/*9*/*24*/*34*/*23*/*30*) clustered with the *Arabidopsis* GATA factors *AtGATA1*, *AtGATA2*, and *AtGATA4*, which are reportedly involved in the light regulation of gene expression and photomorphogenesis [16–17]. Eight soybean GATA factors (*GmGATA39*/*42*/*43*/*45*/*7*/*56*/*35*/*64*) clustered with the *Arabidopsis* GATA factor *AtGATA8* (*BME3*, *Blue Micropylar End3*), which functions as a positive regulator of seed germination [22]. In Class B, seven soybean GATA factors (*GmGATA51*/*33*/*58*/*44*/*12*/*61*/*46*) clustered with the *Arabidopsis* GATA factors *AtGATA21* (*GNC*) and *AtGATA22* (*GNL/CGA1*) and rice GATA factor *OsGATA11* (*Cga1*); these factors regulate chloroplast development, chlorophyll biosynthesis, starch production, plant architecture, and carbon and nitrogen metabolism [7, 27, 46–47]. Four soybean GATA factors (*GmGATA40*/*37*/*5*/*54*) clustered with the *Arabidopsis* GATA factor *AtGATA18* (*HAN*, *HANABU TARANU*) and rice GATA factor *OsGATA15* (*NL1*, *NECK LEAF1*); these factors are involved in regulating flower and shoot apical meristem development and organ differentiation during reproductive development [19, 21]. In Class C, nine soybean GATA factors (*GmGATA27*/*25*/*22*/*15*/*49*/*8*/*21*/*14*/*31*) clustered with the *Arabidopsis* GATA factor *AtGATA25* (*ZIM*, *Zinc-finger protein expressed in Inflorescence Meristem*); this factor is involved in hypocotyl and petiole elongation [20]. Understanding the phylogenetic relationship of GATA factors from soybean, *Arabidopsis*, and rice enables us to investigate the potential biological functions of soybean GATA factors.

### Tissue expression profiles of soybean *GATA* genes

**Fig 5:**
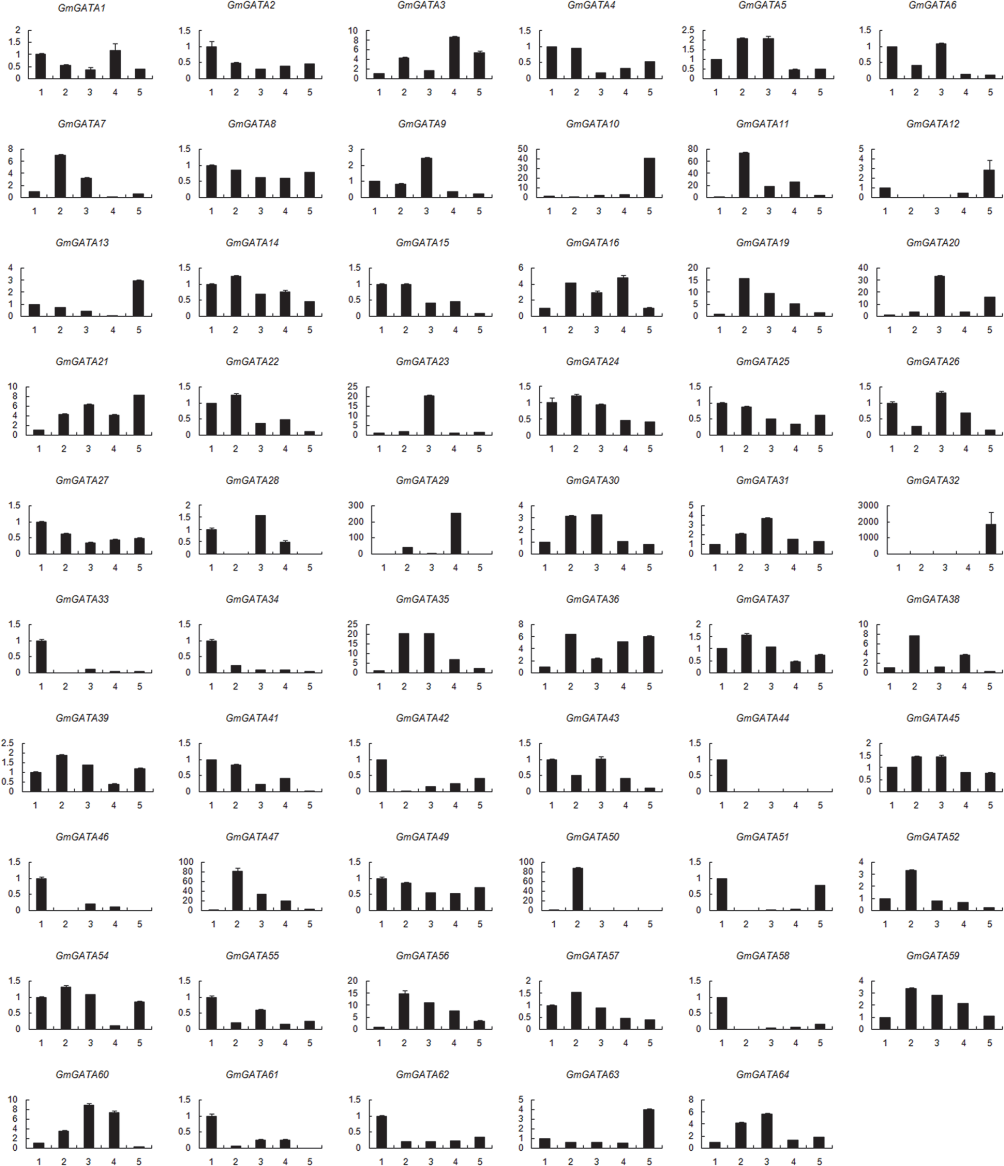
Relative expression profiles of soybean *GATA* genes in various organs. Data were obtained by real-time PCR normalized against the reference gene *ACT11* and shown as a percentage of expression in leaf. Numbers on the *x*-axis indicate various tissues: 1 (young leaf), 2 (root), 3 (stem), 4 (flower), and 5 (immature seed).

To identify the tissue expression patterns of *GmGATA* genes in soybean, specific primers were designed for each of the GATA factor genes (S1 Table), and the expression profiles of the 64 *GmGATA* genes were investigated in various tissues, including root, stem, young leaf, flower, and immature seed, by real-time PCR. Results showed that the soybean *GATA* genes were expressed in distinct patterns (Fig 5). The *GmGATA8*, *GmGATA45*, and *GmGATA49* genes showed less than twofold expression variation in different tissues, suggesting that they are not developmentally regulated at the transcription level. Some *GmGATA* genes were constitutively expressed in different tissues, but with preferential expression in certain tissues. For example, *GmGATA33*/*34*/*42/46*/*58*/*62* were predominantly expressed in young leaf; *GmGATA7*/*11*/*38*/*47*/*52* in root; *GmGATA9*, *GmGATA20*, and *GmGATA23* in stem; and *GmGATA10*, *GmGATA13*, and *GmGATA63* in immature seed. Moreover, *GmGATA29*, *GmGATA32*, *GmGATA44*, and *GmGATA50* exhibited a highly tissue-specific expression pattern in flower, immature seed, young leaf, and root, respectively. Among these four genes, *GmGATA44* having maximum similarity with the *Arabidopsis GATA* gene *AtGATA22* based on GATA zinc finger sequences (Fig 4) shared a highly similar expression pattern to *AtGATA22* [14], a regulator of chloroplast development and chlorophyll biosynthesis [7, 46]. The *GATA* genes highly expressed in specific organs of plants are crucial for the functioning or development of a specific organ.

In addition, four *GmGATA* genes showed no expression in one or two tissues. *GmGATA12* was undetectable in root and stem but highly expressed in seed; *GmGATA28* was not expressed in root and seed but moderately expressed in stem; *GmGATA29* and *GmGATA61* were not expressed in seed but highly expressed in flower and young leaf, respectively. Five *GmGATA* genes *GmGATA17*/*18/40/48/53* were not detected in any examined tissues. This result is consistent with the fact that no EST sequences corresponding with the five *GmGATA* genes were found in the Gene Indices at DFCI (Table 1). This result may be attributed to the insufficient sampling or the presence of untranscribed pseudogenes in the family. Genes within the same segmental duplicated pair usually have similar expression profiles. *GmGATA3*/*36*, *GmGATA6*/*55*, *GmGATA8*/*49*, *GmGATA10*/*63*, *GmGATA11*/*19*, *GmGATA15*/*22*, *GmGATA16*/*59*, *GmGATA25*/*27*, *GmGATA35*/*64*, *GmGATA44*/*58*, and *GmGATA46*/*61* were expressed at similar profiles, implying redundant functions. In addition, other segmental duplicated gene pairs (e.g., *GmGATA13*/*20*, *GmGATA23*/*30*, and *GmGATA33*/*51*) showed significantly different tissue expression profiles, implying divergent functions. Some members in the same subfamily shared a highly similar expression profile. For example, *GmGATA4*/*2*/*11*/*19*/*38/41* from the same clade in subfamily I showed predominant expression in leaf or root, and *GmGATA46*/*61*/*33*/*44*/*58* from the same clade in subfamily II had predominant expression in leaf. All these expression profiles suggest redundancy and divergence in the biological functions of soybean GATA factor genes during plant growth and development.

### Expression profiles of soybean *GATA* genes under low nitrogen stress condition

**Fig 6:**
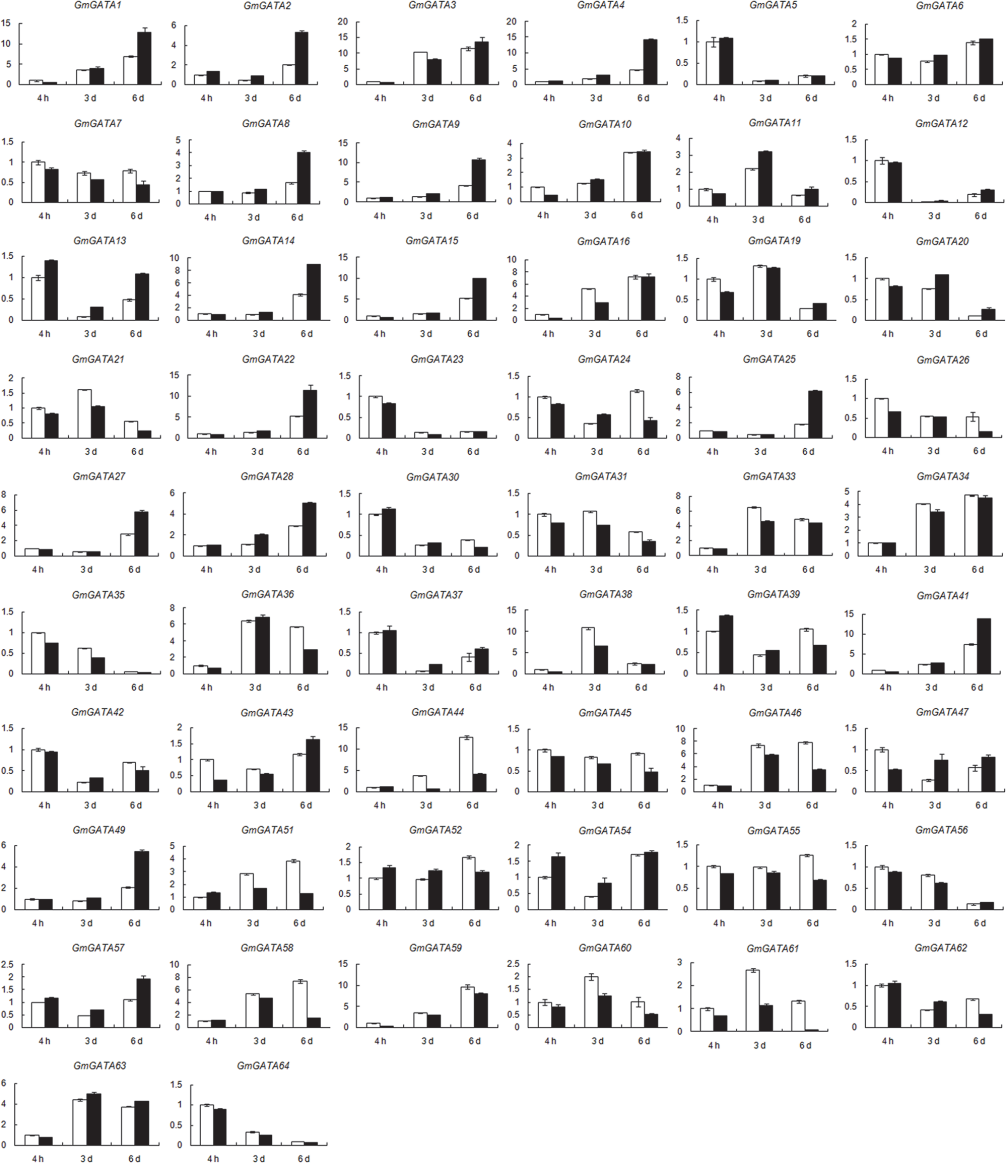
Expression of soybean *GATA* genes in leaves in response to low nitrogen stress. Data were obtained by real-time PCR normalized against the reference gene *ACT11* and shown as a percentage of expression in control leaves at 4 h. White column represents the expression under normal nitrogen condition, and black column represents the expression under limited nitrogen condition. Eight genes (*GmGATA17*/*18*/*29*/*32*/*40*/*48*/*50*/*53*) not expressed in soybean leaf under normal condition were not induced under low nitrogen stress and not present in this figure.

**Fig 7:**
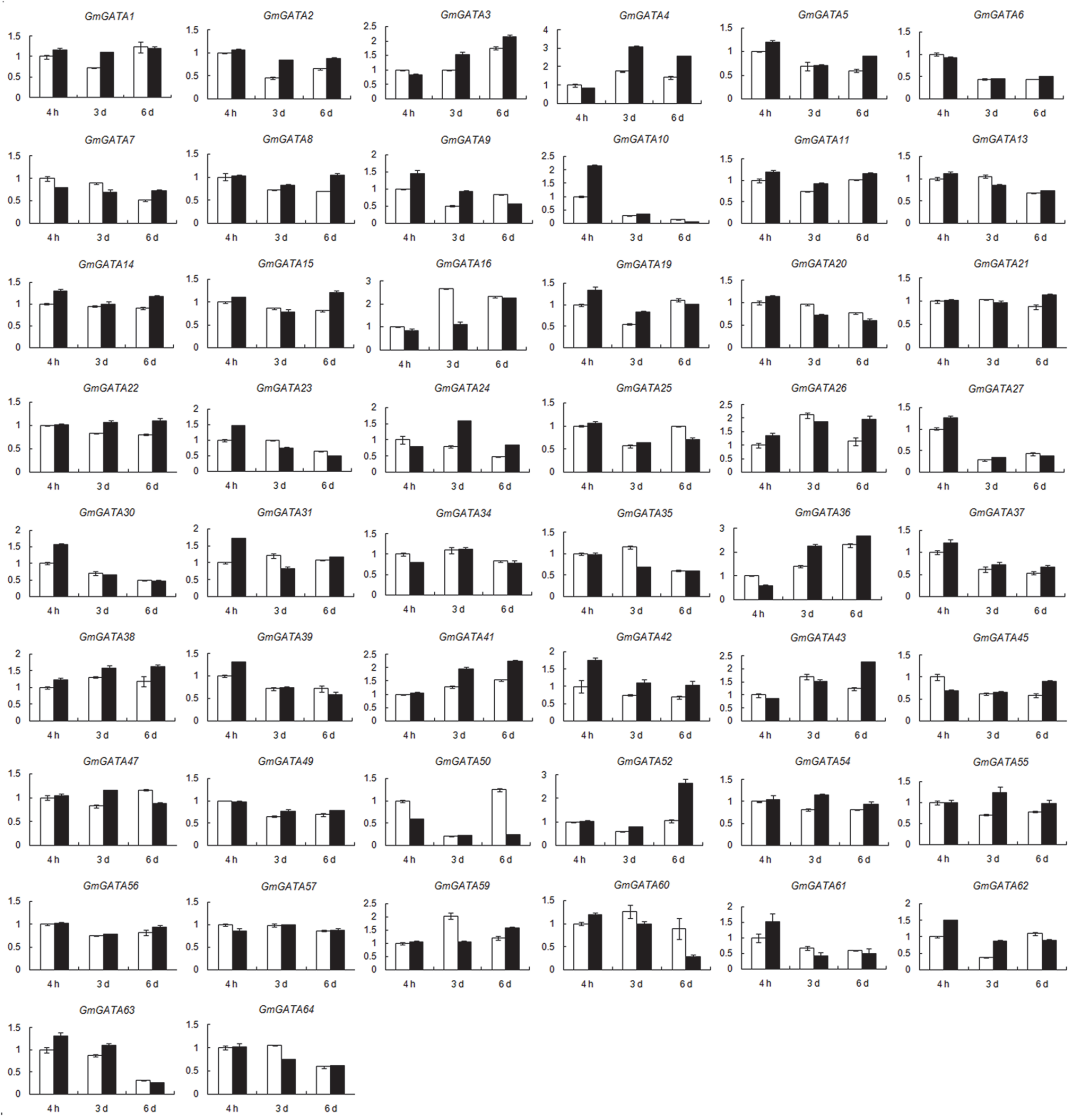
Expression of soybean *GATA* genes in roots in response to low nitrogen stress. Data were obtained by real-time PCR normalized against the reference gene *ACT11* and shown as a percentage of expression in control roots at 4 h. White column represents the expression under normal nitrogen condition, and black column represents the expression under limited nitrogen condition. Fourteen genes (*GmGATA12*/*17*/*18*/*28*/*29*/*32*/*33*/*40*/*44*/*46*/*48*/*51*/*53*/*58*) not expressed in soybean root under normal condition were not induced under low nitrogen stress and not present in this figure.

Previous studies showed that some members of the plant GATA factor gene family are involved in nitrogen response [7, 27, 48]. Therefore, we analyzed transcript abundance from low nitrogen solution-grown and half Hoagland solution-grown soybean seedlings by real-time PCR to determine whether or not the soybean *GATA* factor genes are nitrogen regulated. The expression data in leaf and root are shown in Figs 6 and 7, respectively. We compared the expression levels of *GmGATA* genes in these seedlings at 4 h, 3 d, and 6 d after treatment.

As shown in Fig 6, 26 soybean *GATA* genes were differentially expressed in the leaves of low nitrogen-treated seedlings compared with those of the untreated control seedlings, and most of them showed different expression levels at 6 d after treatment. A total of 12 genes showed significantly higher expression in the leaves of low nitrogen-treated seedlings than in those of the untreated control seedlings (Fig 6). The greatest differences were observed for *GmGATA25* (increased by 2.36-fold at 6 d after treatment), *GmGATA4* (increased by 2.05-fold at 6 d after treatment), and *GmGATA13* (increased by 2.64-fold at 3 d after treatment). Among the 12 differentially expressed *GATA* factor genes, six (*GmGATA2*/*4*/*9*/*13*/*20*/*47*) belonged to one clade of subfamily I, and the other six (*GmGATA8*/*14*/*22*/*25*/*27*/*49*) belonged to subfamily III. By contrast, 14 genes showed lower expression in the leaves of low nitrogen-treated seedlings than in those of the untreated control seedlings (Fig 6). The greatest differences were observed for *GmGATA61* (decreased by 58% and 95% at 3 and 6 d after treatment, respectively), *GmGATA44* (decreased by 81% and 67% at 3 and 6 d after treatment, respectively), *GmGATA58* (decreased by 79% at 6 d after treatment), and *GmGATA26* (decreased by 74% at 6 d after treatment). Among these 14 genes, half of them (*GmGATA10*/*26*/*44*/*46*/*51*/*58*/*61*) belonged to one clade of subfamily II, four (*GmGATA24*/*35*/*43*/*62*) belonged to subfamily I, one (*GmGATA21*) belonged to subfamily III, and two (*GmGATA16*/*59*) belonged to subfamily IV.

Some segmental duplicated gene pairs, such as *GmGATA8*/*49*, *GmGATA16*/*59*, and *GmGATA25*/*27*, shared similar expression change in leaves in response to low nitrogen stress. However, some pairs showed different expression profiles. For example, for *GmGATA14*/*21*, the expression of *GmGATA14* increased by 1.17-fold in low nitrogen-treated leaves compared with the control at 6 d after treatment, whereas *GmGATA21* decreased by 57%. For *GmGATA33*/*51*, *GmGATA51* decreased by 68% in low nitrogen-treated leaves compared with the control at 6 d after treatment, whereas *GmGATA33* showed no expression change in response to low nitrogen stress. Similar results were also obtained for *GmGATA26*/*57*, *GmGATA10*/*63*, *GmGATA43*/*45*, *GmGATA35*/*64*, and *GmGATA52*/*62*. These findings suggest redundancy and divergence in the biological functions of soybean GATA factor genes in response to low nitrogen stress.

Fewer differentially expressed GATA factor genes were found in soybean roots than in soybean leaves. Seven *GATA* genes (*GmGATA10*/*24*/*52*/*62*/*16*/*50*/*60*) showed significantly different expression levels between the roots of low nitrogen-treated and untreated control seedlings (Fig 7). The greatest differences were observed for *GmGATA52* (increased by 1.52-fold at 6 d after treatment compared with the control) and *GmGATA50* (decreased by 79% at 6 d after treatment compared with the control). Among these seven genes, four (*GmGATA24*/*52*/*62*/*60*) belonged to subfamily I, two (*GmGATA10*/*50*) belonged to subfamily II, one (GmGATA16) belonged to subfamily IV, and none belonged to subfamily III. Four *GATA* genes (*GmGATA10*/*16*/*24*/*62*) exhibited different expression levels in both leaves and roots compared with the control.

**Fig 8:**
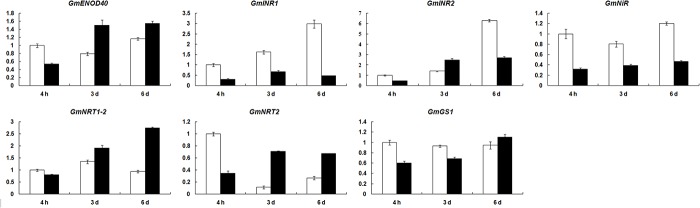
Expression of soybean nodulation and nitrogen metabolism-related genes in roots in response to low nitrogen stress. Data were obtained by real-time PCR normalized against the reference gene *ACT11* and shown as a percentage of expression in control roots at 4 h. White column represents the expression under normal nitrogen condition, and black column represents the expression under limited nitrogen condition.

To further analyze the correlation between the differentially expressed GATA factors and nitrogen metabolism-related genes in soybean roots in response to low nitrogen, a total of seven genes involved in nodulation (*ENOD40* [49]), preliminary nitrogen reduction (*INR1* [50], *INR2* [50] and *NiR* [51]), nitrogen transport (*NRT1-2* and *NRT2* [52]), and nitrogen assimilation (*GS1* [53]) were selected for real-time PCR assay. Results showed that the expression levels of *ENOD40* and *GS1* were not altered significantly in low nitrogen-treated roots compared with the control (Fig 8). The results indicated that the differentially expressed GATA factors were not associated with the nodulation specific gene *ENOD40*. *INR1*, *INR2* and *NiR* were all down-regulated after low nitrogen treatment, and *NRT1-2* and *NRT2* were both up-regulated (Fig 8). The correlation analysis between these soybean nitrogen metabolism-related genes and the differentially expressed GATA factors indicated that *NRT1-2* was co-expressed with *GATA52* in low nitrogen condition, as they were both up-regulated at 6 d after low nitrogen treatment. Moreover, *NRT1-2* contained the GATA binding domain in its promoter region (S2 Text). Whether GATA52 could interact with the promoter of *NRT1-2* and regulate its expression will be analyzed in the future. Additionally, *INR2* and *NRT2* also contained the GATA binding domain in their promoter regions (S2 Text). Whether some other GATA factors interact with the promoters of *INR2* and *NRT2* will be analyzed in our future study.

### *GmGATA44* modulates chlorophyll content

As previously mentioned, the expression patterns of *GmGATA44* and *GmGATA58* were similar to those of the *Arabidopsis* orthologs *AtGATA21* and *AtGATA22* and the rice ortholog *OsGATA11*. They are all inducible by nitrate [27, 48] and exhibit the strongest expression in green leaf tissues [14, 27, 47]. These findings indicate the functional conservation among soybean, *Arabidopsis*, and rice. *AtGATA21*, *AtGATA22*, and *OsGATA11* are involved in regulating chlorophyll synthesis and nitrogen metabolism [7, 27].

**Fig 9:**
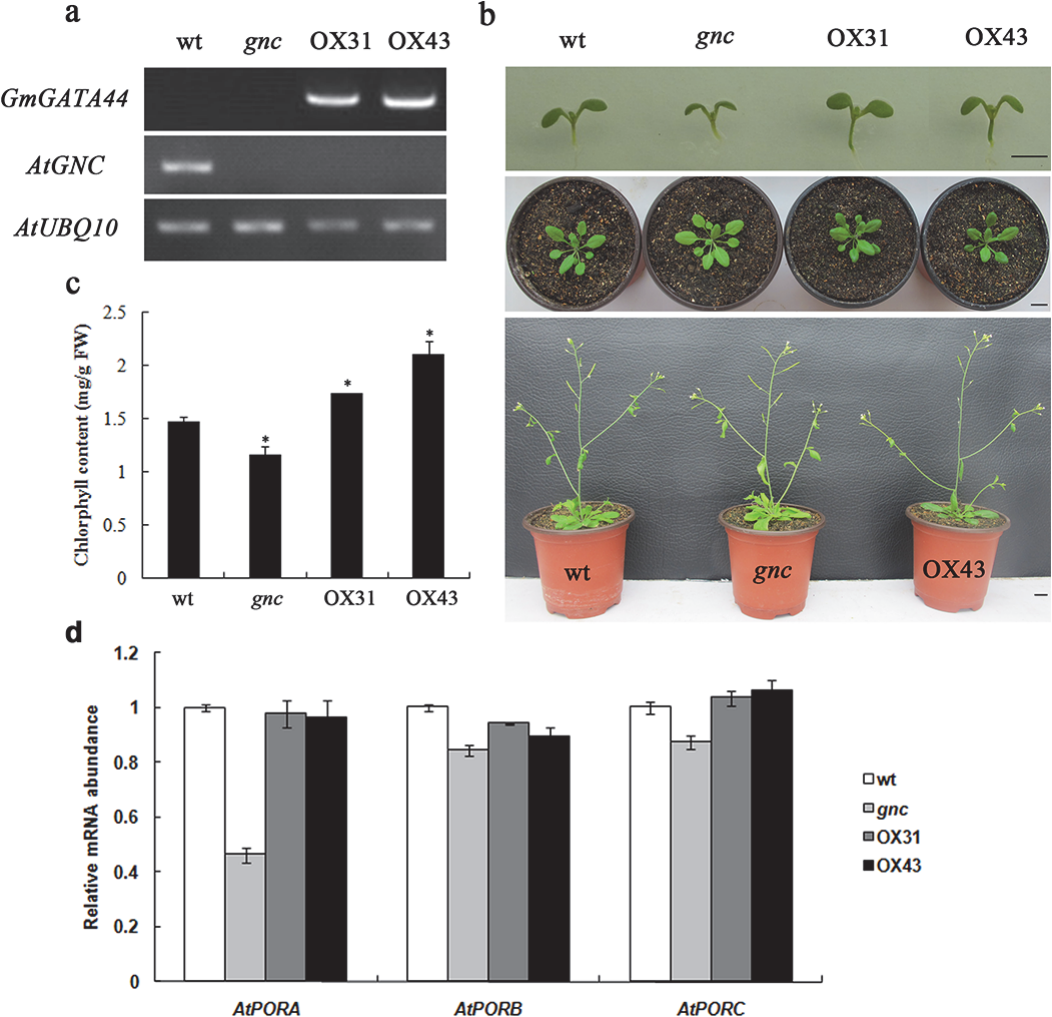
*GmGATA44* modulates chlorophyll content. (a) Expression levels of *GmGATA44* and *AtGNC* in the wild-type *Arabidopsis* (wt), the *gnc* mutant and two *GmGATA44* overexpressing transgenic lines (OX31 and OX43) using semi-quantitative RT-PCR from 3 week old rosette leaf tissue.(b) Images of the wild-type plant, the *gnc* mutant and *GmGATA44* overexpressing transgenic plants at one week (upper panel), 3 weeks (middle panel) and 5 weeks (bottom panel) post germination. Bars = 1 cm.(c) Chlorophyll content of the wild-type plants, the *gnc* mutant and two *GmGATA44* overexpressing transgenic lines at 3 weeks post germination. Data are presented as mean ± SD (N = 10) from triplicate independent measurements. Data analysis was performed using SAS software, and significant differences were calculated using the Student’s *t*-test at 95% confidence limit. Asterisk indicates significant differences from the wild-type plant.(d) Relative expression levels of *AtPORA*, *AtPORB* and *AtPORC* in the wild-type plant, the *gnc* mutant and two *GmGATA44* overexpressing transgenic lines by real-time PCR from 3 week old rosette leaf tissue. Data were obtained by real-time PCR normalized against the reference gene *GAPDH* and shown as a percentage of expression in the wild-type plant.

The *Arabidopsis gnc* mutant has a T-DNA insertion in the exon of *AtGATA21* gene, leading to the reduced chlorophyll phenotype. To confirm whether *GmGATA44* had similar biological functions of the orthologous gene *AtGATA21*, overexpression of *GmGATA44* under the control of CaMV 35S promoter was carried out in the *gnc* mutant background to complement this mutant. A total of 50 *GmGATA44* overexpressing (OX) transgenic plants were obtained, and two lines (OX31 and OX43) were chose for further analysis. Semi-quantitative RT-PCR results showed that the exogenous *GmGATA44* was abundantly expressed in both OX31 and OX43 lines, and the endogenous *AtGNC* was expressed in wild-type *Arabidopsis* rather not in the *gnc* mutant and two transgenic lines (Fig 9A). Both OX31 and OX43 lines restored pale green leaves of the *gnc* mutant to green and even greener leaves than that of wild-type plants (Fig 9B). The results of chlorophyll content in leaves also corresponded to this complementation. The chlorophyll accumulation was improved significantly in both OX31 and OX43 lines, compared to the *gnc* mutant, even more than that of wild-type plants (Fig 9C). In addition, strong accumulation of chlorophyll was also obviously observed in the seedling hypocotyls of both OX31 and OX43 lines (Fig 9B).

Changes in chlorophyll contents indicated that genes involved in chlorophyll biosynthesis might be altered. Consistent with the previous report [54], the expression levels of *AtPORA*, *AtPORB* and *AtPORC* were reduced in the *gnc* mutant compared with the wild-type plants (Fig 9D), which had been suggested to be the molecular cause for the greening defect of the *gnc* mutant [54]. Overexpression of *GmGATA44* in the *gnc* mutant led to the up-regulation of these *POR* genes, especially for *AtPORA*. Moreover, it should be noted that the expression level of *AtPORC* was increased slightly more than that in the wild-type plants. Additionally, other 14 genes involved in tetrapyrrole pathway [55] and two key genes (*AtDXS* and *AtDXR*) in methylerythritol phosphate pathway [56] for chlorophyll biosynthesis were also analyzed, and they were not found to be altered significantly in the two overexpressing lines compared with the *gnc* mutant (S2 Fig).

These results suggested that *GmGATA44* played an important role in modulating chlorophyll biosynthesis, similar to the function of the ortholog *AtGATA21*. Chlorophyll level is often used as a reflection of nitrogen status. The response of transgenic plants to low nitrogen stress will be analyzed in the further study.

## Conclusion

We identified 64 *GATA* genes in soybean through a genome-wide analysis. The soybean genome had more *GATA* genes than the *Arabidopsis* or rice genome. The great expansion of the soybean GATA factor gene family was likely due to segmental duplication during the evolutionary history. An overview of the soybean GATA factor gene family was revealed through the comprehensive investigation of their chromosomal distributions, gene structures, duplication patterns, phylogenetic tree, and conserved motifs. A comparative analysis of the GATA factor gene family across soybean, *Arabidopsis*, and rice helped us facilitate further gene function analysis of soybean *GATA* genes. Our results also provided useful information by identifying candidate tissue-specific and low nitrogen stress responsive soybean *GATA* genes. The preliminary function analysis showed *GmGATA44* had the similar function in modulating chlorophyll biosynthesis with its orthologs in *Arabidopsis* and rice. These investigations and analyses could increase knowledge on the functions of soybean *GATA* genes in the regulation of soybean growth and nitrogen metabolism.

## Supporting Information

S1 Text A complete list of 64 *GATA* gene sequences identified in the present study. The sequences are retrieved from the Phytozome or NCBI database.
(DOC)

S2 Text Regions of the *INR2*, *NRT1-2* and *NRT2* promoters containing the GATA binding domain. (DOC)

S1 Fig Amino acid sequence alignment of soybean GATA zinc finger domains. The 55-amino acid regions of 63 soybean GATA domains and the 29-amino acid regions containing the half GATA domain of GmGATA28 were aligned. Residues conserved in all or most of the soybean GATA domains are highlighted. Asterisks indicate the conserved cysteine residues (Cys) in the GATA domain.
(TIF)

S2 Fig Relative expression levels of 14 genes in tetrapyrrole pathway and two key genes in methylerythritol phosphate pathway for chlorophyll biosynthesis in the wild-type plant, the *gnc* mutant and two *GmGATA44* overexpressing transgenic lines by real-time PCR from 3 week old rosette leaf tissue. Data were obtained by real-time PCR normalized against the reference gene *GAPDH* and shown as a percentage of expression in the wild-type plants.
(TIF)

S1 Table Primers for the real-time PCR of soybean *GATA* genes and the semi-quantitative RT-PCR analysis of *GmGATA44* and *AtGNC*. (DOC)

S2 Table Primers for the real-time PCR of some nodulation and nitrogen metabolism-related genes. (DOC)

S3 Table Primers for the real-time PCR of some chlorophyll biosynthesis-related genes. (DOC)

S4 Table Pairwise identities between homologous pairs of soybean GATA factors. Pairwise identities and amino acid sequence alignments of the 25 homologous pairs identified from the soybean GATA family.
(XLS)

S5 Table Multilevel consensus sequence identified by MEME among soybean GATA factors. The motif numbers correspond to those described in Fig 3.
(XLS)

S6 Table Information of GATA factors from *Arabidopsis* and rice used for phylogenetic analysis. The GATA factor sequences of *Arabidopsis* and rice were obtained from the NCBI and rice genome annotation databases (http://rice.plantbiology.msu.edu/; release 7.0), respectively. The nomenclature is according to previous reports [6, 14].
(XLS)
