# Supplementary material for: Genome-Wide Survey of the Soybean GATA Transcription Factor Gene Family and Expression Analysis under Low Nitrogen Stress
Source: PLoS One. 2015 Apr 17;10(4):e0125174. doi: 10.1371/journal.pone.0125174 (PMC4401516; doi:10.1371/journal.pone.0125174)
Supplement: S1 Text — The sequences are retrieved from the Phytozome or NCBI database. (DOC) [file pone.0125174.s001.doc]

The coding sequences:

>GmGATA1

ATGGTTATCGCAAATTATGGGTTCCTAGAACACCCACTTTGTGTCCCTCAGGACTCATTAGAATGTTTGGGAATGATGAATTGGGAAGGCATGGATTCAATTGATTCCATGTTTTCTACCCCTTGGGAGAGTGAAAAGGAAAGATTGGAACAACCTGAAAAGGATAAAACGGAGAGGAAATCATTCACTGATAGTGGGAGGGATGCTAAGATCTGGGAGAAAAGATGTGGTCACAAGGATGCTAGGATCTGGGAGAGAAGATGTAGTCATTGTGACGCCATAAAAACTCCTCAATGGAGAACCGGTCCTTTTGGGCGAAATACTCTGTGCAATGCTTGTGGTATACGATTCAAGGCAGGGAAGTTATATCCAGAGTATAGACCTGCAGATAGTCCAACGTTTGATGTAAGCAAGCATTCAAATGTTCATAAAGAGATAATGAAAATGAGAAACCACCTTAGTTAA

>GmGATA2

ATGCTTTACCAAACTCCCTATCCTCAACCCTTTCAATTTCATCATCCTTTGCCTTCTTCCTTCTCCCCTCTCCTTGCAGTTCCAACCACACCACCTCCTCTCTATCTCCCTTTCCCTCAGGCCGAAAAAGAAATGGAGTGCGTGGAGGCAGCGTTGAAGAGCAATTATAGGAAAGAAATGACCTTAAAACTGAGCCCACAAACCTTCACGGAAGAGGTTAGTGTCCAAAACGGCACAACCTGCGACGACTTTTTCGTTAACGACCTCCTTGACTTCTCTCACGTCGAAGAAGAACCCGAACAACAAGAAGACACTCCTTGTGTCTCCCTTCAGCATGAAAACCCAAGCCACGAGCCCTGTACTTTCAAAGACGATTATGCCTCTGTGCCCACTAGCGAGCTTAGCGTCTTGGCGGATGACTTGGCGGACTTGGAGTGGCTGTCTCATTTCGTGGAGGATTCCTTCTCGGAATTCTCTGCGGCCTTCCCCACCGTAACGGAGAACCCAACAGCATGCCTTAAAGAGGCAGAGCCTGAACCGGAAATCCCGGTTTTCTCTTTCAAAACCCCGGTTCAGACCAAAGCAAGAAGCAAGCGAACCAGAAACGGTCTTCGTGTTTGGCCATTCGGTTCACCTTCCTTCACCGATTCTTCCTCAAGCTCCACCACCTCTTCCTCTTCCTCTTCTTCACCCTCTAGCCCCTTGCTAATTTACACCCAAAGCCTCGACCACCTGTGCTCCGAGCCAAATACCAAAAAAATGAAGAAAAAACCCTCTTCCGATACCCTGGCGCCGCGAAGGTGCAGTCATTGCGGCGTGCAGAAAACCCCTCAGTGGAGAACTGGGCCGCTAGGCCCAAAAACACTATGCAACGCGTGTGGGGTCCGCTTCAAGTCGGGCCGGCTCTTACCCGAATACAGGCCTGCTTGTAGCCCAACATTTTCAAGTGAATTACACTCGAACCATCATCGTAAAGTGCTCGAGATGCGGCAAAAGAAGGAGACAGTTTCTGTGGATGAGACTGGTTTTGCCCCTGCTCATGTTGTTCCCAGTTTTTGA

>GmGATA3

ATGGGCAAGCAAGGGCCTTGCTATCACTGTGGAGTTACAAGCACACCACTCTGGCGCAATGGACCACCAGAGAAGCCAGTACTATGCAATGCATGTGGGTCTCGATGGAGGACAAAGGGAACTCTTGCGAAATATACCCCTTTACATGCTCGAGCAGAAACTGATGATTATGATGATCAAAGGGTTTCCAGGGTAAAGAGCATATCGATAAATAAGAAGAAAGAAGTGGCATTGCTCAAACGAAAACAGAACCATGATAATGTAGTATCTGGAGGGTTTGCACCTGATTACAACCAGGGATACCAGAAGGTCGTAGATGAAGATATAAGCAACCGATCAAGTTCAGGATCAGCTATCTCTAACTCAGAGAGCTGTGCACAATTTGGTTATGGTGGCATGGATGCTAGTGATCTGACAGGTCCTGCTCAGTCAGTGGTCTGGGATGCCATGGTGCCTTCAAGAAAGAGGACATGTGTTGGTCGTCCAAAGCCTTCTTCTGTTGAGAAGCTAACAAAAGATTTATGTACTATTCTTCATGAACAACAGTCATATTTTTCGGTATCTTCTGAAGAAGATCTTCTTTTTGAAAGTGATACACCAATGGTCTCTGTTGAGATAGGACATGGAAGCATTCTCATTAGGCATCCTAGCTATATAGCTCGTGAAGAAGAGTCTGAGGCTAGCTCTCTTTCGGTTGATAATAAACAATGCCCAATGAGTGAGGCATATTCTTTTTCTGGTGCCATTGCAATGCATAATGATTCCAGTCGCTTGAAGTCATCATCTCTGGAAGTTGAAAAGATTGGGAACTCTACTGGCCAAGGAATGCAGCAGGAACAACTTAAAAGTGACAAGTCTCAACTTGAAAGAGTACAAATCCTAGGCAATCATGAATCCCCATTGTGCTCAATAGATTTAAATGATGTTGTAAACTATGAAGAGTTTTTGAGAATCTTGACAAATGAAGAGCAACAACAATTACTGAAGTTACTTCCTGTGGTTGATACTGCTAAACTCCCTGATAGCCTTGAAGTCATGTTCAGTAGCTCTCAATTCAAGGAGAACTTAACTTACTTTCAGCAGCTTCTTGCGGAAGGAGTCTTTGATATCTCTTTGTTGGGCGCAAAATCTGAAGACTGCAAGATTTTGAAAAGACTTGCATTATCCAATCTGTCAAAGTCAAAATGGGTAGCGCACCATAATTTTCTCAAGAAATGTAAAAACAAAGCTGGAAAATCTAATACTATGGGATCTACTGGTACAACATCAACTAATGTTTTGAACAACAGGGCGTCAACTGATGTTGCAAACATCAAGAGAATGCGTGACAGCAGAAATCAAAACTTACCAGAAATAAAGACAATAATGAGAAGCCCCAAAAGAACGATCGCAAAGGCGAGCTGTGAGGGCAAAGAAGCTGTAGAAGATGGTGCTTGCTATAGTCCAAAACACCTATTTGCTTTGCCTCCTGATGCTAGTTTTCTCTTGCTGGATTCCTTAAACTTTGTTCAGGAGAGTAGTGATCAGGATCTGCTGCTAGAGGTGTCATCTAACACTTCTTTTCCACAGGCAGAGCTCTTGCAGCCAACTTTAAGCCTTGGTGCTCAAGCCAGCACTAGTAGCAGCTCAATCTACTCAAATCTTGTTCACCATTAA

>GmGATA4

ATGAAAGAAAGAGGCACATTTCAGCCCTTATTCAATGCTTTACCAAACTCACTCATCATCCTCCAGTCTTTCAATTTCATCCCTTCACTTCTTTCCACAACCCCATCATCTTTTCCCTCTTTTCTTCTCTCTCAGGCGGAGAAAGAAATGGAGTGTTTGGAAGCAGCGTTGAAGAGCAGTTTCAGGAAAGACATGGCCCTCAAACAAACGTTGTTCCTAGAGGAGTTCTCGTCCGCCTCTAACGTTCAAAACGTCGTCGCTTCCTCCGACGACTTGTTCGTTGACGACCTCCTCAACTTTTCACTCCTCGAAAACAACACCAACAACAATAACAACAACGAAGAACCCGACCAACAACTCAACAACCACGACTCCACCACCCCTCAGAACAACCAGGAAAATTACAATTATAACCCCTCCTTCAACGACAACAATTTCAACACCGAACTCACCGTTCCGGCGGAGGAGGAAGTTGCGGACTTGGAATGGTTGTCTCGTTTCGTCGAAGATTCTAATTTCTCGGAATATTCTCTTCCCTTCCCCGCAACCGTGACTGAGAAAGTGAAAGTGAAATCACCGGAACCGGGAAATACCGCTTTCACTTTCAAAACCCCGGTTCCGGCCAAGGCCAGGAGCAAGCGAACGCGAACCGGTGTCCGGGTTTGGCCGCTCAAGTCACCCTCTTTGGCCGCCGCGTCTTCAACAACAACCTCGTCCTCGTCCTCGTCTTCGCCCTCGAGTCCGCAACGTGCGGACTCGAGGGCGAAGAAGCGTGCCGCTGCGGACGGCGGCGCGGCGCGACGGTGCAGCCACTGTGGCGTTCAGAAAACGCCGCAGTGGCGCACCGGACCGCTCGGGGCTAAGACTCTGTGCAACGCGTGTGGGGTCCGCTACAAGTCGGGTCGGTTATTACCCGAATATAGACCCGCCTGTAGCCCTACTTTCTCCAGCGAGTTGCACTCCAACCACCACCGAAAGGTCCTCGAGATGCGGCGGAAGAAGGAGGACGTGCCTGAGCCCGACACCGCTTCGCCACCGTCGCTTCCCGGTTTTTGA

>GmGATA5

ATGATGCATCATTGTTGTGGAAGCTCACAGGGGCACGTGATGGGCACTTGCACATGCGGCATGTATCACAATCACAATAGCAGCGAAGCCAGCTCTTATGGGTCGATGCTGTTTTCTATGCCCAACAACAACGAATATTACCAAGAACACGACATCTATTCTTCCTTCACGCCCTCTCATTCCTCCGTCGACTGCACGCTCTCCCTCGGAACCCCCTCCACGCGTTTAACCCAAGACGAGGACGACAACAAACGACACCGTCACCAACGTCGTTCTGGTGTCACCAGTTTTTGCTGGGATCTGCTCCATTCCAATCACAACAACAACAACATCACGCAATCTCAAAGCAAGTCTAGCAGCAGAGGAAGCAACAACAACAACGACTCTCTCCTCGCTCGTCGTTGCGCTAACTGCGACACCACTTCCACTCCCCTCTGGAGGAACGGTCCTCGTGGCCCAAAGTCACTATGCAACGCTTGCGGGATCAGATTCAAGAAGGAACAGAGAAGAGCGAGCGCCGCGGGGGCCACGTCAGCGTCAGCGGCGGTTCCCGGCGGCGCAATGGAATCGGCGCGCGTGTACGGCCACCACCACAACAATTCATGGTACGCACACTCTCAGAGCCAGAAGATGATGGGTAACGAGTTACGCTTCATGGATGATTCCGATGACAGAGACTCGGATAATAATGGCATTCCGTTCCTCTCTTGGAAACTCAACATTCCAGATCGAACGAGCCTGGTTGATGAACGATGGTGA

>GmGATA6

ATGGATGTTTGCCGAAATGTATCAGTTTCTTCAAGTGAGTGTCAGCAAGAGCTTCCCACCCTTGATGACCTTTTCTCTCATCAGAACACGGAAGTGGATTTTGGCCTGGAATGGTTATCAGTGTTTGTGGAAGACTGTTTCTCTAGTAGGCCAAGCTGCCTCTTGGCACCTGGTGGTGTTCAAACCACAAGCACTAGCACAAGCACCAAGCCTTCTTCAGGCACTATATTGCAAAGACCCCAACAATTAAGTCATCACTGTCCTTTGCAGAATTTTGCTGTGCCAGGGAAAGCAAGGAGCAAGAGAAAGAGAAAGAGGCTTTCAGCCCCTAGAACAACAAAACACACCCTAAGCACATGGTCGCAACATTTCAGCACACAGAATGATGGGGTGAGTTCTGACCCTCCTCTTTTGAAACAAGCTTATTGGTTGGCTGATAGTGAACTCATTGTGCCTAAGAAGAAGGATGTTGAGCAAGAAGAGGAGGAGGGGGTTGTTGTGGTTGTGAAGAAGGAGAAATTGGGGGATTATTGTGATCATGATGAGGGTGATGAAATTAACAATAATAATAGTAATAATGATGATAATGTGCAGCACCCAATTCCAAGAAGGTGCACACATTGTCTGGCTCAGAGGACCCCACAGTGGAGGGCAGGACCATTGGGTCCAAAGACACTATGCAATGCATGTGGGGTGAGGTTCAAGTCTGGAAGGTTGTTGCCAGAGTATAGGCCAGCCAAGAGCCCTACTTTTGTAAGCTATTTGCATTCCAATTCACACAAGAAAGTCATGGAGATGAGAATGGGTGTTGTTGGTGTTTTTTCTACAGACAACAACAAGTAG

>GmGATA7

ATGATTGGAAACTTCATCGACGACATTGACTGCGGCAGCTTCTTCGACCACATCGACGACCTCCTCGAATTCCCCGACGACAACGCCGCGCCGGTCGCTCCTCCGGCGAACTTCTGGTCCGCCGAGTCCGACTCGCTCCCCGCCTCCTACACGGTGTTTTCCGACAACTCCGTGACGGACCTCTCGGCGGAGCTCTCTGTTTCGTATGACGACATAGTGCAATTAGAATGGCTGTCCAATTTTGTCGAGGACTCTTTCTCTGGTGGGAGCATCACCATGAAGAAAGAGGAGGAGCCACAATGCACCACCACCACCAAAGAGGACATAGCTCATGCCCAATTTCAGACAGCAAGCCCGGTCTCAGTCCTTGAAAGCAGCAGTTTCTGCTCCGGCGAGAAGGCTGCATCTCGCGGCCCGGAGATTTACATCCCCGTGCCATGTGGGCGTGTGCGCAGCAAGCGCCCACGCCCTGCAACCTTCAACCCCCATCCAGTGATGCAGCTAATCTCCCCTGCATCCTCCACTGGCGAGAACGTGCAGCACAACGCCACCACCACTTCCAAGGCGGCCTCATCAGATTCCGAGAATTTTGCCGAGTCAGTGATCAAGGGGCCAAAGCAGGCCTCTGGGGAGCACAAGAACAAAAGGAAGATCAAGGTGACTTTCTCATCAGGTCAAGAGCAGCAGAATGCACCATCACAGGCAGTTAGGAAATGCTTGCACTGTGAGATAACCAAGACACCACAGTGGAGGGCAGGGCCAATGGGGCCGAAAACACTCTGCAATGCTTGTGGCGTGCGCTACAAGTCAGGCCGGCTTTTCCCCGAATACCGCCCTGCAGCAAGCCCAACATTTTGCGCGGCCGTGCACTCCAACTCCCACAAGAAGGTTATTGAAATGAGAAACAAGACAGGCACCAAATCTGGCTTTGCAACTGATTCTGCTGCCTCACCAGAACTCATTCCAAACACTAACAACAGCCTTACCCTTGAATACATGTGA

>GmGATA8

ATGGAGCCATCAGCAATGTACGGACCCTCCCAGCCCCTGAACATCCCCTCGCGGATCGGCGCTGGCGAAAGAGACGACGGCTCCGGCAACGAGCCCGCCGTCGACGGCCACCACCACCACATTCAGTACGAAACGCACGCGCTCGATGACGGTGCCGCCGGTGGCGCCGTGGTTGTCGAGGATGTTACGTCGGACGCGGTCTATGTCTCCGGCGGTGGAGGCCCCGAGGAGTCCAGCCAGCTTACGCTGTCGTTTCGTGGCCAAGTTTACGTCTTCGATGCCGTTACGCCTGATAAGGTTCAAGCGGTGTTGTTACTTTTGGGTGGATGTGAACTATCTTCGGGTGGTTCGCCATGTGTGGATCCTGGGGCTCAACAGAATCAAAGGGGTTCAATGGAATTTCCTAAATGTAGTCTACCACAACGAGCTGCCTCATTAGATAGGTTCAGGCAGAAGAGGAAAGAGCGATGCTTTGATAAAAAAGTGAGATATAGCGTACGGCAAGAAGTTGCGCTCAGGATGCATCGTAACAAGGGTCAGTTTACTTCATCTAAGAAACAAGATGGAGCTAATAGTTATGGTACTGACCAAGATTCAGGACAGGATGATAGTCAATCTGAAACCTCATGTAAACATTGTGGCACTAGTTCAAAATCCACCCCAATGATGCGGCGAGGGCCATCTGGTCCAAGGTCACTTTGCAATGCTTGTGGGCTTTTTTGGGCAAATAGGGGTGCTTTGAGAGACCTTTCTAAGAGAAATCAGGAACACTCTCTTCCACCAGTTGAACAGGTTGATGGAGGTAATGACCCAGACTGTCGGACTGCCGCTGCCGACCCTGCACAAAACAATCTTGCTGCTTTCTCAGAGCCTGTTAATCCAGCTTTGGTAGCTGATCGTAAGGTTTTCCAATCTCAGAAAATGTTGGAGTAG

>GmGATA9

ATGGACTTGTACGGTTCCTTTTCCACCCCTTCAGATTGCTTACACATCGATGATTTTCTAGATTTCTCCAACATCACCACCACCACCACCGACACCCACCACCACTTTCCTCCGCCGCAAAACTCTCCATCAATCTCCCACGATCCCAATTTCTTCCTCAATTTCCCTTCCGTTCCGAGCGACGAAGCAGTGGAGCTGGAGTGGCTCTCCCAGTTCGTGAACGACGAGGCGACGTCGTTTCACAACATCCCACCACCCGCATCCATTGGATCCCACACGACGCCGTTTCTTTCCAACAATAACAGGAACGATAATAATAACGAATATCCCAAATCATCATCATCTTCACCGGTGTTGGCGGGAAAATCGAGAGCAAGAAGGGAAGGGTCGGTGACCGGCGACGGCGTGCGGCGCTGCAGCCACTGTGCGACGGATAAGACCCCGCAGTGGCGCACGGGACCGTTGGGGCCGAAGACGCTCTGCAACGCGTGCGGCGTGCGGTTCAAGTCGGGTCGGCTCGTACCCGAATACCGACCCGCCGCGAGCCCTACGTTCGTGATGACTCAGCACTCGAACTCGCACCGCAAGGTTATGGAGCTCCGTCGCCAGAAGGAGCTGCTCCGCCACCAGCAGCAAGAACAATGCTACCGTCACACTCACCACGACTTCAAAGTCTGCTGA

>GmGATA10

ATGATGGATCTGAATGTGAATGAGAAAAAGAAATGTTGCGCTGATTGCAAAACCACCAAGACACCACTCTGGAGAGGAGGACCAGCTGGACCCAAGACCTTATGCAACGCTTGTGGAATTAGGTATAGGAAGAGAAGGGCTTGTTCGAGGAAGCGAGAGGAGCAGAGGTGGAAGATGTTGGGGGAAGAGGAACAGGCCGCGGTGTGCTTGATGGCCTTGTCCTCTGGTTTTGTTTTCGCTTGA

>GmGATA11

ATGGAGGTAGCAGTGGCCAAAGCGTTGAAACCGAGTTTAAGGAGAGAGTTCATCGTTCAGCAAATGCTCTGCGAGGATATTTTCAGCCTGAACGCCAACACCGTCGCCGCCGGCGAAGATTTCTCCGTCGACGACCTATTTGACTTCTCTAACGGCTCTCTTCACAACGAACAACAACAAGAATACGACGAAGGGAAACAAAGTTTATCGGCTTCAGAGGACCGCGGAGAAGACGATTGCAACTCCAATTCCACCGGCGTTTCCTATGATTCACTTTTCTCCACCGAATTAGCCGTTCCGGCCGGTGATTTGGAGGACCTAGAATGGGTTTCACACTTTGTGGACGATTCACTCCCGGAGCTGTCACTTCTATACCCGGTTCGTTCGGAGGAAGCAAACCGGTTTGTTGAACCGGAACCCTCAGCGAAGAAAACGCCTTGTTTTCCGTGGGAAATGAAAATAACGACCAAGGCGAGAACCGTACGGAATAGGAAGCCCAGCAACAGTCGCATGTGGTCGTTGGGTAGCCCGTTGCTCTCGCTGCCGTCTTCGCCGTCTTCGCCGTCGTCGTGTTCTTCTTCGGTGAGGGAGCCGCCGGCTAAGAAGCAGAAGAAGCAAGCCCAGGCCCAGCCCGTTGGGGCCCAGATTCAGCGGCGGTGCAGTCACTGCCATGTGCAGAAGACGCCGCAGTGGAGGACCGGCCCACTGGGCGCGAAGACCCTCTGCAACGCGTGCGGGGTTCGGTACAAGTCGGGTCGGTTATTTTCGGAGTATAGACCGGCGTGTAGCCCCACCTTCTGCAGCGATATTCACTCCAATAGTCACCGTAAAGTGTTAGAGATTCGGAAGAGGAAAGAGGTGGCTCAACCGGATACCGGCTTGGCCCAGGCCCAGACTCAAATGGTTCCCACCTGCTGA

>GmGATA12

ATGACTTCTGTTTCACTGAACCCCAACCCCCCATGCCCTACGATACAAGATCAAAGTCAACTCTTCATTTCTGCGAATAATCACGAATCAACTTCTCTCTCATGTTGCACCTTCTTTCACATACTCGATCAAAGCCAAACCAAAGATATCAGAGATTTAAGACATGGTCATCAACAGGATGGGAAGCTTGTATTTCATATTGGACCATCAAACAACAACAACCAAGTGTGCAATTCATCCTCCGTTAAACTTCAACCTAAGCCCGTTAAGGCAGATTCAAGCAGCGAGTGTGGCCACCATAACGTTTCCTTGTACAAAATAGAGGACGAAGAGAACAAAAGAGATCATGATTATGAAAAATGGATGTCTTCAACTGCGAGGTTAACGAGAAAAATGATGAGGCTACCTAGCACTAGCAGCGATCTGGCCACAAAGAAAGCATTGAATAATATTACAAGGGTTTGTGCGGATTGCAACACAACCAGTACCCCACTCTGGAGGAGTGGTCCTAATGGTCCTAAGTCACTTTGCAATGCCTGCGGCATTCGACAGAGGAAGGCAAGAAGGGCAATGGCAGAAGCTGTAAATGGTTTTGCTCCTTCCGTGAATTCATCTTCTACAAAGATCAGAGTGCATCACAAGGAAAAGAAGTCTCGTACAAACCATTTTGCACGGTTCAGGCTTAAGTGCAAGTTGGCAACTACTAGTACTGCTGAAGGAACATCTCAGCAGGAGAACGTGAAGATTGATTTGAACGATTTTGGTTTGAGTTTGAGGGATAGTTCGGCTTTGAAGCAGCAAGTGTTTCCAATAATGGATGAAGTAGCTCAGGCAGCGATGCTTCTCATGGATTTATCTTGCGGCTTTGTTTACTGTTAA

>GmGATA13

ATGGAAGCACAAGAGTTTTTCCAGAACACCTTCTGCCCCCAATTCCCCTCAGACAGCAACATCACTCCCTCCAACGCCAACCCCTCCGCCGCCACCACCGACCACTTCCTCGTAGAAGACTTCTTCGACTTCTCCAACGACGACGACGCCACCTTCGACTCCCTCCCCACCGACGTCGACTCCCCCACCGTTACCCCTGTCGACACCACCACCAACTCCAATTTCCCCGCCTCCGCAGACGCCCATTTCCCCGGTGACCTCTCCGTCCCGTATGATGATCTTGCGGAATTGGAATGGCTGTCGAAATTTGCGGACGAATCGTTTTCGAGCGAGGATTTGCAGAAGCTGCAGCTGATAACGGGTGTGAGAGCGCAAAACGATGCTGCATCGTCTGAAACGCGTGACCCAAACCCGGTTATGTTCAACCCGCAAGTGTCGGTTCGGGGCAAGGCTCGGAGCAAGCGGACACGTGGACCCCCATGCAACTGGACCTCTCGCCTCGTTGTGCTCTCCCCGAACACGAAGTCGTCGTCGTCTTCTCACTCCGGCGCGGAAGGCGGCAGCGAGGGGCGGAAGTGCCTGCATTGCGCAACGGACAAGACCCCACAGTGGCGGACTGGGCCCATGGGCCCAAAAACGCTCTGCAACGCGTGCGGCGTGAGGTACAAGTCTGGGCGGCTGGTGCCAGAGTACAGGCCCGCCGCGAGCCCAACGTTTGTTCTGACGAAGCACTCGAATTCGCACCGCAAGGTGCTGGAGCTGCGGCGGCAGAAGGAAATGGTGAAGGTCCAGCACCAGCAGCATCAGTTTCTCCAATTGCAGCATCAGCAGAACATGATGTTCGATGTCCCATCATCCAACGGTGAGGATTTCCTCATCCATCAGCACGTGGGCCCAAATTTCACGCACCTCATCTAG

>GmGATA14

ATGTTCGGCTCCATCAACCAGATCGTCTCCGCCGAAGACACCGACGGCCCCGTCTCCGACCACCACATCCATTACAGTTCCCACACCATCGAAGACGACGGCGCCGCCTCCGATCACCACATCCATTACAGTTCCCACACCATCGAAGAAGACGGCGCCGCCTCCGACCACCACATCCATTACAGTTCCCACACCATCGAAGACGATGGTGCCGCTGTCGAAGACGTTTCCGCCGTTCCGGGCCCTGAAATCTCTATCGACAATTCTAGCCAGCTCACGCTCTCGTTTCGCGGCCAAGTCTATGTCTTCGATGCTGTTACACCTGATAAGGTTCAAGCAGTGTTGTTGCTGCTGGGAGGAAATGAACTAACTTCGGGTTCGCAATGTGCGGAGCTATCATCTCGAAATCAGACTGGTGAGGAGGAATATCCTGCAAAATGTAGTCTACCACATCGAGCAGCGTCATTGAATAGGTTCCGTCAGAAGAGGAAAGAGCGGTGCTTTGATAAGAAAGTTAGATATAGTGTACGTCAAGAAGTTGCACTCAGGATGCATCGAAATAAGGGTCAATTTACTTCATCAAAGAATCAGGATGGAACTAATAGTTGGGGTTCAGATCAAGAGTCAGGGCAGGATGCTGTTCAATCAGAAACCTTGTGCTGCACACATTGTGGAATAAGTTCAAAATCCACTCCAATGATGCGTAAGGGGCCATCTGGTCCAAGGTCACTTTGCAATGCTTGTGGGCTTTTTTGGGCAAATAGGGGCACTTTGAGGGATCTTTCTAAGAGAAACCTGGAACACTCTCTTACACCACCTGAGCAGGTTGATGAGGGCAGTAATAATAATGCCTTGGACATTCGGAGTGGCATCCCTGCACAGCATAACAATCTTGTTAATGATAGCAAAGCTTTGGTATCTGATCGTTGA

>GmGATA15

ATGGCGACCGTGAATCCGCAGCCTCTGCAGTTCGAGGACCCAGCGATACCCGTCGACGATGACGATGACGACGACGACGACGGTGGTGATGATGATGCTATGGATGAGTTGGAGGATGCAAATGTTAACTCAGTGAACGTTACTAATGCCGCGAGTGTAAATCATGAAGCGGTAGTGGCTATGCCTTCCAGAACTAGCGAGCTCACTCTTTCTTTCGAGGGAGAAGTTTATGTCTTTCCCGCTGTCACTCCTCAGAAGGTGCAAGCTGTCTTGCTGCTTTTGGGAGGACGTGATGTGCAGGCAGGTGTGCCAGCAGTTGAACCACCATTTGATCAAAGTAACAGGGACATGGGTGATACTCCAAAGCGTTCAAACCTTTCACGAAGAATAGCATCCTTGGTCAGGTTCCGCGAGAAACGCAAGGAAAGATGTTTTGACAAGAAAATTAGGTATTCCGTCAGAAAAGAGGTAGCACAGAGGATGCATCGGAAGAATGGGCAGTTTGCTTCCTTGAAGGAAAGCCCAGGTTCATCTAATTGGGATTCTGCACAGAGTTCTGGTCAAGTTGGCACTTCTCATTCTGAATCTGTACGTAGATGTCACCATTGTGGTGTCGGTGAAAATAATACTCCTGCAATGCGTCGGGGGCCAGCTGGACCAAGGACTTTATGTAATGCATGCGGCCTTATGTGGGCTAACAAGGGCACACTCAGAGATCTCAGTAAGGGTGGAAGGAATCTTTCTGTTGAGCAAAGTGATCTGGACACTCCTATTGATGTCAAGCCTACTTCTGTTCTTGAAGGGGAATTGCCTGGCATCCATGATGAGCAAGGTAGTTCTGAAGATCCTTCCAAGTCCAATGCAGCGGATGGTTCTAGTAATCATGCTGTAAACCCCAGTGATGAGGAATTGCCTGAAACTGCAGAACACTTTACGAACGTTCTGCCACTGGGAATTGGTCATTCTTCAACAAATGACAGTGAACAGGAACCTCTGGTTGAGCTTTCTAATCCTTCAGATACAGATATTGACATTCCAGGAAACTTTGATTAG

>GmGATA16

ATGGGCAAGCAAGGGCCTTGCTATCACTGTGGAGTTACAAGCACTCCACTTTGGCGTAATGGGCCACCTGAGAAGCCAGTACTATGCAATGCATGTGGATCTCGATGGAGGACAAAGGGAACACTTGCAAATTATACCCCTTTGCATGCCCGGGCAGAAAATGTTGATTATGAGGATCAAAAGGTTTCCAGGGTAAAGAGCATTTCATTAAATAAGAACAAAGAAGTGAAATTAGCCAAACGAAAGCAAAACTATGATAATGCTGCATCTGGAGGGTTTGTTCCTGATTATAGTCAAGGATACCAAAAAGTTGTGGATGAAGATACAAGCAATAGATCAAGCTCAGGGTCAGCTGTCTCTAACTCAGAGAGCTGTGCTCAATTTGGTGGGACTGATGCTAGTGATTTGACAGGTCCTGCTCAGTCAGTGGTCTGGGATGCCATGGTGCCTTCCAAAAAGAGGACATGTGCAGGTCGTCCGAAGCCCTCATCTGTTGAGAAGCTCACAAGAGACCTGTGCACTATTCTTCATGAACAACAGTCTTATTTTTCTGCATCTTCTGAAGAGGATCTTCTTTTTGAAAGTGATACACCAATGGTCTCTGTTGAGATAGGACATGGAAGCATTCTCATCAGGCATCCTAGCTCTATAGCTCGTGATGAAGAGTCTGAGGCTAGCTCTCTCTCAGTTGATAATAAACAATGCCTAATGAATGAAGCATATTCATTTTCTAGTACCATTCCTATGTACAGTGATCGCAGTGGCATGAACTTCTCATCTCATGGAGTTGAAAAGATCAAAAACTCAGCAGGCCAAATCATGAAACAAGAGAAACTTGAAAGGGACAAGTCTCAGCTTGAAAAACTACAAGTTCCTGGAAATCATGATTCACCACTGTGCTCAATAGATTTAAATGATGTAGTCAACTATGAGGAGTTTATGAGAAACTTGACAAATGAAGAGCAGCAGCAATTACTGAAGTATCTCCCGGTGGTTGATACTGCTAAATTTCCTGATAGCCTTAGAAACATGTTCAATAGCTTCCAATTCAAGGAGAACTTAATCTATTTTCAGCAACTTCTTGGGGAAGGAGTCTTTGACATCTCTTTGTTGGGGGCAAAACCTGAAGAATGGAAGACATTAAAAAGGCTTGCGTTATCTAATCTGTCAAAGTCAAAATGGGTAGAACACTATAATTTCCTGAAGAAATGTGAAAACAAATCTGGAAAATCTATTGGTTTGGGATCTACTGCTATGGAATCTAGTTATGTTACAACTGCCAAGAGAATGCGTGAGCATGACAGCCAAAATCAAAATTTTCCAGAATTGAAGACAACAATGAGAAGCCCCAAAAGGGTGTTCATAAAGCCTAGCTGTGAGGTCAAAGAAGTTGTAGAAGAAGGCTCTAGCTTCAGTCCAAAAAGCCTATTTGCTTTACCCCATGGTGTTGGTAGCTTGCACATGTTGGATTCTTTCAACTTTGTTGGTGAGAGTTCTGAAGATCTGCTGTTAGAGGTGCCTTCTAACAGTTCTTTTCCACAGGCAGAGCTTCTGCACCCATCTTTAAGCTATGGTGCTCAGGTCAGCACCACTAGTAGCTCAGTACACTCTCTTGTTACTCATCCTTAA

>GmGATA17

ATGGCGAAAAGGAATGGTCCATGCTTCCACTGCGGCATTAAGTCTTCTCCACATTGGCGTAGTGGACCAGAAGACAAGTCAGTGTTGTGCAACGCATGTGGACTAAGATACACCAAATGGGGAAGCATTGGCCTTCAGAACTATTTTCCCAACCATTTTAAACCTGAGTATCTTGATAACCTTAAAAATCTAGAGGGTAGAAACAATGTTCTCCAAGGTTCAAGCTATGCTACAGATTCAAGTGGAAAAATCCATGTTATGTGGAATCCTTATGTTCCATCAAGGAAACGTTCACGCGTAGTACGGATGACAACATCAATCCAAAGGTTTCATGAACAACTTCTCATGATGTGGAAAAATGAAGAGAACTCAAATGATCAGTCATCCCAAGAATCAGAAGAGGTCTTATTGATTGATAACGTCAATAACTTCATACCTTGTAACGAAATAGGGCTTGGATGCATTCTTCTTAAACCAGAAGATGCTTCTGCATAG

>GmGATA18

ATGGGGAGAAAGCATGGTCCATGCTTCCATTGCAAAATTCATATAACTCCACTTTGGCGTAATGGACCTGAAGACAAGCCAGTATTGTGCAATGCATGTGGATCAAGATACAGGAAATGTGGAAGTCTTGAGAACTATCTTCCCAACCATTTTCAACCAGAGTACCCTGATAACCTTAAAATGCTGAAGCGTAGAAAAACTCTGAAGGGTGGAAAAGGTCGATATTTATGCAGCCCTAAGATTCCTACGAGAAAACGTTCACCATTGGTACGCAAGAAGATAACACCAATGAAGAGGTTTTATATGCAACTTCAAAATATGTGGGAAGATTACGGGAACTCGAATGAGTCCTCCTCAGAAGAGGTCTTAATTTTCAATAACGTCAATAACTTCATACCTAGTAATGAAATAGGCCTTGGATGTATTCCTCTTAAACTAGATGATGCTTCTGCATAG

>GmGATA19

ATGGAGGTAGCAGTGGCCAAAGCGTTGAAACCGAGTTTAAGGAGTGAGTTCATCGTTCAGAAAATGCACTGCGAGGATATTTTCAGCCTGAACGCGAACACCGTCGCCGTCGGCGAAGATTTCTCCGTGGACGACCTATTTGACTTCTCTAACGGCTCTCTGCACAACGAACACCAACAAGAGTGCGACGAAGAGAAACAAAGTTTATCTGCTTCGTCGCAGTCACAGGACCGTGGAGAAGACGATAGCAACTCCAATTCCACCGGCGTTTCCTATGATTCACTTTTCTCCACCGAATTAGCCGTTCCGGCCGGTGACTTGGAGGACCTAGAGTGGGTTTCACACTTCGTGGACGATTCACTCCCGGAGCTGTCACTTTTATACCCGGTTCGTTCGGAGGAAGCGAACCGGTTTGTTGAACCGGAACCCTCAGTGAAGAAAACGCCTCGTTTTCCGTGGGAAATGAAAATAACGAGCAAGGCGAGAAGCGTACGGAATAGGAAGCCCAACACTCGCGTGTGGTCATTGGGCAGCACGTTGCTCTCGCTCCCGTCTTCGCCGCCCGCGAAGAAGCAGAAGAAGCGGGCTGAGGCCCAGGTCCAGCCCGTTGGAGTCCAGATTCAGCGGCGGTGCAGTCACTGCCAGGTGCAGAAGACGCCGCAGTGGAGGACCGGCCCACTGGGCGCCAAAACCCTTTGCAACGCGTGCGGGGTTCGGTACAAGTCGGGTCGGTTATTTTCGGAGTATAGACCGGCGTGTAGTCCCACCTTCTGCAGCGATATTCACTCCAATAGTCACCGTAAAGTGTTAGAGATTCGGAAGAGGAAAGAGGTGGCTGAACCGGATACCGGCTTGGCCCAGACTCAAATGGTTCCCACCTGCTGA

>GmGATA20

ATGGAAGCACAAGAGTTTTTCCAGAACACCTTCTGCCCCCAATTCCCCTCCGGTACCAACATCACACCCTCCAACGCCAATCCCTCCGCCGCCACCGCCGACCACTTCCTCGTCGAAGACTTCTTCGACTTCTCCAACGACGACAACGACGCCACCGCTGTCACCGACGCCACCTTCGACTCCCTCCCCACCGACGTCGACTCCCCCAACGTTACCCCCCTCGACAGCACCACCAAGAACTCCAATTTGCCCTCCTCCTCCTCCGCCGACGCCCATTTCTCCGGTGACCTCTCCGTCCCGTATGATGATCTTGCGGAATTGGAGTGGCTGTCGAAATTCGCGGAGGAATCGTTTTCGAGCGAGGATTTGCAGAAGCTGCAGCTGATATCGGGTGTGAGAGCGCAAAACGATGCTGCATCGTCTGAAACGCGTGACCCGAACCCGGTTATGTTCAACCCGCAAGTGTCGGTTCGAGGCAAGGCTAGGAGCAAGCGGACACGTGGGCCCCCATGCAACTGGACCTCTCGCCTCGTTGTCCTCTCTCCCAACACCACGTCGTCGTCTTCTAACTCCGACGCCGGTAAGAAGCCGGCGACGCCGCGGAGGAGAGAGGCTGCCTTCGCCGAAGGCGGCAGCGAGGGGCGGAAGTGCCTGCATTGCGCCACGGACAAGACCCCGCAGTGGCGGACTGGGCCCATGGGCCCAAAAACGCTCTGCAATGCGTGCGGCGTGAGGTACAAGTCGGGCCGGCTCGTCCCAGAGTACAGGCCCGCGGCGAGCCCAACGTTTGTTCTGACGAAGCACTCGAATTCGCACCGCAAGGTGCTGGAACTGCGGCGGCAAAAGGAAATGGTGAAGGTCCAGCAGCATCAGTTTCTCCAACTGCATCAACAGAACATGATGTTCGATGTGCCATCATCCAACGGTGAGGATTACTTGATCCATCAGCACGTGGGCCCCGATTACACGCACCTCATCTAG

>GmGATA21

ATGTTCGGCTCCATGAACAAGATCGTCTCCGCTGAAGACACCGACGGCTCCGTCTCCGACCACCACATCCATTACAGTTCCCACACCATCGAAGACGACGGCGCCGCCTCCGATCACCACATCCATTACAGTTCCCACACCATCGAAGAGGACGGCGCCGTCTCCAACCACCACATCCATTACAGTTCCCACACCATCGAAGAAGACGGCGGCGCCACTGTCGAAGAGGTTTCCGCCGTTCCGCCCCTTGAAATCTCTATCAACGATTCTAGCCAGCTCACGATCTCGTTTCGCGGCCAAGTCTATGTCTTCGACGCTGTTACACCTGATAAGGTTCAAGCAGTGTTGTTGCTGTTGGGAGGAAATGAACTAACTTCGGGTTCGCAATGTGCGGAGCTATCATCTCAAAATCAGACTGGTGAGGAGGAATATCCTGCAAAATGTAGTCTACCACAGCGAGCAGCGTCATTGAATAGGTTTCGTCAGAAGAGGAAAGAGCGGTGCTTTGATAAGAAAGTTAGATATAGTGTACGTCAAGAAGTTGCACTCAGGATGCATCGTAATAAGGGTCAATTTACTTCATCAAAGAATCAGGATGGAACTAATAGTTGGGGTTCAGATCAAGAGTCAGGGCAGGATGCTGTTCAATCTGAAACCTTGTGCACACATTGTGGAATAAGTTCAAAATCCACTCCAATGATGCGTAGGGGGCCATCTGGTCCAAGGTCACTTTGCAATGCTTGTGGGCTTTTTTGGGCAAATAGGGGCACTTTGAGGGATCTTTCTAAGAGAAACCAGGAACACTCTCTTGCACCACCTGAGCAGGTTGATGAGGGCAGTAATAATAATGACTTTGACTGTCGGAGTGGCATCCCTGCACAACATAACAATCTTGTTAATGATAACAAAGCTTAA

>GmGATA22

ATGGCGACCGTGAATCCGCAGCCTCTGCAGTTCGAGGACCCTGCTATACCCGTCGACGACGATGACGACGACGACGACGGCGGTGATGATGACGCTATGGATGACTTGGAGGATGCGAATGTTAATTCAGTGAACGTTGCTGCTAATGCCGCGGCGAGTGTAAATCATGAAGCGGTAGTGGCTATGCCTTCCAGAACTAGCGAGCTCACTCTTTCTTTCGAGGGCGAGGTTTATGTCTTTCCCGCTATCACTCCTCAGAAGGTGCAAGCTGTCTTGCTGCTTTTGGGAGGACGTGATGTGCAGGCAAGGGTGCCTGCTGTTGAACAACCATTTGACCAAAGTAACAGGGGCATGGGTGATACTCCAAAGCGTTCAAACCTTTCACGAAGAATAGCATCCTTAGTCAGGTTCCGTGAGAAACGCAAGGAAAGATGTTTTGACAAGAAAATTAGGTATTCCGTCAGAAAAGAGGTGGCACAGAGGATGCATCGGAAGAATGGGCAGTTTGCTTCCTTGAAGGAAAGCCCAGGTTCATCTAATTGGGATTCTGCACAGAGTTCTGGTCAAGATGGCACTTCTCATTCTGAATCTGTACGTAGATGTCACCATTGTGGTGTCAGTGAAAATAATACTCCTGCAATGCGTCGGGGGCCAGCTGGACCAAGGACTTTATGTAATGCATGCGGCCTTATGTGGGCTAACAAGGGCACACTTAGAGATCTCAGTAAGGGCGGAAGGAATCTTTCTGTTGAGCAAAGTGATCTGGACACTCCTATTGATGTCAAGCCTACTTCTGTTCTTGAAGGGGAATTGCCAGGCATCCATGATGAGCAAGATAGTTCTGAAGATCCTTCCAAGTCCAATGCAGCGGATGGTTCTAGTAATCATGCTGTAAACCCCAGTGATGAGGAATTGCCTGAAACTGCAGAACACTTTACGAACGTTCTGCCACTGGGAATTGGGCATTCTTCAACAAATGAGAATGAACAGGAACCTCTGGTCGAGCTTTCTAATCCTTCAGATACAGATATTGATATTCCAGGAAACTTTGATTAG

>GmGATA23

ATGCGTGAGAAATTTCACTTGTGCCACTCCTTTACTCCTCAAAACCCCACCATCAGTGTCTCACACAGCAAACCAAAGCAAAGGAATCCTCATTCCTTCCATCAAAACGGTGCGCTTCGCTCCCTCTCTCTAAAACCAACACATACTACTTCTCTTCAGCTTCTCCCACTAAAACACACTATCTATATGGAAGTGCCGGAATACTTCGTCGGAAGCTTTTTTGGCACCGGAGGAGCCGAACAGTTCTGCCCTCCGGAGAAGCGCCACTCCGACCAGAAAACCGGCGAGCCTTTCGCCATTGACGACCTCCTCGACTTCTCCCACGCCGACGCCATTATGTCCGACGGTTTCTTCGACAATGTCGCCGGAAACTCCACCGACTCCTCCACCGTCACCGCCGTCGACAGCTGCAACTCCTCAATTTCGGGCAGCGACAACCGCTTCGCCACCACCATTGTCCCTCGCGGCTTCCCCAGTGATCCTCAATTCTCCGGAGAACTCTGCGTTCCGTATGATGAAATGGCGGAACTGGAATGGCTCTCTAACTTCGTGGAAGACTCGTTCTCCGCCGAGGAGGAGCTGAAGACGCTGCAGCTACTCTCCGGCGCCGCCGCCGCGTCCACCGCCATTGGCGCGAAACCGCAGACGCCGGAGTCCTCCTCCTCCACCGACACGCTTCCGCCGTTCGCCTCCGACGACACCTTGCGAAACGCACCGTTTCTCCACTCGGAAACGCCTCTCCCGGGGAAGGCGCGCAGCAAGCGCTCACGGGCGGCGCCGGGGGACTGGTCCACGCGCCTGCTCCACCTGGTCGCGACGGAGCAGGAGAAGCTGCCGCAGCTAAAGGCGGAGCCGGCGAAGAAGAGAGAAGGAACGAATGCGGAGTGTTCCGGACGCAAATGCCTGCACTGCGGTACGGAGAAGACGCCGCAGTGGAGAACGGGACCGATGGGACCGAAGACGCTGTGCAACGCGTGCGGCGTAAGGTTCAAGTCCGGGAGGCTGGTGCCGGAGTACCGACCGGCGGCGAGTCCAACGTTCATGTCAACGAAGCATTCGAATTCGCATCGGAAGGTTTTGGAGCTGAGGCGGCAGAAGGAGCTGCAGCGACAGCAGCACCAGCAATTGATGAGTCAAAGTTCAATTTTCGGCGTATCCAACGGTGGGGATGAGTTCTTGATCCATCATCATCACCAGCATTGTGGGCCAGACTTTAGACACGTTATTTAG

>GmGATA24

ATGGACTTGTACGGTTCCTTTTCCACCCCTTCAGATTGCTTACATATTGATGATTTTCTAGATTTCTCCAACATCACCACCGACACCCACCACCACCTTCCTCCTCCGCAAAACTCTCCATTAATCTCCCACGACGACGCCAATCTCTTCTTCAATTTCCCTTCCGTTCCGACCGACGAAGCGGCGGAGCTGGAGTGGCTCTCCCAGTTCGTGGACGACGACGCGACGTCGTTTCACAGCTTCCCAGCAACCGCATCGATTGGATCCCACTCGACGTCGTTTCTCTCCAACAATAACAACAGAAACGATAATAACGAATATCCCAAATCTTCATTATCTTCAAACATTCCATGTTCTTCAGCGGTGGCGGGAAAATCGAGAGCGAGGAGGGAAGGGTCGGTTACCGGCGACGGCGGCGTGCGGCGGTGCAGCCACTGTGCCTCGGAGAAGACGCCGCAGTGGCGCGCGGGACCGCTGGGACCGAAGACGCTGTGCAACGCGTGCGGGGTTCGGTTCAAGTCGGGTCGGCTCGTACCCGAATACCGACCCGCGGCGAGCCCTACGTTCGTGCTGACTCAGCATTCGAACTCGCACCGCAAGGTCATGGAGCTCCGTCGCCAGAAGGAGCTGCTCCGCCACCAACAACAACAACAATTACAACAAGAGCAATGCCACCGTCACACTCACAACCACCACGATTTCAAAGTTTGCTGA

>GmGATA25

ATGGATAACAGCCTGAACAATCCGAGCGACAACGGGGAAGACCCGCCACCGCCGCCGCCGGTTCCCATGCAAGTCGACGGCTTCCAACCGTTCCATTACGCCAATTGTTCCGATGAAGGGGAAGAAGCCGTTCCGGTCACCAATGCTTCTTCTGCTATGCACGCCAGAGCCAGCGAACTCACCATTTCCTTCGAAGGCGAAGTCTATGTTTTCCCAGCAGTTACGCCGGAAAAGGTGCAGGCCGTATTATTACTCTTGGGAGCGCAGGAAATGACAAATAGTGCCCCCACCTCTGACATTTTGCTGCAGCAAAACTATCAGGACATTAGGGAAATAAATGATCCTTCCCGAAGTTCAAAGCTTTCACGAAGATTTGCATCACTTGTTAGGTTTCGTGAAAAACGAAAAGAGAGATGTTTTGAGAAGAAAATCCGGTATTCTTGCCGTAAAGAGGTTGCCCAGAGGATGCATCGTAAGAATGGACAGTTTGCATCAATGAAGGAAGACTACAAATCTCCTGCTGAAAATTGGGATTCAAGCAATGGTACTCCTTGCCCAGAATCTACTGAACGTAGATGCCAGCATTGTGGAATTAGTGAGAAGTCTACTCCAGCTATGCGTCGAGGACCAGCAGGTCCAAGATCTCTGTGCAATGCCTGTGGGCTCATGTGGGCAAATAAGGGAACTCTGAGAGATCTCAGCAAAGCAGCAAGGATTGCTTTTGAACAAAATGAACTGGATACTTCAGCTGATATAAAGCCTTCAACAACAGAAGCCGAACATTCTTTTGCTAAGCAGGACAAGGAGGGAAGCCCTGAAGAAACTAAGCCTGTGCAAATGGATTCCAGTCGGTCACCTGAGAAGACAAATGACCAGTTTATTATCGGAACTGCTGAATCAGTTACTGACAACTTGTCCATCCAAGTGGAGAATCATGCTCTTAGTCTACATGAGCAGGATACTCTTGAGGATCTTGCTGATGCTTCCGGGACCGAATTTGAGATTCCTGCAGGTTTTGATGATCAGGTTGACATTGATGATTCCAACATGAGGACTTACTGGCTGTGA

>GmGATA26

ATGGTGGATCCAACTGGAAAAGGATCGGAGATTGAAGTCGAGGACTCAAACTCAAACCCTAACGCTCCTTCCTCAGGGAACAGTCCAAGCAGCAACAATGAGCAGAAGAAAACCTGCGCCGATTGCGGCACCACCAAGACTCCTCTCTGGAGGGGTGGTCCTGCAGGACCCAAGTCTCTGTGCAACGCGTGCGGGATCAGAAGCAGGAAGAAGAAGAGAGCGATCTTGGGAATAAACAAGGGGAGCAACGAGGACGGAAGGAAAGGAAAGAGGACCGGCGGCGCTTTGGGGAAGGAGGTGTTGTTGCACCGATCGCATTGGAAGAAGCTCGGAGAGGAAGAGAAAGCTGCGGTCTTGTTGATGTCGCTCTCTTATGGATCCGTTTATGCCTGA

>GmGATA27

ATGGATAACAGCACCCTGAACAATCCCGGCGACAACGCCGAAGACCCGCCACCGCCGCCGGCTCCCATGCAAGTCTTTGATTCCTTCCAACCGTTCCATTACGCCAACGGTTCCGATGAAGGAGAAGAAGCTGTTCCTGCTCCAGTCGCCAATGCTTCTTCTGCTATGCGCGCCAGAGCTAGCGAACTCACCATTTCCTTCGAAGGCGAAGTCTATGTTTTCCCAGCCGTTACGCCCGAAAAGGTGCAGGCTGTATTATTACTCTTGGGAGCACAGGAGATGCCAAATAGTGCCCCCACCTCTGACTTTTTGTTGCAGCAAAACTATCAGGACATTCGGGAAATAAATGATCCTTCCCGAAGTTCAAAGCTTTCAAGAAGATTTGCATCACTTGTTAGGTTTCGTGAAAAACGAAAGGAGAGATGTTTTGAGAAGAAAATTCGGTACTCTTGCCGTAAAGAGGTTGCTCAGAGGATGCATCGTAAGAATGGACAGTTTGCATCATTGAAGGAAGACTACAAATCTCCTGCTGAAAATTGGGATTCAAGCAATGGTACTCCTTGCCCAGACTCTACTGAGCGTAGATGCCAGCATTGTGGAATTAGTGAGAAGTCTACTCCAGCTATGCGTCGAGGACCAGCAGGTCCAAGATCTCTGTGCAATGCCTGTGGGCTCATGTGGGCAAATAAGGGAACTCTGAGAGATCTCAGCAAAGCAGGAAGGATTGCTTTTGAACAAAATGAACTGGATACTTCAGCTGATATAAAGCCTTCAACAACAGAAGCCAAACATTCTTATGCTAAGCAGGGCAAGGAGGGAAGCCCTGAAGAAACTAAGCCTGTGCAAATGGATTCCAGACGGTCACCTGAGAAGACAAATGAGCAGTTTATTATTGGAACTGCTGAATCAGTTACTAACAACTTGTCCGTCCGATTGGAGAATCATGCTCTTATTCTACATGAGCAGGATACTCTTGAGGATCTTGCTGATGCTTCTGGGACCGAATTTGAGATTCCTGCAGGTTTTGATGATCAGGTTGACATTGATGATGCCAATATGAGGACTTATTGGCTGTGA

>GmGATA28

ATGTGGTATGTTTCACAGCCAAACCACCAGCTCCTTCGCCACGTTTTTCTCCATGCCCAACCACAAACCACCACCCTACATGATTCCGACAACATTTATGACTATTCTTCCTTCACTCCCTCTTCCTTTTCTTCCGTTGACTGCAACCTCTCCCTCGGTACCCCTTCCACCTGCGTCTCCGAAGACGAAGAAAAACGAAGCCGCCACGAATGCCATTCCGTCTCTAACTTCTGCTGGGACTTACTACAATCCAAACACAACAACCCTCAATCCCATTCCAAGTCCTCCGGAACCACCAACACCACCGACCCTCTCCTCGCTCGCCGCTGCGCCAATTGTGATACCACTTCTACTCCTCTCTGGAGAAATGCCCCCGTGGCCCTAAGGTACGTAAATAACTACAATATTTAG

>GmGATA29

ATGAAGAAGAAAGGTCCATGTTCGCATTGCAGAATTAGTTATACTCCACTTTGGCCTAATGGACCAGCTGATAAACCGGTGTTGTGTAACGCTTGTGGATCAAGATACAAGACAAGAGGACACCTTGACAATTATCTTCCCAAGAATGTCCATCCTCAGCCACACCACAAGAAATTTAAAAATGTAAATAGCGGAGGAAGTAATCTCAATGTTGAGCCTGAGCTTGAGTCCGGCAACCAACTTTTAAACCATGTTTCGCCTAGATCTACAACTAATGGTGACAGCGACAAGTTAACCTTAGATGTCCATCATATATCACCACAAGATTTTGGGAAGAAGATCCCATCAAAGAAACGGTCACCGATGGTGTACAAGCGTATGATACCAATGGAGAAGTTTCAAAAGCAGCTTGTTAAGTTGTATAAAAGTGAAAGACAACCAGAAGAGAGTGTTTTGGTGGATAACATGATGAACTTCATACCCGAAAATGAGATAGGACTTGGAACCATTCTTCTTAAGACAAATGATGATGATGCTTCTTCTACAGATAAATGTGGATCATCCACATCTGCACCCTGA

>GmGATA30

ATGGAGGCGCCGGAATACTTCGTCGGAGGCTATTTTGGCGCCGGAGGAGCCGAACAGTTCTCTCTGTCGGAAAAGCGCCACTCCGACCAGAAAACTGGCGAGCCTTTCGCCATTGACGACCTCCTCGACTTCTCCCATGCGGATGCCATTATGTCCGACGGTTTCTTCGACAATGTCACCGGAAACTCCACTGACTCCTCCACTGTCACCGCCGTCGACAGCTGCAACTCCTCAATTTCCGGCAGCGACAACCACTTCGCCACCGCCATTGTCCCCCGCTGCTACCACAGCGATCCTCAATTCTCCGGAGAACTCTGCGTTCCGTATGATGAAATGGCGGAACTGGAATGGCTCTCGAACTTCGTGGAAGACTCGTTCTCCGCCGAGGAGGAGCTGAAGACGCTGCAGCTGCTCTCGGGCGGCGGCGCCGCGTCCACCGCCATTGGCGCGAAACCGCAGACGCCGGAGTCCTCCTCCTCCACCGACACGCTTCCGCCGTTCGCCTCCCGACGGACCTTGCGAAACGCACCGTTTCTCCACTCGGAAACTCCTCGCCCAGGGAAGGCACGCAGCAAGCGCTCACGCGCGGCGCCGGGGGACTGGTCCACGCGCCTGCTCCACCTGGTCGCGCCGGAGAAGGAGAAGCCGCCGCAGGCGAAGAAGAGAGAGGGAACGAATGTGGAGTGTTCCGGACGCAAATGCCTTCACTGCGGCGCGGAGAAGACGCCGCAGTGGCGAACGGGACCGATGGGACCGAAAACTCTATGCAACGCGTGCGGCGTGAGGTTCAAGTCCGGGAGGCTGGTGCCGGAATACCGGCCGGCGGCGAGTCCAACGTTCATGTCAACGAAGCATTCGAATTCGCATCGGAAGGTTTTGGAGCTCAGGCGGCAGAAGGAGATGCAACGACAGCAGCATCATCAGCAGTTGATGAGTCAAAGTTCAATTTTCGGCGTATCCAACGGTGGAGATGAATTCTCGATCCATCATCATCATCATAATCATCACCTCCATTGTGGGCCAGACTTTAGACACGTTATTTAG

>GmGATA31

ATGGATGGTATTCATGGGGGTGATTCTCGGATACACATAACTGATGGACAGCATCCTATACACGTGCCATATGTGCAAGAACACGAGCATCATGGACTCCACCATATAAGCAATGGGAATGGGATAGATGATGATCATAATGATGGTGGTGATACTAACTGTGGTGGAAGTGAGAGTATGGAAGGTGAAGTCCCCTCCAACCATGGAAATCTCCCTGACAATCATGCTGTAATGATGGATCAAGGGGGTGATTCTGGGGATCAGCTTACGTTGTCTTTTCAGGGCCAGGTTTATGTCTTTGACTCCGTGTCACCAGAAAAGGTTCAAGCTGTACTACTGTTACTGGGAGGCCGCGAAATACCTCCAACTATGCCTGCCATGCCAGTATCTCCTAACCATAATAACCGGGGATATACTGGTACTCCGCAAAAATTCAGTGTCCCTCAGAGATTAGCTTCATTGATTAGGTTTCGTGAAAAGCGGAAGGAACGAAATTATGACAAAAAAATTCGCTATACTGTTCGTAAAGAAGTAGCATTAAGGATGCAAAGAAATAAAGGTCAGTTTACATCTTCCAAGTCCAATAATGATGAATCTGCATCAAATGCCACAAATTGGGGGATGGATGAAAACTGGACAGCAGACAATAGTGGATCCCAGCAGCAAGATATTGTTTGTAGGCACTGTGGCATCAGTGAGAAGAGTACACCAATGATGCGACGCGGGCCTGAAGGGCCAAGGACCCTCTGCAATGCTTGTGGTCTTATGTGGGCAAATAAGGGAATCCTGAGGGACCTATCAAGGGCGGCACCCTTATCTGGGACTATCAAGAATGAGAATAAAAGTTTAGAGGCCAACCAGATAGTCCATAGAGTTGCAGGAGAAGCTGATGATTCATCATGA

>GmGATA32

ATGGCTTTAGTAGGTGAACAAAAGCATTCACTCATGGTGAAGAAAGGTCCATGTTCGCATTGTGGAGTTACTCATACTCCACTTTGGCATGATGGACCAACTGAGAAACCAGTGTTGTGTAGCGATTGTGGATCACAATACAAGTTAAAAGGAAATTTAGACAATTATTTTCCCAAGAATCCCGTTGTTCAGTCATTCCACAATAAGTTTACAAATGTAAATGGTGGAAAACATCTCAATGTTGATGTGGACCAACTTTCAAACTATGTTCCACCAACTGATGAAGACAACAATATGTCAACCCCAAATGTCCATTGCATATCCGCGCAAGCTACTCCACTTTGGCGTAACGGACTAGTTGATAAATCGGTGTTGCACAAAGCTTGTGAATTATCCCCCCAAGAAACTCCAGCTTCAGCCATTAATGCCCCCAAGAAACTCCAGCTTCAGCCATTAATCCACAAGAACTTTATAAATGTAAATGGTGGAAGCAGTCTCAATGTTGAGGATGAGGACCAACTTTCAAACCATATTACACCTGCAAGTGATGGAGACAACAATAAATCAACCCCAAATGTCCAACACATATCACCACAAGATTTTGGGAGCAAAATCCCTTCAAGGAAACGGTCACGGGTGGTATACCTGACACCATTGAAGGAGTGTATGGAGGAGCTTTGGAAATTACATAGAAATTATGGAAGGCATGCAGAAGAGCGTATATTGGAGGATAATGTGAACAACTTCATACCCGAAAATGAGATAGCAGGACTTGGAGCCATTCTTCTTAAGACGGATCATGATGTTGCAGCTTCTGCAGATACATGTGAATCATCCACAGATGATTGA

>GmGATA33

ATGATACCAACTTATCGTTACTCAGTGTCTTCGCCTATGCCTATAGATCTTAATGAAGATCACACCCACCACGTCTTCAGTACAAGCCATCAAGCCTCTTCTTCGTCTTCTTCTCTATCTTTTTCTATTCTCTTCAACCCGGATCATCAAGGTCAAGGAGGATCTTGTTGCCACTGGGAATCAAAGCACTTACAAAGTGATGAAGAGGCCCAGAAGATTGTTCCTTCTAGTGAATCATGGGAGCATCCAGTATCTGAAAAGGATGAAAACAGAAGTGATCTCAAGCTGAGAGTTTGGAAGAAAGAAGATAAGTGTGAAAATTTTCAAGTTGAAGATAATTCAACTAAGTGGATGCCTTTAAAGATGAGAATGATGCGGAGGATGATGGTGTCAGATCAAACGGGTTTTGATACAGAAGGCATGATCTCTAACTCTAAGCAGATCAAGAATGAAGAGAAAAACCCACCACTGACACCTCTAGGAACTGATGATAGTAATAACTACAATTCTTCTGCAAACCACAGCAAGATCACTGTTAGGGTTTGTTCTGATTGCCACACCACTAAGACCCCTCTCTGGAGAAGTGGACCAAAAGGTCCAAAGACACTTTGCAATGCTTGTGGAATTCGACAAAGGAAGGCAAGGCGTGCCATTGCCGTGGCTGCAACGGCAAACGGAATGAATCCAGTGGAGGCTGAGAAATCTCAAGTAAAGAAAGGAAACAAATTGCATAGCAAAGGGATGAAGTCCAAAACTAAGGGTGCACCACATATGAAAAAGAAGCGCAAACTTGGAGCGAAGTATAGAAAGAGGTTTGGTGCTTTTGAGGATTTGACAGTGAGATTGAGTAAGAACTTGGCTCTCCAAAAAGTCTTCCCTCCGGATGAGAAGGAGGCTGCAATCTTGCTCATGGCTTTATCTTTATGGCCTTCTTCATGGCTTTCCCACAGATCGTTACGTCAATTACTTAGATTTATGAGATATATTGGTGGTAAAACCATAATTATTGTATGA

>GmGATA34

ATGGAAACCATTGGTTCTGTGGATGACCTCTTGGATTTTTCATCAGACATAGGCGAGGAAGATGATTACGATGACAAACCTAGGAAAGCCTGTCCTTCACTTAACTCAAAATGCGCCGGCCCATCGTTGTTTAACCCATTGGTCCAGGTTGATCCAAACCATTCATTTTCTGAGTTTGCAGAAGAGGAGCTTGAATGGCTATCCAACAAAGATGCATTTCCTTCTGTTGAAACGTTTGTCGACCTATCATCTATTCAACCCGGCACAACCAAGAACCAAAAATCAGCCCCAGTACTTGAGTGTAGCACAGGCAGTAGCAACAGTAATAACAGCACTAACAGTATTTCCCTCCTAAACAGCTGTGATCACCTGAAGGTCCCAGTCCGTGCACGGAGCAAGAGTCGCAGTAGGCATCGCCCTGGCCTTGCTGAGAATTCTAGTCAGCAAGTCTGGTGGAGACAGCCAAGTAATGGAACTTCCAAAGCAGATGAAGGGATGAAAATCTCATCCATCGGTAGAAAATGTCAGCACTGTGGAGCTGAAAAAACTCCACAATGGCGGGCAGGTCCCTCTGGTCCAAAAACACTTTGTAATGCATGTGGGGTTAGGTTCAAGTCTGGGCGGCTTGTGCCTGAATACCGTCCTGCAAGTAGCCCAACTTTTCATAGTGATTTGCATTCTAATTCCCACAGGAAGATAGTAGAGATGAGGAGGCAGAAGCAAATGGGAATGGGATAA

>GmGATA35

ATGGTTGGACCTAACTTCATGGATGAGATAGACTGCGGCAGCTTCTTTGACCACATCGACGACCTTCTCGATTTTCCCGTCGAGGACGTCGACGGCGGCGCTGCCACCTTGCCTTCCGTCTCCGCCGGAAACAGCAACTCGCTGGCGAGCATCTGGCCCTCCGAGTCCGACTCGTTTCCCGCCTCCGACTCGGTGTTTTCCGGCAACAGTGCTTCGGACCTCTCGGCCGAGCTATCCGTTCCGTATGAAGACATTGTCCAATTGGAATGGTTGTCCAACTTTGTGGAGGATTCCTTTTGTGGGGGGAGCCTAACAATGAACAAAGTGGAAGAGCCATCATGTACCACTAAGGAGGACTCGGTCAACACCCAATTTCACACATCAAGCCCAGTTTCTGTCCTCGAAAGTAGCAGTTCTTGCTCTGGTGGCAAGACTTTGCCACCTCGCAGTCCAGAGATTTACATCCCTGTGCCGTGTGGACGTGCACGCAGCAAGCGTCCACGTCCAGCAACCTTCAATCCTAGGCCTGCCATGAACCTTATTTCCCCTGCCTCCTCTTTTGTTGGGGAGAATATGCAGCCTAATGTCATATCATCCAAGGCCTCTTCAGATTCTGAGAATTTTGCTGAGTCTCAACTTGTTCCCAAGATGCCGAAGCTAGCTTCTGGGGAGCCTAAGAAGAAAAAGAAAGTGAAGGTGCCACTTCCAGTAGCTCCAGCTGATAACAATCAAAATGCCTCACAACCTGTTAGGAAATGCATGCATTGTGAGATAACCAAGACACCACAGTGGAGGGCAGGGCCAATGGGGCCAAAAACACTATGCAATGCATGTGGTGTTCGTTACAAGTCCGGCCGGCTCTTCCCCGAATACCGGCCTGCTGCAAGTCCAACTTTTTGCCCATCCGTGCACTCCAATTCTCATAAGAAGGTCCTGGAAATGAGATGCAGGGGATTTGACAAATCTGGTTTTGCAATCAATTCAGCTGCCTCACCTGAACTCATTCCAAACACTAACAGCAGCCTTACCCTGGAGTACATGTGA

>GmGATA36

ATGGGCAAGCAAGGACCTTGCTATCACTGTGGAGTTACAAGCACGCCACTCTGGCGCAATGGACCACCCGAGAAGCCAGTACTATGCAATGCATGTGGGTCTCGATGGAGAACAAAGGGAACTCTTGCAAATTATACCCCTTTACATGCTCGAGCAGAAACTGATGATTATGATGATCAAAGGGTTTCCAGGATAAAGAGCATATCGATAAATAAGAAGAAAGAAGTGGCATTGCTCAAACGAAAACAGAACCATGATAATGTAATGTCTGGAGGGTTTGCACCTGATTACAACCAGGGATACCAAAAGGTTGTAGATGAAGATATTAGCAACCGGTCGAGTTCAGGATCAGCTATCTCTAACTCAGAGAGCTGTGCACAATTTGGTTATGGTGGCATGGATGCTAGTGATCTGACAGGTCCTGCTCAGTCAGTGGTCTGGGATGCCATGGTGCCTTCAAAAAAGAGGACATGTGTTGGTCGTTCAAAGCCTTCTTCTGTTGAGAAGCTAACAAAAGATTTATGTACTATTCTTCATGAACAACAGTCATATTTTTCGGCATCTTCTGAAGAAGATCTTCTTTTTGAAAGTGATACACCAATGGTCTCTGTTGAGATAGGACATGGAAGCGTTCTCATTAGGCATCCTAGCTATATAGCTCGTGAAGAAGAGTCTGAGGCTAGCTCTCTTTCAGTTGATAATAAACAATGCCCAATGAGTGAGGCATATTCTTGTTCTGGTGGCATTCTAATGCATAATGATTCCAGTCGCTTGAAGTCTTCATCTCTGGAAGTTGAAAAGATCGGGAACTCTACTGGTCAAGGAGTGCTGCAGGAACAACTTAAAAGTGACAAGTCTCAACATGAAAGAGTACAAATTCTTGGCAATCATGAATCCCCATTGTGCTCAATAGATTTAAATGATGTTGTAAACTATGAAGAGTTTTTGAGAATCTTGACAAATGAAGAGCAACAGCAATTACTGAAGTTACTTCCTGTGGTTGATACTGCTAAACTCCCTGATAGCCTTAAAGTCATGTTCAATAGCTCTCAATTCAAGGAGAACTTAACTTACTTTCAGCAGCTTCTTTCAGAAGGAGTCTTTGATATCTCTTTGTTGGGGGCAAAACCTGAAGACTGCAAGACTTTGAAAATACTTGCATTATCCAATCTGTCAAAGTCAAAATGGGTAGAACACCATAATTTTCTCAAGAAATATAAAAACAAAGCTGTAAAATCTAATACTATGGGATCTACTGGTACAGCATCAATTAATGTTTTGAACAACAGGGCATCAACTAATGTTGCAAATATCAAGAGAATGTGTGATAGCAGAAATCAAAACTTTCCAGAATTGAAGACAATAATGAGGAGCCCCAAAAGAATGATCACAAAGGCTAGTTTTGAGTGCAAAGAAGCTGTAGAAGATGGTGCTTGCTATAGTCCAAAACACCTATTTGCTTTGCCTCCTGATGCTAGTTCTCTCTTGCTGGATTCTTTCAACTTTGTTGAGGAGAGTGGTGATCAGGATCTGCTGCTAGAGGTGCCATCTAACACTTCTTTTCCACAGGCAGAGCTCTTGCACCCAACTTTAAGCCTTGGTGCTCAAGCCAGCACTGGTAGCAGCTCAGTCTACTCAAATCTTGTTCACCATTAA

>GmGATA37

ATGCATCGCTGTTGCAGTGGTTCCCAGGGGCACGTGATGGGACCCTGCACATGTGGTATGTTTCACAGCCAAACCACCAGCTCCTTCGCCATGTTCTTCTCCATGCCCAACCACAAACCACCACCCTACGATGATTCCGACAACATTTATGACTATTCTTCCTTCACTCCCTCTTCCTCTTCTTCCGTTGACTGCACCCTCTCCCTCGGTACCCCTTCCACCCGCTTCTCCGAAGACGAAGAAAAACGAAGCCGCCACGAACGCCGTTCCGTCTCTAACTTCTGCTGGGACTTACTACAATCCAAACACAACAACCCTCAATCCCATTCCAAGTCCTCCCGAACCACCAACACCACCGACCCTCTCCTCGCTCGCCGCTGCGCCAATTGTGATACCACTTCTACTCCTCTCTGGAGAAATGGCCCCCGTGGCCCCAAGTCACTGTGCAATGCGTGTGGGATTAGATTCAAGAAGGAGGAGAGAAGAGCGAGTGCGGCCGCCGCCACGCCGGCATCGGCGGCTTCCGGTGGCGTTATGGAATCGGCGCAGGTGTACAATAACTCGTGGTACGCACACCAGCAGAGTCAGAAGATGCAGTGCTTCTCGCCGGGGATGGGGAACGAGTTCCGTTTCGTGGATGACGCCGATAGAGACGCCGCCGATAACGGCATTCCGTTCCTCTCGTGGAGACTCAACGTCACGGACAGAACGAGCCTGGTTCACGACTTCACGAGATGA

>GmGATA38

ATGGAAGTTGCGGCGGCGAAAGCGCTGAAACCAAGCTTGCGGACAGAGTTTATTTTCCCTCAAGCAATTTACGACGAGATTTTATGTTTTAACGCGAACAACGTTGTTGCCGATGAAGATTTCTCCATGGACGACTTACTTGACTTTTCCAACGGGGAATTCCAAGTTGGAAAAGACTTCGATGATTACGAGGAAGAGGAAGACGAGGAAAAAAACAGCACCTCCGGTTCCTTGCAGTCGCAGGACAGAGCCGAAGACGACAACAATTCTAATTCCACCGCTGGAGGCGGCGGCCACGACTATGTTTTCGCCGGCGAGTTGTCAGTTCCGGCAGATGATGTGGCAGACTTGGAATGGGTTTCTCACTTTGTGGATGATTCTCTCCCGGAGTTATCTATATTGTACCCCATTCATTGTTCCAAGAAAACAAGAGTATGGGCCGAACCGGAATCCAGATTGAGCCCGGCCCAAACCGTTTCAAAAGTCCCGAGAAAGTCGAGAACTGAAAAGCCTAGAAAGCCCAACACTCGTGTCTGGTCCTCTTTTACGGTGTTTGCTGGCTCCGTTGGGTTCGGCGAGTTGGTGACTAAGAAGCAGAAGAAAAAGGTTGAGGCCCAAAGCGGTGGGGCCCAGTCTCTGCGACGTTGTAGCCACTGCCAGGTGCAGAAAACACCGCAATGGAGGATCGGTCCACTAGGGCCCAAAACACTTTGCAACGCTTGCGGGGTTCGATTCAAGTCTGGTCGGCTTTTTCCAGAGTATAGACCGGCCTGTAGCCCGACCTTTTGTGGCCACATTCACTCTAACAACCATCGTAGAGTGTTGGAGATGAGATGGAAAAAACAGATAGCAGAATCCGTTACTGGTTCAGACCGGAAACAATTGATCCCAAATTATTAA

>GmGATA39

ATGAAGGACTGTTGGTTTTTTGACAACAATTTCAATGGTCTGTCGGATGAGAGTCTTGATGATGTGATGGACATGGAGCTTTTGGATTTGCCCCTTGACTTTGAGGATGTGGAAACTGATGCTGTGGAAGAACAAGATTGGGATGCTCAGCTCAAACTCCTTGAAGACCCACCACCACCACTTGGAGTTTTCCCACTACAACAATCCTCTGCATTCTGTGGACAAACTCGGAATGAGAATGCGAAACTCGGCAGCAAGAGTTTCTCTGCTTCTCTAGCGAAGACTGTCAGACCTGCATATGGCAAAACTATTCCTGTCCAGAAAGTCTCTTTAAAAGGAAAAGATTTGCTCCAATTCCAAACCAACAGCCCAGTTTCTGTTTTTGAAAGCAGTAGTAGTTCTCCCTCGGTTGAGAACTCCAACTTTGAGTTACCTGTCATCCCAACAAAGCGTCCCCGTACCAAACGCCGGCGTCTCTCAAACATCAGCCTGCTTTACTCTATTCCTTTTATCCTTACTTCACCGGCTTTTCAAAAATTTCAGAGGATGGATTTCTCCAAATCAGATATACAAACACAGCCTTCTGGGGAATTATTATGCAAGTTCAAAAAGAAGCAGAGGAAAAAGGATATTCCCCTGCCGACTAACAAAATTGAGATGAAGAGATCCTCATCACAGGAGTCAGTTGCACCCAGAAAATGCTTGCATTGTGAGGTGACAAAGACGCCGCAATGGAGAGAAGGACCAATGGGTCCTAAGACCCTATGCAATGCCTGTGGGGTTCGATACCGGTCTGGCCGGCTCTTTGCTGAATACCGGCCTGCATCTAGCCCCACTTTTGTAGCATCACTGCACTCAAACTCTCACAAGAAGGTCTTAGAAATTAGGAACAGAGCCACCCAGGTGACTGTTAGATAA

>GmGATA40

ATGACCTTGATCACTCCCTCTTCCTCTTCTTCCGTTGACTGCACCCTCTCCCTCGGTACCCCTTCCACCCGCTTCTCCAAATACGAAGAAAAACGAAGTTGTCACGAACGTCGTTCTGTCTCTAACTTCTGTTGGGACTTACTACAATCCAAACACAACAACCCTCAATCCCATTCCAAGTCCTCCCAAATCACCAACACCACCGACCCTGTCCTCGTTCACCGCTGCGCCAATTGTGATACCACTTATAATCCTCTCTGGAGAAATGGCCCCCATGGCCCCAAGTCACTGTGCAATGCATGTGGGATTAGATTCAAGAAGGAGGAGAGAAGAGCGAGTGCAGCCACTGCCAAGATGCAGTGCTTCTCGCCGAGGATGGGGAACGAATTTCGTTTCATGGATGACGCTGATAGAGTTACCGCCGATAACGGCATTTCGTTCCTCTCGTGGAGACTCAACGTCACAGATTGA

>GmGATA41

ATGGAAGTTGCGGCAGCGAAAGCCCTGAAACCAAGCCTTCGGACAGAGTTCATTTTCCCTCAAGCAATTTACGACGAGATTTTGTGTTTTAACGCCAACAACGTTGTTGCCGGCGAAGATTTCTCCGTGGACGACCTACTCGACTTCTCCAACGGCGAATTTCAAGTCGGGAAAGATTTCGATGACTACGAGGAAGACGAAGACGAAGAAAAAGGCAGCACCTCCGGTTCCTTGCAGTCGCAGGACAGAACCGAAGACGACAGCAACTCTAATTCCACCGCCGGAGGCGGCGGAGACTCCGTTTTCGCCGGCGAGTTGTCAGTTCCGGCGGATGACGTGGCAGACTTGGAATGGGTTTCTCACTTTGTGGACGATTCTCTCCCGGAGTTATCTCTATTGTACCCAGTCCGTTGTTCCGAGCAAACAAGGGTATGTACCGAACCGGAACCCAGACCGGGCTCGGTCCAAACCATTCCAGCAGTTCCAAGAAAGCCGAGAACCGGAAAGACTAGAAAGCCCAACGCTCGTGTCTGGTCCTCGATGTCGTCTTTGTGTTCTTCGGTGACGGCCAAGAAACAGAAGAAAAAGGTCGAGGCCCAAAACGGTGGGGCCCAGTCTCTGCGACGGTGCAGCCACTGCCAGGTGCAAAAAACGCCACAGTGGAGAACCGGCCCACTAGGGCCCAAAACACTTTGCAACGCTTGTGGAGTTCGGTTCAAGTCCGGTCGACTTTTTCCAGAGTATAGACCAGCCTGTAGCCCAACTTTTTCTGACGATATTCACTCCAACAGCCATCGTAAAGTGTTGGAGATGAGACGAAAAAAGGAGATAGTTGAATCGGACCGGATTCAATTGATCCCAAGTTGTTAA

>GmGATA42

ATGAAGGACTGTTGGTTTTTTTACAATAATTTTAATGGTCTGTCGGATGAGAGTCTCGATGATGTGATGGACATGGAGTTTTTGGATTTGCCCCTTGACTTTGAGGATGTGGAAACTGATGCTGTGGAGGAACAAGATTGGGATGCTCAGTTCAACAAATTCCTTGAAGACCCACCCCCACCACTTGGATCATTTCCACTACAATCCTCTGAGTTCTGTGGACAAACTCAACATGAGAATGTGAAACTCGGCAAGAGCTTTCGTGCTTCTCTACCAAAGACTGTCAGACCTACATATGGCAAAACTATTCCTATCCAGAATGTCTCTTTAAAAGGAAAAGATTTGCTCCAATTCCAAACCAACAGCCCAATTTCTGTTTTTGAAAGCAGTAGTAGTTCTCCCTCGGTTGAGAACTCCAACTTTGAGTTACCTGTCATCCCAACAAAGCGTCCCCGAAACAAACGCCAGCGTCTCTCAAACATCAGCCTGCTTTTCTCTATTCCTTTTATCCTTACTTCACCGACTTTTCAAAAATGTCAGAGGATGATTTTCTCCGAATCAGATTTACAAACACAACCTGCTGGGGAATTATTGTGCATGGTCTCAAAGAAGCTGAGGAAAAAGGATATTCCCATGCTAGCTAACAGAATTGAGATGAAGAGATCCTCGTCACAGGAGTCAGTTGCACTCAGGAAATGCTTACATTGTGAGGTGACAAAGACGCCGCAATGGAGAGAGGGACCAATGGGTCCTAAGACCCTATGCAACGCCTGTGGGGTTCGATACCGATCTGGCCGGCTATTTGCTGAATACCGGCCTGCAGCTAGCCCCACTTTTGTATCATCATTGCACTCAGACTCTCACAAGAAGGTTTTAGAAATTAGGAACAGAGCCACCCAAGTGACTGTTAGATAA

>GmGATA43

ATGTCTAAGGATATTGCTAACATGAAGGACTCTTGGTTTTTCGACAATAACTTTAATGGTCTGTCAGATGAGATTTTTGATGATGTCATCAACTTTTTTGATTTCCCGCTGGAAGACGTGGATGCTAATGGTGTGGAAGAAGACTGGGATGCTCAATTAAAATGCCTTGAAGACCCGCGTTTTGATGTTTATTCAGCATCATCAGCTGGGCTGTGTGCCGAAACTCAAAATGAGAAGCCCCAACTTGGAATGAAGTTATCTGCTTCTAGTAATGGGATTTCCCCAATAAAACAGCTGGCAAAAGCTCCTGGACCAGCATATGGAAAGACCATTCCCCACCAGAATGTCACTTCCAATGGAAAAGATTTGCATCAATTCCAAACCTACACCTACAGCCCAGTTTCGGTTTTTGAAAGTAGCAGTTCGTCCTCAGTTGAGAATTCCAACTTCGATCGACCTGTCATCCCAGTGAAGCGGGCTCGTAGTAAACGTCAGCGTCCTTCAAACTTCAGTCCTCTATTTTCAATTCCTCTCATCGTTAATTTGCCGGCTGTGCGAAAAGATCAAAGGACAGCAGCCTCTGACTCAGATTTTGGAACAAATGTTGCTGGGAATCTATCAAACAAAGTAAAAAAGCAGAGGAAAAAGGATTTGTCCCTGCTATCAGATGTTGAGATGACGAGATCCTCATCACCAGAGTCAGGTCCCCCCAGAAAATGCATGCATTGTGAGGTGACAAAAACCCCACAATGGAGAGAGGGACCTATGGGTCCCAAAACACTGTGCAATGCTTGTGGTGTTCGATACAGGTCCGGCCGCCTCTTTCCTGAATACCGGCCGGCAGCTAGCCCGACTTTTGTAGCATCGCTGCACTCAAACTGCCACAAGAAGGTTGTGGAGATGAGAAGCAGAGTCATCCAGGAGCCTGTTAGGTGTTCTATGTTGGCTTCATCAAATCTCCATGGAAATTCTGTAGGATAA

>GmGATA44

ATGATTCCAGCCTATCGCCACTCAGTATCTTCTGTTATGCCTCTGGATCTTAATGAAGATCAAAACCACGAGTTCTTCAGTCCAATTCATCACCCTTCCTCTTCGTTTTCTTCTCTATCTTCATCATATCCTATTCTCTTCAACCCGCCAAATCAAGATCAAGAAGCTCGATCATACGACTGGGAAACAACAAAGCACTTACCAAGTCATGAAGAAGAGGCTGAGAAGATTATCCCTACTAGTGGATCATGGGGTCACTCGGTGGAAGAAAGTGAGCATAAGGTGACAGTTTGGAGAAAAGAAGAGAGGAATGAAAATCTTGCTGAAGATGGTTCGGTGAAGTGGATGCCTTCGAAGATGAGAATTATGCGGAAGATGTTGGTGTCCAATCAAACTGATGCATACACTTCAGACAACAACACTACGCACAAGTTTGATGATCATAAACAACAACTGTCGTCACCGCTTGGAATTGATGATAACAGCAGCAACAACTATTCAGACAAAAGTAACAACAGTATTGTTAGGGTTTGTTCTGATTGCCACACCACCAAGACTCCTCTATGGAGGAGTGGACCAAGAGGCCCCAAGTCGCTTTGCAATGCCTGCGGAATTCGACAAAGGAAGGCAAGACGAGCCATGGCAGCTGCTGCGGCGGCAGCATTGGGAGATGGAGCAGTTATTGTGGAAGCTGAGAAATCTGTGAAGGGAAAGAAGTTGCAGAAGAAGAAAGAGAAGAAGACAAGAATTGAGGGTGCAGCTCAGATGAAAATGAAGCGGAAGCTTGGAGTTGGAGCAAAGGCATCACAAAGTAGAAACAAGTTTGGTTTTGAGGATTTGACATTGCGCTTGAGAAAGAACTTGGCTATGCATCAAGTTTTCCCTCAGGACGAGAAGGAGGCTGCGATCCTCCTCATGGCTTTATCTTATGGCCTTGTTCATTGA

>GmGATA45

ATGTCTAAGGATATTGCTAACATGAAGGACTCTTGGTTTTTCGACAATAACTTTAATGGTCTCTCAGACGAGATTTTTGATGATGTCATTAACTTTTTTGATTTCCCGCTGGAAGACGTGGAAGCTAATGGCGTCGAAGAAGACTGGGATGCTCAATTAAAATGCCTTGAAGACCCGCGTGTTGATGTTTATACAGCATCATCAGCTGGGTTGTGTGCCAAAACTCAAAATGAAAAGCCCCAACTTGGAATGAAGTTCTCTGCTTCTGGAAATGGGATTTCCCCAATAAAACAGCTGGGAAAAGCTACTGGACCAGTATATGGAAAGACCATTACTCACCAGAATGTCACTTCCAATGGAAAAGATTTGCATCAATTCCAAACCTACACCTACAGCCCAGTTTCGGTTTTTGAAAGCAGCAGTTCGTCCTCAGTTGAGAATTCCAACTTTGATCGACCTGTCATCCCAGTAAAGCGGGCTCGTAGTAAACGTCAGCGTCCTTCAAGCTTCAGTCCTCTATTTTCAATTCCTTTCATCCTTAATTCGCCGGCTATGCAAAATCATCAAAGGATAGCAGCTGCTGACTCAGATTTTGGAACAAATGTTGCTGGGAATCTATCAAACAAACTAAAAAAGCAGAAAAAAAAGGATTCGTCCCTGCTATCAGATGATGTTGAGATGATGAGATCCTCATCACCGGAGTCAGGTTCCCCCAGAAAATGCATGCATTGTGAGGTGACAAAAACCCCACAATGGAGAGAGGGACCTGTGGGTCCCAAAACACTGTGCAATGCTTGTGGTGTTCGATACCGGTCTGGCCGCCTCTTTCCTGAATACCGACCGGCAGCTAGCCCGACTTTTGTAGCATCACTGCACTCAAACTGTCACAAGAAGGTTGTGGAGATGAGAAGCAGAGCCATCCAGGAGCCTGTTAGGGGTTCTATGTTGGCTTCATCAAATCTCCATGGAAATGCTGTAGGATAA

>GmGATA46

ATGACTCCTTATTCTCTGAACCCACCAGGCCCTTCCATACAAGCTGGTCAAAACCAACTCTTCAATATTTCTCCTAATAATCAGGACTGCCGTACCTTTTTTAACATATTTGATCCAAGGCAAACCAGCATAGAAATTGGAGGGTTAAGAGAAAATTATCGACAGGATGACAAGATGATATTGCATGATGGATCATCAAGCAACTGCAACTCGTCCTTCAATATTTCACCTGAGACAGTAGTTATGGTTGATCCATTAAGCAGCGCATGTGATCGGCGTAATTTACCATCTGAGGAAGAGAGCAAAAATAACGATCACGGGTCTGGGAACAAATGGATGTCTTCAAAGATGAGGTTAATGAAAAAAATGATGAGACCGAGTATCAGTCCAACCACTGACAAAGCAATCAATTCAAGCCCAAGATTCCAAAATCATCAAGGGCTCGAAAGCAGAAGGTACAGCCAAAGAAGCCCTCGTAACAACAACGGCAGTAGCACCCCTAGGGTTTGTTCGGATTGCAATACAAGCACTACCCCACTTTGGAGGACTGGCCCTAAGGGTCCCAAGTCTCTTTGCAATGCCTGTGGCATTAGACAGAGGAAGGCAAGAAGGGCAATGGCGGAAGCTGCAAATGGTTTGGTCACTCCCATAGCATGTGAAAAGACCAGACTACACAACAAGGAAAAGAAGTCTCGTATGAACCATTTTGCACAGTTCAAGAACAAGTACAAGTCCACTACTACTACTACTACTACTACTGTAGGCTCATCTGAGGGAGTGAGGAAGCTTGAATATTTCAACAACTTCGCCATAAGTTTGAGGAGTAACAACTCTGATTTTGAACAAATGTTTCCACGGGATGAAGTAGCTGAGGCGGCATTGCTTCTGATGGACTTATCTTGTGGTTTTGTCCACTTATAA

>GmGATA47

ATGGAATCACCCAACTCTTCCCCAATTTTCCCACAATTCACCTTCGACACCAACAAGAACAACAACAACCCGGACAACTTCATTGTGGAGGACCTCTTGGACTTCTCCAACGACGACGTTGTCATCACCGACGCCACCTTCGACTCCATCACGACCGACTCTTCCACCGTCACCACCGTCGTTGACAGCTGCAACTCCTCCTCCTTCTCTGGCTCCGACCCCAATACCGTCCCTGATGTCGGTAGCCAGAATCTATCCGACGGCCATTTCTCCGGTGACCTCTGCGTTCCGTATGATGACATCGCGGAGTTAGAATGGCTTTCGAATTTCGTGGAGGAGTCGTTTTCGAGCGAGGACCTGCAGCAGATGCAGCTGATATCAGGCATGAATGCGCGAAACTACGACGTATCAGAGGCCCGCGAGTTCCACTACGAGCCCACCACCAGAAGCGGGCCCCACACCCCGGAGCCCACCACCAAAAGCGGCGGGCTCCACTATGAGCCCACCAGAAACAGCCCAATATTCAATTCGGAAGTGTCGGTTCCAGCCAAGGCCCGCAGCAAGAGGTCCCGCGGGCCCCCATGCAACTGGGCCTCGCGCCTCCTCGTCCTGTCCCCGACAACCTCATCGTCCTCGGACTCCGAGGTCACCGTTCCTGCTCCCGCCGAGCACGGGCCGGCCCCAGCAAAAAAGGCCGCAAAGGCCGGGCCGAGGAAGAAGGACAGCGGCAGCGATGGCAACGGCAGTGGCGGGGACGGGCGCAGGTGCTTGCACTGCGCCACGGACAAGACCCCGCAGTGGCGGACCGGGCCCATGGGACCGAAGACTCTGTGCAACGCTTGTGGCGTGAGGTTCAAGTCGGGCAGGCTGGTGCCCGAGTACAGGCCCGCGGCGAGCCCGACTTTTGTTCTGACTAAGCACTCCAACTCGCACCGCAAGGTGCTGGAGCTGCGAAGGCAGAAGGAAATGGTGCGGGCCCAGCAACACCACCAGCAGCACCACCAGCAACAACAACAGTTCCTACACCACCACCACCATAATCATAACCATCACCATCATCAACATCATCAGAACATGATGTTTGATGTATCCAACGGTGACGATTACTTGATCCACCAACCCGTGGGCCCCGATTTCAGGCAGCTTATCTAG

>GmGATA48

TGTTTTTCAAGCCCAGCAAGCTGTGTCTTGGTACCTGTTGGTGTTAAGACTACAAGTACAAAAAGCCTAAGCACAAGCATCAACCCTTCTTTGAAGAGACCTCAACAACAAAATGAGCCACATTTGCAAAACTTTGTTGTGCCAGGGAAGCCAAGGAGCAAAAGGAAGAGGCTTTCAGAACCAAGAACCAACAAAGACCCTCTAAGCATATGGTCACACCACTTGAACCCTCAAATTGAGGCCTTGTGTTCTGACCCTCCTCTACTCAAACAGGCTTATTGGTTGGTAGACAGTGAACTCATCATGCCAAAGCCAAAGGATAACAAGGAGCAAAAAGAAGAGGTTGTGATCATGACCAAAAAGGATGAAGAAAAAGTGATTATTAATAGGACCCCACAATGGAGGGTAAAACCATTAGGTCCAAAAACACTATGCAAAGCATGTGGAGTGAGGTACAAGTCTGGTAGGTTGCTACCAGAGTATAGGCCATCCAAGAGTCCTACTTTTGTGAGCTACTTGCACTCCAATTCCCACAAAAAGTCATGGAGATGA

>GmGATA49

ATGGAGCCATCAGCAATGTACGGACACTCCCAGCCCCTGAGCATGCCCTCGCAGATCGGCGGCGGCGAAAGCGACGACGGCTCCGGAAACGAGCACGCCGTCGACGGCCACCACCACCACATTCAGTACGAAACGCACGCGCTCGAGGACGGTGCCGCCGTCGTCGTCGAGGATGTTACCTCGGATGCGGTCTATGTCTCCGGCGGTGGAGGCCCCGTAGAGTCCAGCCAGCTTACGCTGTCGTTTCGTGGCCAAGTGTACGTCTTCGATGCCGTTACGCCTGATAAGGTTCAAGCGGTGTTGTTACTGTTGGGTGGATGTGAACTATCTTCAGGTGGTTCGCCATGTGTGGATCCGGGGGCTCAACATAATCAAAGGGGTTCAATGGAATTTCCTAAATGTAGTCTACCACACCGAGCTGCCTCATTACATAGGTTCAGGCAGAAGAGGAAAGAGCGATGCTTTGACAAAAAAGTGAGATATAGCGTGCGGCAAGAAGTTGCGCTCAGGATGCACCGTAACAAGGGCCAGTTTACTTCATCTAAGAAACAAGATGGAGCTAATAGTTATGGTACTGACCAAGATTCAGGACAGGATGATAGTCAATCCGAAACCTCATGTACACATTGTGGCATTAGTTCAAAATCCACCCCAATGATGCGGCGAGGGCCATCTGGTCCAAGGTCACTTTGCAACGCTTGTGGGCTTTTTTGGGCAAATAGGGGTGCTTTGCGAGACCTTTCTAAGAGAAATCAGGAACACTCTCTTCCACCAGTTGAGCAGGTTGATGAAGGTAATGACTCGGACTGTCGGACTGCCACTGCCGACCCTGCACATAACAATCTTCCTGCTTTCTCAGAGCATGATAATCCAGCTTTGGTAGCTGATCATAAAGTTTTCCAGTCTCAGAAAATGTTGAAGTAG

>GmGATA50

ATGGATTTTGGAAAGACTAATGAAGCATCCAATTCAAAGCTGGTTCATGACTTCGACTTGAACATTGCATATGTGGAGGAATTTGATCATGTGAATGCTGAAAACGAGTTTTCATCGCCCATTCTCGTGAATACTACTCAGCAGGCTTGTAAAAATAGCATTGAAAACATGAACATAGAGGATGCAATTGCATATAACCGCGAGACAACTATTCAAGCAAATTCTGCTGCTAAGGGAGCTACCTCTGAAGATACGAGGCAAGTTGAACCCAAGTATATTGCCTCTGTCTCCTTTACACCAGCAAGAATCTCTCAATACCTTCGTCGTCGCCGCCACCATCGAGGTGCAGAATCTAAGCAGAGTACTGATCCAGATAAACTCTGCACTAATTTTTATTGTAAAACAAGAAAAACTCCCATGTGGCGCAAGGGACCTCTGGGACCAAAGACTTTGTGCAATGCTTGTGGCCTTCAATACCTTAAGATGGTGAAGGGTACAGGCAGTGGCCTCCCTGCGGCAGTTTCAGATGAAGGTGATGCTTCTGTCCCAGCTGAAACCGTGGAAAGGGATTCAAATCTCTAA

>GmGATA51

ATGATACCAACTTATCGTTACTCAGTGTCTTCACCTATGCCTATAGATCTTAATGAAGATCACACCCACCACCTCTTCAGTACAAATCATCAAGCCTCTTGTTCATCTTCTTCGCTATCTTATTCTATTCTCTTCAACCCGGATCAAGATCAGGGAGGATCTTGTAGCGACTGGAAATCCAAGCATTTGCAAAGTGATGAAGAGGCCCAGAAGATTGTTCCTTCTAGTGGATTATCCGAAAAGGATGAAAACAAAAGTGATCTCAAGCTGAGGGTTTGGAAGAAAGAAGATAAGTGTGAAAATTTTCAAGGTGAAGATAATTCAACCAAGTGGATGCCTTTAAAGATGAGAATGATGCGGAGGCTGATGGTGTCAGATCAAACGGGTTCTGATGATACAGAAGGCATGATCTCTAACTCTCAGAAGATCAAGTATGAAGAGAAAAACTCACCACTGTCCCCTCTAGGAACTGATGATAGTAACTACAATTCTTCTTCAAACCACAGCAATATCACTGTTAGGGTTTGTTCTGATTGCCACACCACTAAGACCCCTCTCTGGAGAAGTGGACCAAAAGGTCCAAAGTCACTTTGCAATGCTTGTGGAATTCGACAAAGGAAGGTACGACGTGCCATTGCCGCCGCTGCAACGTCAAACGGAACAAATCCGGTGGAGGCTGAGAAATCTCAAGTGAAGAAGGGAAACACGTTGCATAGCAAAGGGATGAAGTCCAAAACTGAGGGTGCACAACAGATGAAAAAGAATCGTAAACTCGGAGCCAGGTATAGAAAGAGGTTTGGTGCTTTTGAGGATTTGACAGTGAGATTGAGTAAGAACTTCGCTCTCCAGCAAGTTTTCCCTCAGGATGAGAAGGAGGCTGCAATCTTGCTCATGGCTTTATCTTATGGCCTTCTTCATGGCTTTCCCACGGATCGTTATATCACTTAG

>GmGATA52

ATGAACATGGATATGTGCCAAAATGTATCAGTTTCCGGTGAGTGCCAACAAGTGCAGGTTTTTGCCCCTTCTTGCTCAAGCAGCCTTGATGACCTCTTCTCTGCTCAGAACACGGAAGTGGATGTTGAGTTGGAGTGGCTTTCAGAATTTGTTGAAGACTGTTTTTCAAGCCCACCAAGCTGTGTCTTGGTACCTGTTGGTGTTAAGACTACAAGTACAAAAAGCACAAGCACAAGCATCAACCCTTCTTTGAAGAGACCTCAACAACAAAATGAGCCACCTTTGCAAAACTTTGCTGTGCCAGGGAAGGCAAGGAGCAAAAGGAAGAGGCTTTCAGCACCAAGAACCAACAAAGACCCTCTAAGCATATGGTCACACCATTTGAACCCTCAAAATGAGGCCTTGTGTTCTGACCCTCCTCTACTCAAACAGGCTTATTGGTTGGCAGACAGTGAACTCATCATGCCAAAGCCAAAGGATAAGGAGGAGCAACAAGAAGAGGTTGTGATCATGGCCAAAGAGGATGAAGAAAAAGTGATTATTAATGTGAGCAAGGAAATAAGCTTTGGGGACTCTGAACTTGATGAGGGTAGCAATGGTCAACAACAACCAATGCCAAGAAGGTGCACACATTGCTTGGCTCAGAGGACCCCACAGTGGAGGGCAGGACCATTAGGTCCAAAGACACTATGCAATGCATGTGGAGTGAGGTACAAGTCTGGTAGGTTGCTACCAGAGTATAGGCCAGCCAAGAGTCCTACTTTTGTGAGCTACTTGCACTCCAATTCCCACAAGAAAGTCATGGAGATGAGGATGTCTGTTTACTCCATTTCTAGTGAGCAGTAG

>GmGATA53

ATGGGCAAAGTCCTTGGCAATATTCAATTCTACGTTATGGCCACCCTTTTCATCAACACCGAACTCACTGTTCCGGCGGAGGAGGAAGTTGCGGACTTGGAATGGTTGTCTCATTTCGTCGAAGATTCTAATTTCTCAGAATACTCTCTTCCCTTCCCTGCAACCTTAGCGGAGAAAGTGAAATCACCGGAACCGGGAAACACCGGTTTCACTTACAAGACCTCGGTTCCGACGAAGACGAGGAGCAAGCCAACGCGAACCAGTGTCCGGGTTTGGCCGCTCACGTCATCAACAGTAACAACAACAACCCCGACAACTTCATCCCCGTCCTCATCTTCGCCCTCGAGTCCGTTGCTTGCGTACGCAGCAGCGGACCCGAGGGTGAAGAAACACGTTGTTATTGACAGCGCCGTGGCGGCGCGACGGTGCAATCACTGCGGAGTACAGAAAACACCACAGTGGCGCATCGGGCCACTCGGGGCGAAGACCCTGTGCAACGCGTGTGGGGTCCGCTTCAAGTCGGGTCGGTTATTACCTGAATACAGACCCGCCTGTAGCCCTACTTTCTCCATCAAGTTGCACTCCAACCACCACCGGAAGGTCCTCGAGATGCGGCGGAAGAAGGAGGTCACGCCGGAACCCGACACCTCTTCGCCACGGTCGATTCCCAATTTTTGA

>GmGATA54 ATGATGCATCATTGTTGTGGAAGCTCCCAGGGGCACGTGATGGGCACGTGCACATGCGGCATGTATCACAGCGAAACCAGCGCTTATGGCTCGATGCTGTTTTCCGTGCCCAACAACAGCGAATATGACATGTATTCGTCCTTCACGCCCTCTCCTTCCTCCGTGGACTGCACGCTCTCCCTCGGAACACCCTCCACGCGTTTAACCCAAGACGACGACCATGACAACGACAACAAACGACACCCCCACCAACGTCGTTCTGGAGTCGCCAATTTTTGCTGGGACCTGCTCCATTCCAAACACAACAACAACAACACGCAATCTCAAAGCAAGTCTAGTAGCAGAGGAAGCAGCAGCAACAACAACAACAACAACGACCCTCTCCTCGCTCGTCGTTGCGCCAACTGCGACACCACTTCCACACCCTTATGGAGGAACGGTCCTCGTGGTCCTAAGTCACTATGCAATGCTTGCGGGATCAGATTCAAGAAGGAAGAGAGAAGAGCGAGCGCCGCCGCCGCCACGTCAACGGCGGTTCCCGAGGGCGAGATGGAATTGGCCCGCGTGTACGGCCACCACCACAACAATTCGTGGTACGCCGCACACTCGCAGAACCAGAAGATGATGATGATGGGTAACGAGTTACGCTTCATGGACGATTCCGAAGACAGGGATTCGGAGAATAACGGGATTCCCTTTCTCTCTTGGAAACTGAACGTTCCAGATCGAACGAGCCTCGTTGATGAACGATGGTGA

>GmGATA55

ATGGACATGGATGTTTGCCGAAATATATCAGTTTCTTCAAGTGAGTGTCAGCAAGAGCTTCCCACCCTTGATGACCTTTTCTGTCATCAGAACACGGAAGTGGATTTTGGCATGGAATGGTTATCAGTGTTTGTGGAAGACTGTTTCTCTAGTAGGCCAAGTTGCCTCTTGCCACCCTCTGGTGGTGGTGTTCAAACCACAAGCACTAGCACCAAGCCTTCTTCAGGCACAATAATGCCAAGACCCCAACAAAGTCATCACTGTCCTTTGCAGAATTTTGCTGTGCCAGGGAAAGCAAGGAGCAAGAGAAAGAGACTTTCAGCCCCTAGAACAACAAAACACACCCTAAGCACATGGTCACAACATTTCAGCTCACAGAATGATGGGGTGAGTTCTGACCCTCCTCTCTTGAAACAAGCATATTGGTTGGCTGATAGTGAACTCATAGTCCCCAAGAAGAAGGATGTTGAGCAAGAAGAGGGGGTTGTTGTGGTGGTGAAGAAGGAGAAATTGGGGGATTATTATGATGATGATGAGGGTGATGAAGTTAACAACAATAATACCAACAATAATAATAATGATAATGTGCAGCACCCAATTCCAAGAAGGTGCACACATTGTCTAGCTCAGAGGACCCCACAGTGGAGGGCAGGACCATTGGGTCCAAAGACACTATGCAATGCATGTGGGGTGAGGTACAAGTCTGGAAGGTTGTTGCCAGAGTATAGGCCAGCCAAGAGCCCTACTTTTGTAAGCTATTTGCATTCCAATTCACACAAGAAAGTCATGGAGATGAGAATGGGTGTTGGTGTTGCTGTTCTTTCTACAGACAAGAAGTAG

>GmGATA56

ATGATCGGAAACTTCATCGACGACATTGACTGCGGCAACTTCTTCGACCACATCGACGACCTCCTCGAATTCCCCGACGACGCCGCCGCCGCTGACACCTCCGCCGCCGCTCCTGTCCCTCCACCGGCGAACTTCTGGTCCGCCGAGTCCGACTCGCTCCCCGCCACCGACACGGTGTTTTCCGACAACTCCGTGACGGACCTCTCGGCGGAGCTCTCTGTTCCGTATGAAGACATTATGCAATTGGAATGGCTGTCCAATTTTGTTGAGGACTCTTTCTCTGGTGGGAGCATGACCATGAAGAAAGAGGAGCCACAATGCACCACCACCAAGGAGGACATAGCTCCTGCCCAGTTCCAGACAGCAAGCCCAGTATCAGTCCTTGAAAGCAGCAGTTTCTGCTCTGGGGAGAAGGCTGGCACGGAGATTAACATCTCCGTGCCATGTGGACGCGCGCGCAGCAAGCGCCCGCGTCCTGCAACCTTCAACCCCAATCCAGTGATGCAGCTCATCTCCCCTGCATCCTCCACTGGTGAGAACACGCAGCACAACGCTGCCAACACCTCCAAGGCATCATCGGATTCCGAGAATTTCGCTGAGTCGGTGATCAAGGCTCCTAAGCAGGCCTCTGGGGAGCACAAGAAGAAAAAGAAGATCAAAGTGACGTTCCCATCAGGTCAAGAGCGGAATGCACCATCACAGGCAATTAGGAAATGCTTGCACTGTGAGATAACCAAGACACCACAGTGGAGGGCAGGGCCAATGGGGCCGAAAACACTCTGCAATGCTTGTGGCGTGCGCTACAAGTCAGGCCGGCTTTTCCCCGAATATCGCCCTGCAGCGAGTCCAACGTTTTGTGCGGCCATGCACTCCAACTCCCATAAGAAGGTCCTTGAAATGAGGAACAAGACAGGCACCAAATCTGGCTTTGCAACTGTTTCTGCTGCCTCACCAGAACTCATTCCAAACACTAACAGCAGCCTTACCCTTGAATATATGTGA

>GmGATA57

ATGGTGGATCCAACTGGAAAAGGATCGGAGGTTGAAGTTGAGGACTCAAACTCAAACCCTAACGCTCCTTCCTCAGGGAACAGTCCGAGCAGCAACAATGAGCAGAAGAAAACCTGCGCCGATTGCGGCACCACCAAGACCCCTCTCTGGAGGGGTGGTCCTGCAGGACCTAAGTCTCTGTGCAACGCGTGCGGGATCAGAAGCAGGAAGAAGAAGAGAGCGATCCTGGGAATCAACAAGGGGAGCACCGAGGACGGAAGGAAAGGAAAGAGGACCGGCGGAGGCGGCGGAATTGGCGGAATTGGCGGTGGCGCGTTGGGGAGGGAAGTGTTGTTGCACCGATCGCATTGGAAGAAGCTAGGAGAGGAAGAGAAAGCTGCGGTGTTGTTGATGTCGCTCTCTTATGGATCCGTTTATGCCTGA

>GmGATA58 ATGATTCCAGCCTATCGCCACTCAGTATCTTCTGTTATGCCTCTGGATCTTAATGAAGATCAAAACCACGAGTTCTTCAGTCCAACTCACCACCCTTCCTCTTCGTTTTCTTCTCTATCTTCATATCCTATTCTCTTCAACCCGCCAAATCAAGATCAAGAAGCTCGATCATACTACTGGGAACCAACAAAGCAGTACTTACCAAGTCATGAAGAAGAGACTGAGAAGATTATTCCTTCTAGCGGATCATGGGATCACTCGGTGGCAGAAAGTGAGCACAATAAGGCGACAGTTTGGAAGAAAGCAGAAGAGAGGAATGAAAATCTTGAATCAGTTGCTGCTGAAGATGGTTCGTTGAAGTGGATGCCTGCCAAGATGAGAATTATGCGGAAGATGTTGGTGTCGGATCAAACTGATACATATACTAATTCAGACAACAACACTACGCACAAGTTTGATGATCAGAAACAACAACTGTCGTCACCGCTTGGAACTGATAACAGCAGCAGCAACAACTATTCAAACCACAGTAACAACACTGTTAGGGTTTGTTCTGATTGCCACACCACCAAGACTCCTCTATGGAGGAGTGGACCAAGAGGCCCCAAGTCACTTTGCAACGCCTGTGGGATTCGACAAAGGAAGGCAAGACGAGCCATGGCAGCTGCTGCGGCTTCTGCATCGGGAAATGGAACAGTAATTGTGGAAGCTAAGAAATCTGTGAAGGGACGGAACAAGTTGCAGAAGAAGAAAGAGAAGAAGACAAGAACTGAGGGTGCAGCACAGATGAAAAAGAAGCGGAAGCTTGGAGTTGGATCAGCAAAGGCATCTCAAAGTAGAAACAAGTTTGGTTTTGAGGATTTGACGTTGCGCTTGAGAAAGAACTTGGCTATGCATCAAGTTTTCCCTCAGGACGAGAAGGAGGCTGCGATCCTTCTCATGGCTTTATCTTATGGCCTTGTTCATTGA

>GmGATA59

ATGGGCAAGCAAGGGCCTTGCTATCACTGTGGAGTTACAAGCACACCACTTTGGCGTAATGGGCCACCTGAGAAGCCAGTACTATGCAATGCATGTGGATCTCGATGGAGGACAAAGGGAACACTTGCAAATTATACCCCTTTGCACGCCCGGGCAGAAAATATTGATTATGAGGATCAAAAGGTTTCCAGGGTAAAGAGCATATCATTAAATAAGAACACAGAAGTGAAATTGGTCAAACGAAAGCAAAACTATGGTAATGCTGCATCTGGAGGGTTTGTTCCTGATTATAGTCAAGGATACCGAAAAGTTGTGGATGAAGATACAAGCAATAGATCAAGCTCAGGGTCAGCTGTCTCTAACTCAGAGAGCTGTGCTCAATTTGGTGGCCCAGATGCTAGTGATTTGACAGGTCCTGCTCAGTCAGTGGTCTGGGATGCCATGGTGCCTTCAAAAAAGAGGACATGTGCAGGTCGTCCAAAGCCTTCATCTGTTGAGAAGCTCACGAGAGACCTGTGCACTATTCTTCATGAACAGCAGTCTTATTTTTCTGCATCTTCTGAAGAGGATCTTCTTTTCGAAAGTGACACACCAATGGTTTCTGTTGAGATAGGACATGGAAGCATTCTCATCAGGCATCCTAGCTCTATAGCTCGTGATGAAGAGTCTGAGGCTAGCTCTCTCTCAGTTGATAATAAACAATGCCTAATGAATGAAGCATATTCATTTTCTAGTACCATTCCTATATATAGTGATCGCAGTAGCATGAACTTCTCATCTCACGGAGTTGAAAAGATCAAAAACTCAGCTGGCCAAATCATGCAACAAGAGAAGCTTGAAAGGGACAAGTCTCAGCTTGAAAAACTACAAGTTCATGGAAATCATGATTCACCATTGTGCTCAATAGATTTAAATGATGTAGTCAACTACGAGGAGTTTATGAGAAACTTGACAAATGAACAGCAGCAGCAATTACTGAAGTATCTCCCAGTGGTTGATACTGCTAAATTTCCTGATAGCCTTAGAAACATGTTCAATAGCTTCCAATTCAAGGAGAACTTAATCTATTTTCAGCAACTTCTCGGGGAAGGAGTCTTTAACATCTCTTTGTTGGGGGCAAAACCTGAAGAATGGAAGACATTAGAAAGGCTTGCATTATCTAATCTGTCAAAGTCGAAATGGGTAGAACACTATAATTTTCTTAAGAAATGTGAAAACAAATCTGGAAAATCTATTGGTTTGGGTTCTACTGCTATGGAATCAAGTAATGTTACAACTGGCAAAAGAATGCGTGAGCATGACAGCCGAAATCAAAATATTCCAGAATTGAAGACAACGATGAGGAGCCCCAAAAGGGTGATCATAAAGCCTCCTAGCTGTGAGGTCAAAGAAGTTGTAGAAGAAGGCTCTAGCTTCAGTCCAAAAAGCTTATTTGCTTTACCCCATGGTGTTGGTGGCTTGCACATGCTGGATTCTTTCAACTTTGTTGGTGAGAGTTCTGAGGATCTGCTTTTAGAGGTGCCTTCTAACAGTTCTTTTCCACAGGCTGAGCTCCTGCACCCATCTTTAAGCTATGGTGCTCGTCAGGTCAGCACCACTAGTAGCTCAGTACACTCACCTGTTACTCATCCTTAA

>GmGATA60

ATGGAATCACCCAGCTCTTCCCCAATTTTCCCACAATTCACCTTCGACAACAACAACTCCGACCACTTCATCGTGGAGGACCTCTTGGACTTCTCCAACGATGACGTTGTCATCACCGATGCCACCTTTGACTCCATCACTACTGACTCTTCCACCGTCACCACCACCGTCCACAGCTGCAACTCCTCCTCCTTCTCTGGCTCCGACCCCAATACCGTCCCTGATATTGGTAGCCGGAATCTCTCCGATGGCCATTTTTCTGATGACCTCTGCGTTCCGTATGATGACATAGCGGAGTTAGAATGGCTTTCGAATTTCGTGGAGGAGTCGTTTTCGAGCGAGGACTTGCATAAGATGCAGCTGATATCAGGCATGAATGCGCAAAACAACGACGTATCAGAGGCCCGCGAGTTCCACTACGAGCCCACCACCACTAGAAGCGGGTCCCACACCCCAGAGCCCACCAGAAACAGCCCAATATTCAATTCGGAAGTGTCGGTTCCAGCCAAGGCCCGCAGCAAGAGGTCCCGTGGGCCCCCTTGCAACTGGGCATCGCGCCTCCTGGTCCTGTCCCCAACGTCGTCGTCCTCGGATAACGAGGTCGTCGTTCCATCTCCCGCCACTGCCGAACCCTGTCCGACTCCGGCGAAAAAAATGGCAAAGGTCGGGCCGAGGAAGAAGGACAGTAGCAGCAGTGACGGAAACGGCAGTGGTGGGGACGGGCGCAGGTGCTTGCACTGCGCCACGGACAAGACCCCGCAGTGGCGGACCGGGCCCATGGGCCCGAAGACTCTCTGCAACGCTTGTGGCGTGAGGTACAAGTCGGGCCGGCTGGTGCCCGAGTACAGGCCCGCGGCAAGCCCAACATTTGTTCTGACTAAGCACTCCAACTCGCACCGCAAGGTGCTGGAGCTGCGAAGGCAGAAGGAAATGGTGCGGTCCCAGCAACACCACCACCAGCATCAACAACAGTTTCTACAACACCACCACCATAACCATCACCACTATCAACATCACCAGAACATGATGTTTGATGTATCCAACGGTGACGATTACTTGATCCACCAATACGTGGGCCCCGATTTCAGGCAGCTTATCTAG

>GmGATA61

ATGACTCCTTATTCTCTGAACCCACCAGGCCCTTCCATACAAGCTGGTCAAACCCAACTCTTCAATATTTCTCCTAATAATCAGGATTGCCGTACCATTTTTAACATATTTGATCCAAGGAAAACCAGGATAGAAATTGGAGGGTTAAGAGATAATTATCATCAACAGGATGACAAGATGATGGTATTGCATGATGGATCATCAAGCAACAGCAACAAGTCGTCCTTCAATAATAATATTTCACCTGAGCCAGTAGTTGTTATGGTTGATCCAATTATAAGCAGCGCATGTGATCAGCAGCATAATTTACCCTATGAGGAAGAGAGTAAAAATATTGATGATCACGGGTCTGGGAACAAATGGATGTCTTCAAAGATGAGGTTAATGAAAAAAATGATGAGACCGAGTATGAGTCCAACCACTGACAAAGCAATCAATTCAGGGCTCGAAAGCAGCAGCAGCAGGTACAGCCAAAGAAGCCTTTGTAACAACAACGCCAGTAGCACCACTAGGGTTTGTTCGGATTGTAATACAAGCACTACCCCACTTTGGAGGAGTGGCCCTAAGGGTCCTAAGTCTCTATGCAATGCCTGTGGCATTAGACAGAGGAAGGCAAGAAGGGCAATGACAAAAGCTACAAGTGGTTTGATCACTCCCATAACATGTGCAAAGACCAGAGTGCACAACAAGGAAAAGAAGTCTCGTGCAAACCATTTTGCACAGTTCAAGAACAAGTACAAGTCCACAACCACAACTAGTGCAGGCTCATCTGAGGGAGTGAGGAAGCTTGAATATTTAAAGGACTTCGCCATAAGCTTGAGGAGTAACAACTCTGATTTTGAACAAGGGTTTCCACGGGATGAAGTAGCTGAAGCGGCATTGCTTCTGATGGACTTGTCTTGTGGTTTTGTCCACTTATAA

>GmGATA62

ATGAATATGGATATGTGCCAAAATGTATCAGTTTCCGGTGAGTGCCAACAAGTGCAGGTTTTTGCCCCTTCTTGCTCAAGCAGCCTTGATGACCTCTTCTCTGCTCAGAACACGGAAGTGGATGTTGAGTTGGAGTGGCTTTCAGAGTTTGTTGAAGACTGTTTTTCAAGCCCTCCAAGCTGTGTCTTGGTACCTATTGGTGTCAAGACTACAAGCACAAGCACCAACCTTTCTTCAGGCACATTGAAGAGACCCCAACAACAAAATGAGTCACCTTTGCAAAACTTTGCTGTGCCAGGGAAGGCAAGGAGCAAAAGGAAGAGGCTTTCAGCCCCAAGAACCAACAAAGACCCTCTAAACATATGGTCACACCATTTGAATCCTCAAAATGAGTCCTTGTGTTCTGACCCTCCTCTTCTCAAACAGGCTTATTGGTTAGCTGACAGTGAGCTCATCATGCCAAAGCCAAAGGATGAGGAGCAAGAAGAGGTGGTGACCAAAGAGGATGAAAAAGTGATTAATGTGATGAGCAAGGAAAGCTTTGGGGACTCTGAGCTTGAGGAGGGTAGCAATGGTCAACAACCAATGCCAACAAGGAGGTGCAGCCATTGCTTGGCTCAGAGGACCCCGCAGTGGAGGGCAGGACCATTAGGTCCAAAGACACTATGCAATGCATGTGGAGTGAGGTACAAGTCTGGTAGGTTGCTACCAGAGTATAGGCCAGCCAAGAGTCCTACTTTTGTTAGCTACTTGCACTCCAATTCCCACAAGAAAGTCATGGAGATGAGGATGGCTGTTTTCTCCACCATTTCTAGTGAGCAGTAG

>GmGATA63

ATGATGGATCTGAAGGAATGGTCATCATCTTCTGAGGAATTGAATGTGAACAGGAAATGTTGCGCTGATTGCAAAACCACCAAGACCCCACTCTGGAGAGGAGGACCAGCTGGACCCAAGACTCTATGCAACGCTTGTGGAATTAGGTATCGGAAGAGAAGGGCATGTTGGAGGAAGGGAGAGTTGAAGAAACAGAAACAGAAACAGAAACAGAGGTGGAAGATGCTGGGGGAAGAGGAACAAGCGGCGGTGTGTTTGATGGCCCTGTCCTGTGGTTTTGTTTTCGCTTGA

>GmGATA64

ATGGTTGGACCAAACTTCATGGACGAGATAGACTGCGGCAGCTTCTTTGACCACATCGACGACCTTCTCGATTTTCCCGTCGAGGACGTTGACGGCGGCGCCGCCACCTTACCGTCGGTAGCCGCTGCCGGCAACTGCAACTCGCTGGCGAGCATCTGGCCCGCTGAGTCCGACTCGTTTCCTACCTCCGACTCGGTGTTTTCCGGCAACACCGCTTCGGACCTCTCGGCGGAGCTCTCGGTTCCGTATGAAGACATTGTCCAATTGGAATGGTTGTCCAATTTTGTGGAGGATTCCTTTTGTGGGGGGAGCCTAACAATGAACAAAGTGGAAGAGCCATCATGTACCACCAAGGAGGACTCAGTAAACACCCAATTTCACACATCAAGCCCAGTTTCTGTCCTTGAAAGTAGCAGTTCTTGCTCTGGTGGCAAGACTTTTCCACTAAGCAGTCCAGAGATTTACATCCCTGTGCCATGTGGACGTACACGCAGCAAGCGTCCACGTCCAGCAACCTTCAATCCTAGGCCTGCCATGAACCTCATTTCCCCTGCCTCTTCTTTTGTTGGGGAGAACATGCAGCCTAATGTCATATCATCCAAGTCATCTTCAGATTCTGAGAATTTTGCTGAGTCTCAACTTGTTCCCAAGATGCCAAAACAAGCTTCTGAGGAGCCTAAGAAGAAAAAGAAAGTGAAGCTGCCACTTCCATTAGTTCCAGCTGATAACAATCAAAATGCCTCACAACCTGTTAGGAAATGCATGCATTGTGAGATAACCAAGACACCACAGTGGAGGGCAGGGCCAATGGGACCAAAAACACTATGCAATGCTTGTGGTGTTCGTTACAAGTCCGGCCGGCTCTTCCCCGAGTACCGGCCTGCTGCAAGTCCAACTTTTTGCCCATCTGTGCACTCCAATTCTCATAAGAAGGTCCTGGAAATGAGATGCAGGGGCATTGACAAATCCGGTTTTGCAATCAATTCAGCTGCCTCACCCGAACTCATTCCAAACACTAACAGCAGCCTGCCCCTGGAGTACATGTGA

The amino acid sequences:

>GmGATA1

MVIANYGFLEHPLCVPQDSLECLGMMNWEGMDSIDSMFSTPWESEKERLEQPEKDKTERKSFTDSGRDAKIWEKRCGHKDARIWERRCSHCDAIKTPQWRTGPFGRNTLCNACGIRFKAGKLYPEYRPADSPTFDVSKHSNVHKEIMKMRNHLS

>GmGATA2

MLYQTPYPQPFQFHHPLPSSFSPLLAVPTTPPPLYLPFPQAEKEMECVEAALKSNYRKEMTLKLSPQTFTEEVSVQNGTTCDDFFVNDLLDFSHVEEEPEQQEDTPCVSLQHENPSHEPCTFKDDYASVPTSELSVLADDLADLEWLSHFVEDSFSEFSAAFPTVTENPTACLKEAEPEPEIPVFSFKTPVQTKARSKRTRNGLRVWPFGSPSFTDSSSSSTTSSSSSSSPSSPLLIYTQSLDHLCSEPNTKKMKKKPSSDTLAPRRCSHCGVQKTPQWRTGPLGPKTLCNACGVRFKSGRLLPEYRPACSPTFSSELHSNHHRKVLEMRQKKETVSVDETGFAPAHVVPSF

>GmGATA3

MGKQGPCYHCGVTSTPLWRNGPPEKPVLCNACGSRWRTKGTLAKYTPLHARAETDDYDDQRVSRVKSISINKKKEVALLKRKQNHDNVVSGGFAPDYNQGYQKVVDEDISNRSSSGSAISNSESCAQFGYGGMDASDLTGPAQSVVWDAMVPSRKRTCVGRPKPSSVEKLTKDLCTILHEQQSYFSVSSEEDLLFESDTPMVSVEIGHGSILIRHPSYIAREEESEASSLSVDNKQCPMSEAYSFSGAIAMHNDSSRLKSSSLEVEKIGNSTGQGMQQEQLKSDKSQLERVQILGNHESPLCSIDLNDVVNYEEFLRILTNEEQQQLLKLLPVVDTAKLPDSLEVMFSSSQFKENLTYFQQLLAEGVFDISLLGAKSEDCKILKRLALSNLSKSKWVAHHNFLKKCKNKAGKSNTMGSTGTTSTNVLNNRASTDVANIKRMRDSRNQNLPEIKTIMRSPKRTIAKASCEGKEAVEDGACYSPKHLFALPPDASFLLLDSLNFVQESSDQDLLLEVSSNTSFPQAELLQPTLSLGAQASTSSSSIYSNLVHH

>GmGATA4

MKERGTFQPLFNALPNSLIILQSFNFIPSLLSTTPSSFPSFLLSQAEKEMECLEAALKSSFRKDMALKQTLFLEEFSSASNVQNVVASSDDLFVDDLLNFSLLENNTNNNNNNEEPDQQLNNHDSTTPQNNQENYNYNPSFNDNNFNTELTVPAEEEVADLEWLSRFVEDSNFSEYSLPFPATVTEKVKVKSPEPGNTAFTFKTPVPAKARSKRTRTGVRVWPLKSPSLAAASSTTTSSSSSSSPSSPQRADSRAKKRAAADGGAARRCSHCGVQKTPQWRTGPLGAKTLCNACGVRYKSGRLLPEYRPACSPTFSSELHSNHHRKVLEMRRKKEDVPEPDTASPPSLPGF

>GmGATA5

MMHHCCGSSQGHVMGTCTCGMYHNHNSSEASSYGSMLFSMPNNNEYYQEHDIYSSFTPSHSSVDCTLSLGTPSTRLTQDEDDNKRHRHQRRSGVTSFCWDLLHSNHNNNNITQSQSKSSSRGSNNNNDSLLARRCANCDTTSTPLWRNGPRGPKSLCNACGIRFKKEQRRASAAGATSASAAVPGGAMESARVYGHHHNNSWYAHSQSQKMMGNELRFMDDSDDRDSDNNGIPFLSWKLNIPDRTSLVDERW

>GmGATA6

MDVCRNVSVSSSECQQELPTLDDLFSHQNTEVDFGLEWLSVFVEDCFSSRPSCLLAPGGVQTTSTSTSTKPSSGTILQRPQQLSHHCPLQNFAVPGKARSKRKRKRLSAPRTTKHTLSTWSQHFSTQNDGVSSDPPLLKQAYWLADSELIVPKKKDVEQEEEEGVVVVVKKEKLGDYCDHDEGDEINNNNSNNDDNVQHPIPRRCTHCLAQRTPQWRAGPLGPKTLCNACGVRFKSGRLLPEYRPAKSPTFVSYLHSNSHKKVMEMRMGVVGVFSTDNNK

>GmGATA7

MIGNFIDDIDCGSFFDHIDDLLEFPDDNAAPVAPPANFWSAESDSLPASYTVFSDNSVTDLSAELSVSYDDIVQLEWLSNFVEDSFSGGSITMKKEEEPQCTTTTKEDIAHAQFQTASPVSVLESSSFCSGEKAASRGPEIYIPVPCGRVRSKRPRPATFNPHPVMQLISPASSTGENVQHNATTTSKAASSDSENFAESVIKGPKQASGEHKNKRKIKVTFSSGQEQQNAPSQAVRKCLHCEITKTPQWRAGPMGPKTLCNACGVRYKSGRLFPEYRPAASPTFCAAVHSNSHKKVIEMRNKTGTKSGFATDSAASPELIPNTNNSLTLEYM

>GmGATA8

MEPSAMYGPSQPLNIPSRIGAGERDDGSGNEPAVDGHHHHIQYETHALDDGAAGGAVVVEDVTSDAVYVSGGGGPEESSQLTLSFRGQVYVFDAVTPDKVQAVLLLLGGCELSSGGSPCVDPGAQQNQRGSMEFPKCSLPQRAASLDRFRQKRKERCFDKKVRYSVRQEVALRMHRNKGQFTSSKKQDGANSYGTDQDSGQDDSQSETSCKHCGTSSKSTPMMRRGPSGPRSLCNACGLFWANRGALRDLSKRNQEHSLPPVEQVDGGNDPDCRTAAADPAQNNLAAFSEPVNPALVADRKVFQSQKMLE

>GmGATA9

MDLYGSFSTPSDCLHIDDFLDFSNITTTTTDTHHHFPPPQNSPSISHDPNFFLNFPSVPSDEAVELEWLSQFVNDEATSFHNIPPPASIGSHTTPFLSNNNRNDNNNEYPKSSSSSPVLAGKSRARREGSVTGDGVRRCSHCATDKTPQWRTGPLGPKTLCNACGVRFKSGRLVPEYRPAASPTFVMTQHSNSHRKVMELRRQKELLRHQQQEQCYRHTHHDFKVC

>GmGATA10

MMDLNVNEKKKCCADCKTTKTPLWRGGPAGPKTLCNACGIRYRKRRACSRKREEQRWKMLGEEEQAAVCLMALSSGFVFA

>GmGATA11

MEVAVAKALKPSLRREFIVQQMLCEDIFSLNANTVAAGEDFSVDDLFDFSNGSLHNEQQQEYDEGKQSLSASEDRGEDDCNSNSTGVSYDSLFSTELAVPAGDLEDLEWVSHFVDDSLPELSLLYPVRSEEANRFVEPEPSAKKTPCFPWEMKITTKARTVRNRKPSNSRMWSLGSPLLSLPSSPSSPSSCSSSVREPPAKKQKKQAQAQPVGAQIQRRCSHCHVQKTPQWRTGPLGAKTLCNACGVRYKSGRLFSEYRPACSPTFCSDIHSNSHRKVLEIRKRKEVAQPDTGLAQAQTQMVPTC

>GmGATA12

MTSVSLNPNPPCPTIQDQSQLFISANNHESTSLSCCTFFHILDQSQTKDIRDLRHGHQQDGKLVFHIGPSNNNNQVCNSSSVKLQPKPVKADSSSECGHHNVSLYKIEDEENKRDHDYEKWMSSTARLTRKMMRLPSTSSDLATKKALNNITRVCADCNTTSTPLWRSGPNGPKSLCNACGIRQRKARRAMAEAVNGFAPSVNSSSTKIRVHHKEKKSRTNHFARFRLKCKLATTSTAEGTSQQENVKIDLNDFGLSLRDSSALKQQVFPIMDEVAQAAMLLMDLSCGFVYC

>GmGATA13

MEAQEFFQNTFCPQFPSDSNITPSNANPSAATTDHFLVEDFFDFSNDDDATFDSLPTDVDSPTVTPVDTTTNSNFPASADAHFPGDLSVPYDDLAELEWLSKFADESFSSEDLQKLQLITGVRAQNDAASSETRDPNPVMFNPQVSVRGKARSKRTRGPPCNWTSRLVVLSPNTKSSSSSHSGAEGGSEGRKCLHCATDKTPQWRTGPMGPKTLCNACGVRYKSGRLVPEYRPAASPTFVLTKHSNSHRKVLELRRQKEMVKVQHQQHQFLQLQHQQNMMFDVPSSNGEDFLIHQHVGPNFTHLI

>GmGATA14

MFGSINQIVSAEDTDGPVSDHHIHYSSHTIEDDGAASDHHIHYSSHTIEEDGAASDHHIHYSSHTIEDDGAAVEDVSAVPGPEISIDNSSQLTLSFRGQVYVFDAVTPDKVQAVLLLLGGNELTSGSQCAELSSRNQTGEEEYPAKCSLPHRAASLNRFRQKRKERCFDKKVRYSVRQEVALRMHRNKGQFTSSKNQDGTNSWGSDQESGQDAVQSETLCCTHCGISSKSTPMMRKGPSGPRSLCNACGLFWANRGTLRDLSKRNLEHSLTPPEQVDEGSNNNALDIRSGIPAQHNNLVNDSKALVSDR

>GmGATA15

MATVNPQPLQFEDPAIPVDDDDDDDDDGGDDDAMDELEDANVNSVNVTNAASVNHEAVVAMPSRTSELTLSFEGEVYVFPAVTPQKVQAVLLLLGGRDVQAGVPAVEPPFDQSNRDMGDTPKRSNLSRRIASLVRFREKRKERCFDKKIRYSVRKEVAQRMHRKNGQFASLKESPGSSNWDSAQSSGQVGTSHSESVRRCHHCGVGENNTPAMRRGPAGPRTLCNACGLMWANKGTLRDLSKGGRNLSVEQSDLDTPIDVKPTSVLEGELPGIHDEQGSSEDPSKSNAADGSSNHAVNPSDEELPETAEHFTNVLPLGIGHSSTNDSEQEPLVELSNPSDTDIDIPGNFD

>GmGATA16

MGKQGPCYHCGVTSTPLWRNGPPEKPVLCNACGSRWRTKGTLANYTPLHARAENVDYEDQKVSRVKSISLNKNKEVKLAKRKQNYDNAASGGFVPDYSQGYQKVVDEDTSNRSSSGSAVSNSESCAQFGGTDASDLTGPAQSVVWDAMVPSKKRTCAGRPKPSSVEKLTRDLCTILHEQQSYFSASSEEDLLFESDTPMVSVEIGHGSILIRHPSSIARDEESEASSLSVDNKQCLMNEAYSFSSTIPMYSDRSGMNFSSHGVEKIKNSAGQIMKQEKLERDKSQLEKLQVPGNHDSPLCSIDLNDVVNYEEFMRNLTNEEQQQLLKYLPVVDTAKFPDSLRNMFNSFQFKENLIYFQQLLGEGVFDISLLGAKPEEWKTLKRLALSNLSKSKWVEHYNFLKKCENKSGKSIGLGSTAMESSYVTTAKRMREHDSQNQNFPELKTTMRSPKRVFIKPSCEVKEVVEEGSSFSPKSLFALPHGVGSLHMLDSFNFVGESSEDLLLEVPSNSSFPQAELLHPSLSYGAQVSTTSSSVHSLVTHP

>GmGATA17

MAKRNGPCFHCGIKSSPHWRSGPEDKSVLCNACGLRYTKWGSIGLQNYFPNHFKPEYLDNLKNLEGRNNVLQGSSYATDSSGKIHVMWNPYVPSRKRSRVVRMTTSIQRFHEQLLMMWKNEENSNDQSSQESEEVLLIDNVNNFIPCNEIGLGCILLKPEDASA

>GmGATA18

MGRKHGPCFHCKIHITPLWRNGPEDKPVLCNACGSRYRKCGSLENYLPNHFQPEYPDNLKMLKRRKTLKGGKGRYLCSPKIPTRKRSPLVRKKITPMKRFYMQLQNMWEDYGNSNESSSEEVLIFNNVNNFIPSNEIGLGCIPLKLDDASA

>GmGATA19

MEVAVAKALKPSLRSEFIVQKMHCEDIFSLNANTVAVGEDFSVDDLFDFSNGSLHNEHQQECDEEKQSLSASSQSQDRGEDDSNSNSTGVSYDSLFSTELAVPAGDLEDLEWVSHFVDDSLPELSLLYPVRSEEANRFVEPEPSVKKTPRFPWEMKITSKARSVRNRKPNTRVWSLGSTLLSLPSSPPAKKQKKRAEAQVQPVGVQIQRRCSHCQVQKTPQWRTGPLGAKTLCNACGVRYKSGRLFSEYRPACSPTFCSDIHSNSHRKVLEIRKRKEVAEPDTGLAQTQMVPTC

>GmGATA20

MEAQEFFQNTFCPQFPSGTNITPSNANPSAATADHFLVEDFFDFSNDDNDATAVTDATFDSLPTDVDSPNVTPLDSTTKNSNLPSSSSADAHFSGDLSVPYDDLAELEWLSKFAEESFSSEDLQKLQLISGVRAQNDAASSETRDPNPVMFNPQVSVRGKARSKRTRGPPCNWTSRLVVLSPNTTSSSSNSDAGKKPATPRRREAAFAEGGSEGRKCLHCATDKTPQWRTGPMGPKTLCNACGVRYKSGRLVPEYRPAASPTFVLTKHSNSHRKVLELRRQKEMVKVQQHQFLQLHQQNMMFDVPSSNGEDYLIHQHVGPDYTHLI

>GmGATA21

MFGSMNKIVSAEDTDGSVSDHHIHYSSHTIEDDGAASDHHIHYSSHTIEEDGAVSNHHIHYSSHTIEEDGGATVEEVSAVPPLEISINDSSQLTISFRGQVYVFDAVTPDKVQAVLLLLGGNELTSGSQCAELSSQNQTGEEEYPAKCSLPQRAASLNRFRQKRKERCFDKKVRYSVRQEVALRMHRNKGQFTSSKNQDGTNSWGSDQESGQDAVQSETLCTHCGISSKSTPMMRRGPSGPRSLCNACGLFWANRGTLRDLSKRNQEHSLAPPEQVDEGSNNNDFDCRSGIPAQHNNLVNDNKA

>GmGATA22

MATVNPQPLQFEDPAIPVDDDDDDDDGGDDDAMDDLEDANVNSVNVAANAAASVNHEAVVAMPSRTSELTLSFEGEVYVFPAITPQKVQAVLLLLGGRDVQARVPAVEQPFDQSNRGMGDTPKRSNLSRRIASLVRFREKRKERCFDKKIRYSVRKEVAQRMHRKNGQFASLKESPGSSNWDSAQSSGQDGTSHSESVRRCHHCGVSENNTPAMRRGPAGPRTLCNACGLMWANKGTLRDLSKGGRNLSVEQSDLDTPIDVKPTSVLEGELPGIHDEQDSSEDPSKSNAADGSSNHAVNPSDEELPETAEHFTNVLPLGIGHSSTNENEQEPLVELSNPSDTDIDIPGNFD

>GmGATA23

MREKFHLCHSFTPQNPTISVSHSKPKQRNPHSFHQNGALRSLSLKPTHTTSLQLLPLKHTIYMEVPEYFVGSFFGTGGAEQFCPPEKRHSDQKTGEPFAIDDLLDFSHADAIMSDGFFDNVAGNSTDSSTVTAVDSCNSSISGSDNRFATTIVPRGFPSDPQFSGELCVPYDEMAELEWLSNFVEDSFSAEEELKTLQLLSGAAAASTAIGAKPQTPESSSSTDTLPPFASDDTLRNAPFLHSETPLPGKARSKRSRAAPGDWSTRLLHLVATEQEKLPQLKAEPAKKREGTNAECSGRKCLHCGTEKTPQWRTGPMGPKTLCNACGVRFKSGRLVPEYRPAASPTFMSTKHSNSHRKVLELRRQKELQRQQHQQLMSQSSIFGVSNGGDEFLIHHHHQHCGPDFRHVI

>GmGATA24

MDLYGSFSTPSDCLHIDDFLDFSNITTDTHHHLPPPQNSPLISHDDANLFFNFPSVPTDEAAELEWLSQFVDDDATSFHSFPATASIGSHSTSFLSNNNNRNDNNEYPKSSLSSNIPCSSAVAGKSRARREGSVTGDGGVRRCSHCASEKTPQWRAGPLGPKTLCNACGVRFKSGRLVPEYRPAASPTFVLTQHSNSHRKVMELRRQKELLRHQQQQQLQQEQCHRHTHNHHDFKVC

>GmGATA25

MDNSLNNPSDNGEDPPPPPPVPMQVDGFQPFHYANCSDEGEEAVPVTNASSAMHARASELTISFEGEVYVFPAVTPEKVQAVLLLLGAQEMTNSAPTSDILLQQNYQDIREINDPSRSSKLSRRFASLVRFREKRKERCFEKKIRYSCRKEVAQRMHRKNGQFASMKEDYKSPAENWDSSNGTPCPESTERRCQHCGISEKSTPAMRRGPAGPRSLCNACGLMWANKGTLRDLSKAARIAFEQNELDTSADIKPSTTEAEHSFAKQDKEGSPEETKPVQMDSSRSPEKTNDQFIIGTAESVTDNLSIQVENHALSLHEQDTLEDLADASGTEFEIPAGFDDQVDIDDSNMRTYWL

>GmGATA26

MVDPTGKGSEIEVEDSNSNPNAPSSGNSPSSNNEQKKTCADCGTTKTPLWRGGPAGPKSLCNACGIRSRKKKRAILGINKGSNEDGRKGKRTGGALGKEVLLHRSHWKKLGEEEKAAVLLMSLSYGSVYA

>GmGATA27

MDNSTLNNPGDNAEDPPPPPAPMQVFDSFQPFHYANGSDEGEEAVPAPVANASSAMRARASELTISFEGEVYVFPAVTPEKVQAVLLLLGAQEMPNSAPTSDFLLQQNYQDIREINDPSRSSKLSRRFASLVRFREKRKERCFEKKIRYSCRKEVAQRMHRKNGQFASLKEDYKSPAENWDSSNGTPCPDSTERRCQHCGISEKSTPAMRRGPAGPRSLCNACGLMWANKGTLRDLSKAGRIAFEQNELDTSADIKPSTTEAKHSYAKQGKEGSPEETKPVQMDSRRSPEKTNEQFIIGTAESVTNNLSVRLENHALILHEQDTLEDLADASGTEFEIPAGFDDQVDIDDANMRTYWL

>GmGATA28

MWYVSQPNHQLLRHVFLHAQPQTTTLHDSDNIYDYSSFTPSSFSSVDCNLSLGTPSTCVSEDEEKRSRHECHSVSNFCWDLLQSKHNNPQSHSKSSGTTNTTDPLLARRCANCDTTSTPLWRNAPVALRYVNNYNI

>GmGATA29

MKKKGPCSHCRISYTPLWPNGPADKPVLCNACGSRYKTRGHLDNYLPKNVHPQPHHKKFKNVNSGGSNLNVEPELESGNQLLNHVSPRSTTNGDSDKLTLDVHHISPQDFGKKIPSKKRSPMVYKRMIPMEKFQKQLVKLYKSERQPEESVLVDNMMNFIPENEIGLGTILLKTNDDDASSTDKCGSSTSAP

>GmGATA30

MEAPEYFVGGYFGAGGAEQFSLSEKRHSDQKTGEPFAIDDLLDFSHADAIMSDGFFDNVTGNSTDSSTVTAVDSCNSSISGSDNHFATAIVPRCYHSDPQFSGELCVPYDEMAELEWLSNFVEDSFSAEEELKTLQLLSGGGAASTAIGAKPQTPESSSSTDTLPPFASRRTLRNAPFLHSETPRPGKARSKRSRAAPGDWSTRLLHLVAPEKEKPPQAKKREGTNVECSGRKCLHCGAEKTPQWRTGPMGPKTLCNACGVRFKSGRLVPEYRPAASPTFMSTKHSNSHRKVLELRRQKEMQRQQHHQQLMSQSSIFGVSNGGDEFSIHHHHHNHHLHCGPDFRHVI

>GmGATA31

MDGIHGGDSRIHITDGQHPIHVPYVQEHEHHGLHHISNGNGIDDDHNDGGDTNCGGSESMEGEVPSNHGNLPDNHAVMMDQGGDSGDQLTLSFQGQVYVFDSVSPEKVQAVLLLLGGREIPPTMPAMPVSPNHNNRGYTGTPQKFSVPQRLASLIRFREKRKERNYDKKIRYTVRKEVALRMQRNKGQFTSSKSNNDESASNATNWGMDENWTADNSGSQQQDIVCRHCGISEKSTPMMRRGPEGPRTLCNACGLMWANKGILRDLSRAAPLSGTIKNENKSLEANQIVHRVAGEADDSS

>GmGATA32

MALVGEQKHSLMVKKGPCSHCGVTHTPLWHDGPTEKPVLCSDCGSQYKLKGNLDNYFPKNPVVQSFHNKFTNVNGGKHLNVDVDQLSNYVPPTDEDNNMSTPNVHCISAQATPLWRNGLVDKSVLHKACELSPQETPASAINAPKKLQLQPLIHKNFINVNGGSSLNVEDEDQLSNHITPASDGDNNKSTPNVQHISPQDFGSKIPSRKRSRVVYLTPLKECMEELWKLHRNYGRHAEERILEDNVNNFIPENEIAGLGAILLKTDHDVAASADTCESSTDD

>GmGATA33

MIPTYRYSVSSPMPIDLNEDHTHHVFSTSHQASSSSSSLSFSILFNPDHQGQGGSCCHWESKHLQSDEEAQKIVPSSESWEHPVSEKDENRSDLKLRVWKKEDKCENFQVEDNSTKWMPLKMRMMRRMMVSDQTGFDTEGMISNSKQIKNEEKNPPLTPLGTDDSNNYNSSANHSKITVRVCSDCHTTKTPLWRSGPKGPKTLCNACGIRQRKARRAIAVAATANGMNPVEAEKSQVKKGNKLHSKGMKSKTKGAPHMKKKRKLGAKYRKRFGAFEDLTVRLSKNLALQKVFPPDEKEAAILLMALSLWPSSWLSHRSLRQLLRFMRYIGGKTIIIV

>GmGATA34

METIGSVDDLLDFSSDIGEEDDYDDKPRKACPSLNSKCAGPSLFNPLVQVDPNHSFSEFAEEELEWLSNKDAFPSVETFVDLSSIQPGTTKNQKSAPVLECSTGSSNSNNSTNSISLLNSCDHLKVPVRARSKSRSRHRPGLAENSSQQVWWRQPSNGTSKADEGMKISSIGRKCQHCGAEKTPQWRAGPSGPKTLCNACGVRFKSGRLVPEYRPASSPTFHSDLHSNSHRKIVEMRRQKQMGMG

>GmGATA35

MVGPNFMDEIDCGSFFDHIDDLLDFPVEDVDGGAATLPSVSAGNSNSLASIWPSESDSFPASDSVFSGNSASDLSAELSVPYEDIVQLEWLSNFVEDSFCGGSLTMNKVEEPSCTTKEDSVNTQFHTSSPVSVLESSSSCSGGKTLPPRSPEIYIPVPCGRARSKRPRPATFNPRPAMNLISPASSFVGENMQPNVISSKASSDSENFAESQLVPKMPKLASGEPKKKKKVKVPLPVAPADNNQNASQPVRKCMHCEITKTPQWRAGPMGPKTLCNACGVRYKSGRLFPEYRPAASPTFCPSVHSNSHKKVLEMRCRGFDKSGFAINSAASPELIPNTNSSLTLEYM

>GmGATA36

MGKQGPCYHCGVTSTPLWRNGPPEKPVLCNACGSRWRTKGTLANYTPLHARAETDDYDDQRVSRIKSISINKKKEVALLKRKQNHDNVMSGGFAPDYNQGYQKVVDEDISNRSSSGSAISNSESCAQFGYGGMDASDLTGPAQSVVWDAMVPSKKRTCVGRSKPSSVEKLTKDLCTILHEQQSYFSASSEEDLLFESDTPMVSVEIGHGSVLIRHPSYIAREEESEASSLSVDNKQCPMSEAYSCSGGILMHNDSSRLKSSSLEVEKIGNSTGQGVLQEQLKSDKSQHERVQILGNHESPLCSIDLNDVVNYEEFLRILTNEEQQQLLKLLPVVDTAKLPDSLKVMFNSSQFKENLTYFQQLLSEGVFDISLLGAKPEDCKTLKILALSNLSKSKWVEHHNFLKKYKNKAVKSNTMGSTGTASINVLNNRASTNVANIKRMCDSRNQNFPELKTIMRSPKRMITKASFECKEAVEDGACYSPKHLFALPPDASSLLLDSFNFVEESGDQDLLLEVPSNTSFPQAELLHPTLSLGAQASTGSSSVYSNLVHH

>GmGATA37

MHRCCSGSQGHVMGPCTCGMFHSQTTSSFAMFFSMPNHKPPPYDDSDNIYDYSSFTPSSSSSVDCTLSLGTPSTRFSEDEEKRSRHERRSVSNFCWDLLQSKHNNPQSHSKSSRTTNTTDPLLARRCANCDTTSTPLWRNGPRGPKSLCNACGIRFKKEERRASAAAATPASAASGGVMESAQVYNNSWYAHQQSQKMQCFSPGMGNEFRFVDDADRDAADNGIPFLSWRLNVTDRTSLVHDFTR

>GmGATA38

MEVAAAKALKPSLRTEFIFPQAIYDEILCFNANNVVADEDFSMDDLLDFSNGEFQVGKDFDDYEEEEDEEKNSTSGSLQSQDRAEDDNNSNSTAGGGGHDYVFAGELSVPADDVADLEWVSHFVDDSLPELSILYPIHCSKKTRVWAEPESRLSPAQTVSKVPRKSRTEKPRKPNTRVWSSFTVFAGSVGFGELVTKKQKKKVEAQSGGAQSLRRCSHCQVQKTPQWRIGPLGPKTLCNACGVRFKSGRLFPEYRPACSPTFCGHIHSNNHRRVLEMRWKKQIAESVTGSDRKQLIPNY

>GmGATA39

MKDCWFFDNNFNGLSDESLDDVMDMELLDLPLDFEDVETDAVEEQDWDAQLKLLEDPPPPLGVFPLQQSSAFCGQTRNENAKLGSKSFSASLAKTVRPAYGKTIPVQKVSLKGKDLLQFQTNSPVSVFESSSSSPSVENSNFELPVIPTKRPRTKRRRLSNISLLYSIPFILTSPAFQKFQRMDFSKSDIQTQPSGELLCKFKKKQRKKDIPLPTNKIEMKRSSSQESVAPRKCLHCEVTKTPQWREGPMGPKTLCNACGVRYRSGRLFAEYRPASSPTFVASLHSNSHKKVLEIRNRATQVTVR

>GmGATA40

MTLITPSSSSSVDCTLSLGTPSTRFSKYEEKRSCHERRSVSNFCWDLLQSKHNNPQSHSKSSQITNTTDPVLVHRCANCDTTYNPLWRNGPHGPKSLCNACGIRFKKEERRASAATAKMQCFSPRMGNEFRFMDDADRVTADNGISFLSWRLNVTD

>GmGATA41

MEVAAAKALKPSLRTEFIFPQAIYDEILCFNANNVVAGEDFSVDDLLDFSNGEFQVGKDFDDYEEDEDEEKGSTSGSLQSQDRTEDDSNSNSTAGGGGDSVFAGELSVPADDVADLEWVSHFVDDSLPELSLLYPVRCSEQTRVCTEPEPRPGSVQTIPAVPRKPRTGKTRKPNARVWSSMSSLCSSVTAKKQKKKVEAQNGGAQSLRRCSHCQVQKTPQWRTGPLGPKTLCNACGVRFKSGRLFPEYRPACSPTFSDDIHSNSHRKVLEMRRKKEIVESDRIQLIPSC

>GmGATA42

MKDCWFFYNNFNGLSDESLDDVMDMEFLDLPLDFEDVETDAVEEQDWDAQFNKFLEDPPPPLGSFPLQSSEFCGQTQHENVKLGKSFRASLPKTVRPTYGKTIPIQNVSLKGKDLLQFQTNSPISVFESSSSSPSVENSNFELPVIPTKRPRNKRQRLSNISLLFSIPFILTSPTFQKCQRMIFSESDLQTQPAGELLCMVSKKLRKKDIPMLANRIEMKRSSSQESVALRKCLHCEVTKTPQWREGPMGPKTLCNACGVRYRSGRLFAEYRPAASPTFVSSLHSDSHKKVLEIRNRATQVTVR

>GmGATA43

MSKDIANMKDSWFFDNNFNGLSDEIFDDVINFFDFPLEDVDANGVEEDWDAQLKCLEDPRFDVYSASSAGLCAETQNEKPQLGMKLSASSNGISPIKQLAKAPGPAYGKTIPHQNVTSNGKDLHQFQTYTYSPVSVFESSSSSSVENSNFDRPVIPVKRARSKRQRPSNFSPLFSIPLIVNLPAVRKDQRTAASDSDFGTNVAGNLSNKVKKQRKKDLSLLSDVEMTRSSSPESGPPRKCMHCEVTKTPQWREGPMGPKTLCNACGVRYRSGRLFPEYRPAASPTFVASLHSNCHKKVVEMRSRVIQEPVRCSMLASSNLHGNSVG

>GmGATA44

MIPAYRHSVSSVMPLDLNEDQNHEFFSPIHHPSSSFSSLSSSYPILFNPPNQDQEARSYDWETTKHLPSHEEEAEKIIPTSGSWGHSVEESEHKVTVWRKEERNENLAEDGSVKWMPSKMRIMRKMLVSNQTDAYTSDNNTTHKFDDHKQQLSSPLGIDDNSSNNYSDKSNNSIVRVCSDCHTTKTPLWRSGPRGPKSLCNACGIRQRKARRAMAAAAAAALGDGAVIVEAEKSVKGKKLQKKKEKKTRIEGAAQMKMKRKLGVGAKASQSRNKFGFEDLTLRLRKNLAMHQVFPQDEKEAAILLMALSYGLVH

>GmGATA45

MSKDIANMKDSWFFDNNFNGLSDEIFDDVINFFDFPLEDVEANGVEEDWDAQLKCLEDPRVDVYTASSAGLCAKTQNEKPQLGMKFSASGNGISPIKQLGKATGPVYGKTITHQNVTSNGKDLHQFQTYTYSPVSVFESSSSSSVENSNFDRPVIPVKRARSKRQRPSSFSPLFSIPFILNSPAMQNHQRIAAADSDFGTNVAGNLSNKLKKQKKKDSSLLSDDVEMMRSSSPESGSPRKCMHCEVTKTPQWREGPVGPKTLCNACGVRYRSGRLFPEYRPAASPTFVASLHSNCHKKVVEMRSRAIQEPVRGSMLASSNLHGNAVG

>GmGATA46

MTPYSLNPPGPSIQAGQNQLFNISPNNQDCRTFFNIFDPRQTSIEIGGLRENYRQDDKMILHDGSSSNCNSSFNISPETVVMVDPLSSACDRRNLPSEEESKNNDHGSGNKWMSSKMRLMKKMMRPSISPTTDKAINSSPRFQNHQGLESRRYSQRSPRNNNGSSTPRVCSDCNTSTTPLWRTGPKGPKSLCNACGIRQRKARRAMAEAANGLVTPIACEKTRLHNKEKKSRMNHFAQFKNKYKSTTTTTTTTVGSSEGVRKLEYFNNFAISLRSNNSDFEQMFPRDEVAEAALLLMDLSCGFVHL

>GmGATA47

MESPNSSPIFPQFTFDTNKNNNNPDNFIVEDLLDFSNDDVVITDATFDSITTDSSTVTTVVDSCNSSSFSGSDPNTVPDVGSQNLSDGHFSGDLCVPYDDIAELEWLSNFVEESFSSEDLQQMQLISGMNARNYDVSEAREFHYEPTTRSGPHTPEPTTKSGGLHYEPTRNSPIFNSEVSVPAKARSKRSRGPPCNWASRLLVLSPTTSSSSDSEVTVPAPAEHGPAPAKKAAKAGPRKKDSGSDGNGSGGDGRRCLHCATDKTPQWRTGPMGPKTLCNACGVRFKSGRLVPEYRPAASPTFVLTKHSNSHRKVLELRRQKEMVRAQQHHQQHHQQQQQFLHHHHHNHNHHHHQHHQNMMFDVSNGDDYLIHQPVGPDFRQLI

>GmGATA48

CFSSPASCVLVPVGVKTTSTKSLSTSINPSLKRPQQQNEPHLQNFVVPGKPRSKRKRLSEPRTNKDPLSIWSHHLNPQIEALCSDPPLLKQAYWLVDSELIMPKPKDNKEQKEEVVIMTKKDEEKVIINRTPQWRVKPLGPKTLCKACGVRYKSGRLLPEYRPSKSPTFVSYLHSNSHKKSWR

>GmGATA49

MEPSAMYGHSQPLSMPSQIGGGESDDGSGNEHAVDGHHHHIQYETHALEDGAAVVVEDVTSDAVYVSGGGGPVESSQLTLSFRGQVYVFDAVTPDKVQAVLLLLGGCELSSGGSPCVDPGAQHNQRGSMEFPKCSLPHRAASLHRFRQKRKERCFDKKVRYSVRQEVALRMHRNKGQFTSSKKQDGANSYGTDQDSGQDDSQSETSCTHCGISSKSTPMMRRGPSGPRSLCNACGLFWANRGALRDLSKRNQEHSLPPVEQVDEGNDSDCRTATADPAHNNLPAFSEHDNPALVADHKVFQSQKMLK

>GmGATA50

MDFGKTNEASNSKLVHDFDLNIAYVEEFDHVNAENEFSSPILVNTTQQACKNSIENMNIEDAIAYNRETTIQANSAAKGATSEDTRQVEPKYIASVSFTPARISQYLRRRRHHRGAESKQSTDPDKLCTNFYCKTRKTPMWRKGPLGPKTLCNACGLQYLKMVKGTGSGLPAAVSDEGDASVPAETVERDSNL

>GmGATA51

MIPTYRYSVSSPMPIDLNEDHTHHLFSTNHQASCSSSSLSYSILFNPDQDQGGSCSDWKSKHLQSDEEAQKIVPSSGLSEKDENKSDLKLRVWKKEDKCENFQGEDNSTKWMPLKMRMMRRLMVSDQTGSDDTEGMISNSQKIKYEEKNSPLSPLGTDDSNYNSSSNHSNITVRVCSDCHTTKTPLWRSGPKGPKSLCNACGIRQRKVRRAIAAAATSNGTNPVEAEKSQVKKGNTLHSKGMKSKTEGAQQMKKNRKLGARYRKRFGAFEDLTVRLSKNFALQQVFPQDEKEAAILLMALSYGLLHGFPTDRYIT

>GmGATA52

MNMDMCQNVSVSGECQQVQVFAPSCSSSLDDLFSAQNTEVDVELEWLSEFVEDCFSSPPSCVLVPVGVKTTSTKSTSTSINPSLKRPQQQNEPPLQNFAVPGKARSKRKRLSAPRTNKDPLSIWSHHLNPQNEALCSDPPLLKQAYWLADSELIMPKPKDKEEQQEEVVIMAKEDEEKVIINVSKEISFGDSELDEGSNGQQQPMPRRCTHCLAQRTPQWRAGPLGPKTLCNACGVRYKSGRLLPEYRPAKSPTFVSYLHSNSHKKVMEMRMSVYSISSEQ

>GmGATA53

MGKVLGNIQFYVMATLFINTELTVPAEEEVADLEWLSHFVEDSNFSEYSLPFPATLAEKVKSPEPGNTGFTYKTSVPTKTRSKPTRTSVRVWPLTSSTVTTTTPTTSSPSSSSPSSPLLAYAAADPRVKKHVVIDSAVAARRCNHCGVQKTPQWRIGPLGAKTLCNACGVRFKSGRLLPEYRPACSPTFSIKLHSNHHRKVLEMRRKKEVTPEPDTSSPRSIPNF

>GmGATA54

MMHHCCGSSQGHVMGTCTCGMYHSETSAYGSMLFSVPNNSEYDMYSSFTPSPSSVDCTLSLGTPSTRLTQDDDHDNDNKRHPHQRRSGVANFCWDLLHSKHNNNNTQSQSKSSSRGSSSNNNNNNDPLLARRCANCDTTSTPLWRNGPRGPKSLCNACGIRFKKEERRASAAAATSTAVPEGEMELARVYGHHHNNSWYAAHSQNQKMMMMGNELRFMDDSEDRDSENNGIPFLSWKLNVPDRTSLVDERW

>GmGATA55

MDMDVCRNISVSSSECQQELPTLDDLFCHQNTEVDFGMEWLSVFVEDCFSSRPSCLLPPSGGGVQTTSTSTKPSSGTIMPRPQQSHHCPLQNFAVPGKARSKRKRLSAPRTTKHTLSTWSQHFSSQNDGVSSDPPLLKQAYWLADSELIVPKKKDVEQEEGVVVVVKKEKLGDYYDDDEGDEVNNNNTNNNNNDNVQHPIPRRCTHCLAQRTPQWRAGPLGPKTLCNACGVRYKSGRLLPEYRPAKSPTFVSYLHSNSHKKVMEMRMGVGVAVLSTDKK

>GmGATA56

MIGNFIDDIDCGNFFDHIDDLLEFPDDAAAADTSAAAPVPPPANFWSAESDSLPATDTVFSDNSVTDLSAELSVPYEDIMQLEWLSNFVEDSFSGGSMTMKKEEPQCTTTKEDIAPAQFQTASPVSVLESSSFCSGEKAGTEINISVPCGRARSKRPRPATFNPNPVMQLISPASSTGENTQHNAANTSKASSDSENFAESVIKAPKQASGEHKKKKKIKVTFPSGQERNAPSQAIRKCLHCEITKTPQWRAGPMGPKTLCNACGVRYKSGRLFPEYRPAASPTFCAAMHSNSHKKVLEMRNKTGTKSGFATVSAASPELIPNTNSSLTLEYM

>GmGATA57

MVDPTGKGSEVEVEDSNSNPNAPSSGNSPSSNNEQKKTCADCGTTKTPLWRGGPAGPKSLCNACGIRSRKKKRAILGINKGSTEDGRKGKRTGGGGGIGGIGGGALGREVLLHRSHWKKLGEEEKAAVLLMSLSYGSVYA

>GmGATA58

MIPAYRHSVSSVMPLDLNEDQNHEFFSPTHHPSSSFSSLSSYPILFNPPNQDQEARSYYWEPTKQYLPSHEEETEKIIPSSGSWDHSVAESEHNKATVWKKAEERNENLESVAAEDGSLKWMPAKMRIMRKMLVSDQTDTYTNSDNNTTHKFDDQKQQLSSPLGTDNSSSNNYSNHSNNTVRVCSDCHTTKTPLWRSGPRGPKSLCNACGIRQRKARRAMAAAAASASGNGTVIVEAKKSVKGRNKLQKKKEKKTRTEGAAQMKKKRKLGVGSAKASQSRNKFGFEDLTLRLRKNLAMHQVFPQDEKEAAILLMALSYGLVH

>GmGATA59

MGKQGPCYHCGVTSTPLWRNGPPEKPVLCNACGSRWRTKGTLANYTPLHARAENIDYEDQKVSRVKSISLNKNTEVKLVKRKQNYGNAASGGFVPDYSQGYRKVVDEDTSNRSSSGSAVSNSESCAQFGGPDASDLTGPAQSVVWDAMVPSKKRTCAGRPKPSSVEKLTRDLCTILHEQQSYFSASSEEDLLFESDTPMVSVEIGHGSILIRHPSSIARDEESEASSLSVDNKQCLMNEAYSFSSTIPIYSDRSSMNFSSHGVEKIKNSAGQIMQQEKLERDKSQLEKLQVHGNHDSPLCSIDLNDVVNYEEFMRNLTNEQQQQLLKYLPVVDTAKFPDSLRNMFNSFQFKENLIYFQQLLGEGVFNISLLGAKPEEWKTLERLALSNLSKSKWVEHYNFLKKCENKSGKSIGLGSTAMESSNVTTGKRMREHDSRNQNIPELKTTMRSPKRVIIKPPSCEVKEVVEEGSSFSPKSLFALPHGVGGLHMLDSFNFVGESSEDLLLEVPSNSSFPQAELLHPSLSYGARQVSTTSSSVHSPVTHP

>GmGATA60

MESPSSSPIFPQFTFDNNNSDHFIVEDLLDFSNDDVVITDATFDSITTDSSTVTTTVHSCNSSSFSGSDPNTVPDIGSRNLSDGHFSDDLCVPYDDIAELEWLSNFVEESFSSEDLHKMQLISGMNAQNNDVSEAREFHYEPTTTRSGSHTPEPTRNSPIFNSEVSVPAKARSKRSRGPPCNWASRLLVLSPTSSSSDNEVVVPSPATAEPCPTPAKKMAKVGPRKKDSSSSDGNGSGGDGRRCLHCATDKTPQWRTGPMGPKTLCNACGVRYKSGRLVPEYRPAASPTFVLTKHSNSHRKVLELRRQKEMVRSQQHHHQHQQQFLQHHHHNHHHYQHHQNMMFDVSNGDDYLIHQYVGPDFRQLI

>GmGATA61

MTPYSLNPPGPSIQAGQTQLFNISPNNQDCRTIFNIFDPRKTRIEIGGLRDNYHQQDDKMMVLHDGSSSNSNKSSFNNNISPEPVVVMVDPIISSACDQQHNLPYEEESKNIDDHGSGNKWMSSKMRLMKKMMRPSMSPTTDKAINSGLESSSSRYSQRSLCNNNASSTTRVCSDCNTSTTPLWRSGPKGPKSLCNACGIRQRKARRAMTKATSGLITPITCAKTRVHNKEKKSRANHFAQFKNKYKSTTTTSAGSSEGVRKLEYLKDFAISLRSNNSDFEQGFPRDEVAEAALLLMDLSCGFVHL

>GmGATA62

MNMDMCQNVSVSGECQQVQVFAPSCSSSLDDLFSAQNTEVDVELEWLSEFVEDCFSSPPSCVLVPIGVKTTSTSTNLSSGTLKRPQQQNESPLQNFAVPGKARSKRKRLSAPRTNKDPLNIWSHHLNPQNESLCSDPPLLKQAYWLADSELIMPKPKDEEQEEVVTKEDEKVINVMSKESFGDSELEEGSNGQQPMPTRRCSHCLAQRTPQWRAGPLGPKTLCNACGVRYKSGRLLPEYRPAKSPTFVSYLHSNSHKKVMEMRMAVFSTISSEQ

>GmGATA63

MMDLKEWSSSSEELNVNRKCCADCKTTKTPLWRGGPAGPKTLCNACGIRYRKRRACWRKGELKKQKQKQKQRWKMLGEEEQAAVCLMALSCGFVFA

>GmGATA64

MVGPNFMDEIDCGSFFDHIDDLLDFPVEDVDGGAATLPSVAAAGNCNSLASIWPAESDSFPTSDSVFSGNTASDLSAELSVPYEDIVQLEWLSNFVEDSFCGGSLTMNKVEEPSCTTKEDSVNTQFHTSSPVSVLESSSSCSGGKTFPLSSPEIYIPVPCGRTRSKRPRPATFNPRPAMNLISPASSFVGENMQPNVISSKSSSDSENFAESQLVPKMPKQASEEPKKKKKVKLPLPLVPADNNQNASQPVRKCMHCEITKTPQWRAGPMGPKTLCNACGVRYKSGRLFPEYRPAASPTFCPSVHSNSHKKVLEMRCRGIDKSGFAINSAASPELIPNTNSSLPLEYM
