# Supplementary material for: Genome-Wide Survey of the Soybean GATA Transcription Factor Gene Family and Expression Analysis under Low Nitrogen Stress
Source: PLoS One. 2015 Apr 17;10(4):e0125174. doi: 10.1371/journal.pone.0125174 (PMC4401516; doi:10.1371/journal.pone.0125174)
Supplement: S2 Text — (DOC) [file pone.0125174.s002.doc]

**S2 Text. Regions of the *INR2*, *NRT1-2* and *NRT2* promoters containing the GATA binding domain.**

*INR2* promoter (-453 ~ -404)

(+) strand

TTAC**GATA**GGATGGCAAACTTATAATGCAACTATTTTTTTTTATTTGTTA

*NRT1-2* promoter (-816 ~ -767)

(+) strand

AATAGTACTATAAAATTAAAACTTATCTTTGAATTTGGTAGCTAGGTAAT

(-) strand

TTATCATGATATTTTAATTTTGA**ATAG**AAACTTAAACCATCGATCCATTA

*NRT2* promoter (-177 ~ -128)

(+) strand

TCCATGGCCCTTGGGAATCCACTTGCCTCCTATCAGACTCTTACGTAGTC

(-) strand

AGGTACCGGGAACCCTTAGGTGAACGGAGG**ATAG**TCTGAGAATGCATCAG
