# Supplementary figures and images for: Genome-Wide Survey of the Soybean GATA Transcription Factor Gene Family and Expression Analysis under Low Nitrogen Stress
Source: PLoS One. 2015 Apr 17;10(4):e0125174. doi: 10.1371/journal.pone.0125174 (PMC4401516; doi:10.1371/journal.pone.0125174)

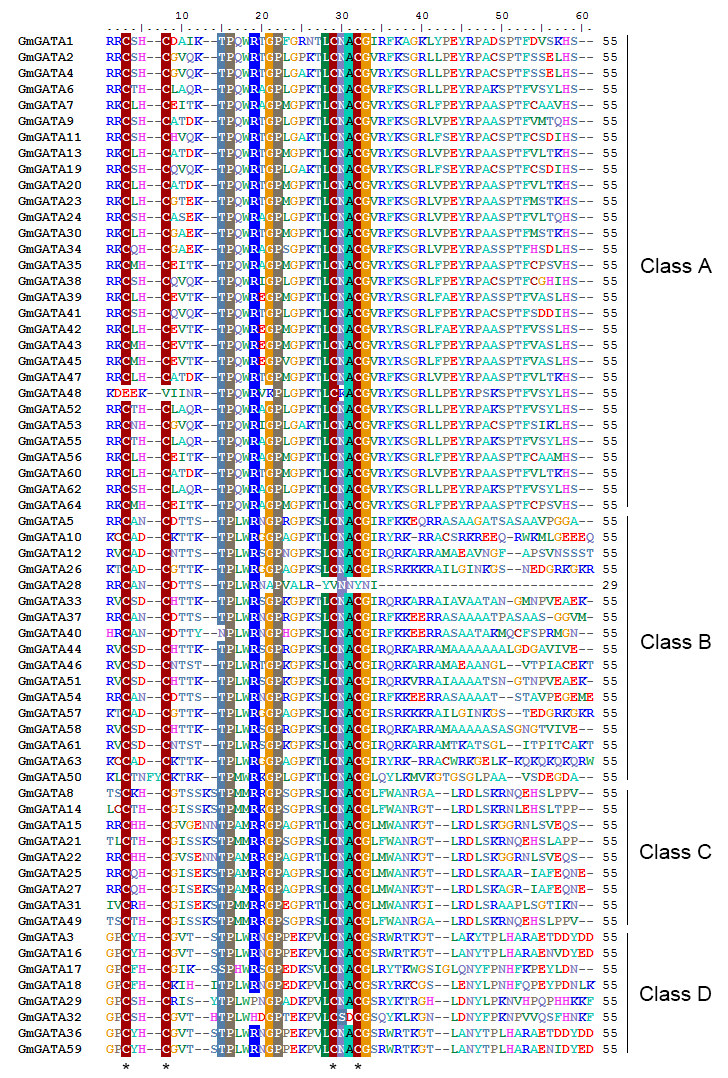

Supplement: S1 Fig — The 55-amino acid regions of 63 soybean GATA domains and the 29-amino acid regions containing the half GATA domain of GmGATA28 were aligned. Residues conserved in all or most of the soybean GATA domains are highlighted. Asterisks indicate the conserved cysteine residues (Cys) in the GATA domain. (TIF) [file pone.0125174.s003.tif]

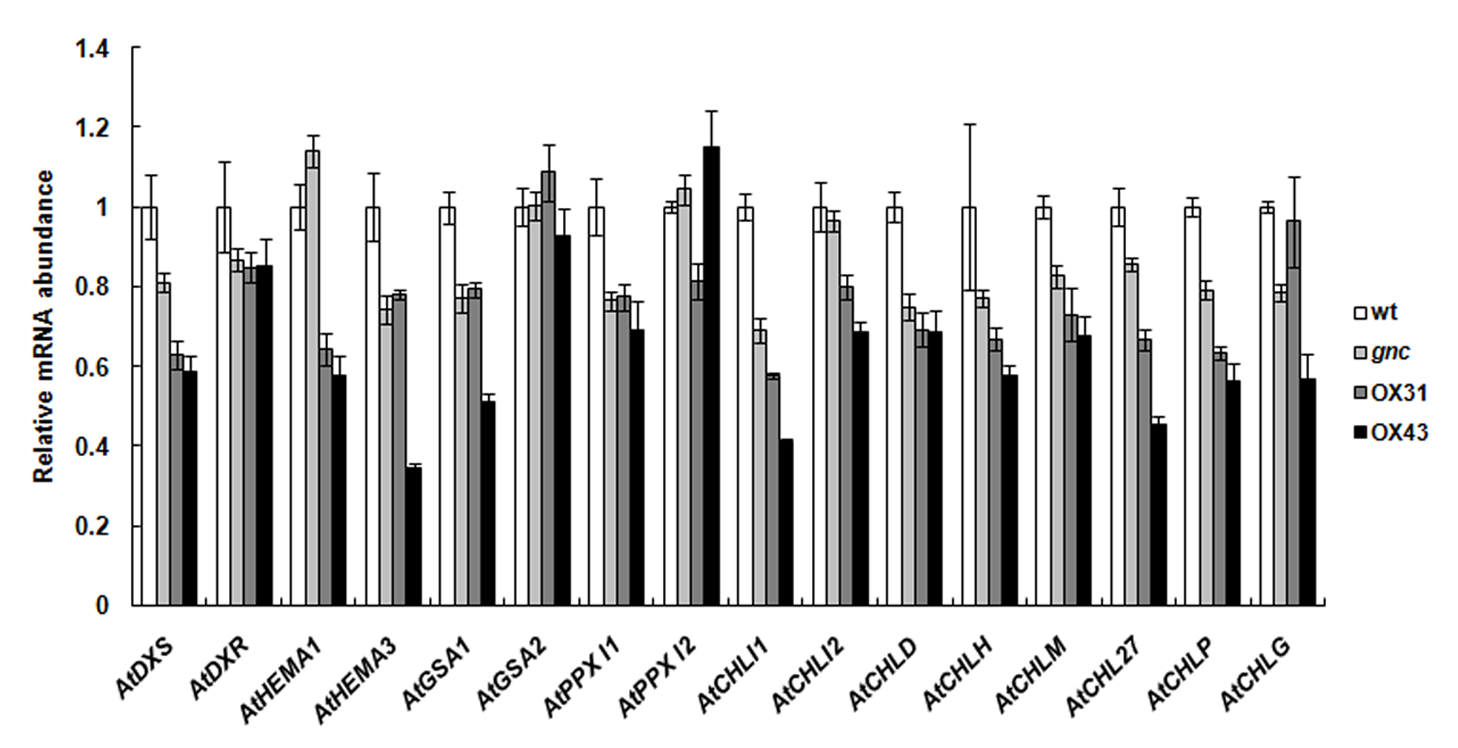

Supplement: S2 Fig — Data were obtained by real-time PCR normalized against the reference gene GAPDH and shown as a percentage of expression in the wild-type plants. (TIF) [file pone.0125174.s004.tif]
