# Supplementary material for: Genome-Wide Survey of the Soybean GATA Transcription Factor Gene Family and Expression Analysis under Low Nitrogen Stress
Source: PLoS One. 2015 Apr 17;10(4):e0125174. doi: 10.1371/journal.pone.0125174 (PMC4401516; doi:10.1371/journal.pone.0125174)
Supplement: S1 Table — (DOC) [file pone.0125174.s005.doc]

**S1 Table. Primers for the real-time PCR of soybean *GATA* genes and the semi-quantitative RT-PCR analysis of *GmGATA44* and *AtGNC*.**

| **Gene** | **Forward primer (5′–3′)** | **Reverse primer (5′–3′)** | **Product (bp)** |
| --- | --- | --- | --- |
| *GmGATA1* | CATTGTGACGCCATAAAAACTCC | CTGGATATAACTTCCCTGCCTTG | 106 |
| *GmGATA2* | GGCTCTTACCCGAATACAG | AAACCAGTCTCATCCACAG | 127 |
| *GmGATA3* | ACATCAAGAGAATGCGTGACAGC | CAGCTTCTTTGCCCTCACAGC | 111 |
| *GmGATA4* | CAAATGGACCTGTTTCAGTGGG | CCTACCACAACCAACGACGG | 84 |
| *GmGATA5* | TGGATGATTCCGATGACAGAGAC | GCTCGTTCGATCTGGAATGTTG | 83 |
| *GmGATA6* | CAGTCCGCAACCATAATTGTGAC | TACGGGAAAGTGCGACTGATG | 88 |
| *GmGATA7* | CTCCAACTCCCACAAGAAGGTTA | CAACTAAGATCCTTCTTCCCTTGC | 163 |
| *GmGATA8* | TTGATGGAGGTAATGACCCAGAC | TTTCTGAGATTGGAAAACCTTACG | 128 |
| *GmGATA9* | CCAGCAGCAAGAACAATG | AAGTAAGGACAGACAAGAGAC | 79 |
| *GmGATA10* | TTGATGGCCTTGTCCTCTGG | TTTACAAGGTTAGGTATGCGACATC | 105 |
| *GmGATA11* | AAATAACGACCAAGGCGAGAAC | GGCAGCGAGAGCAACGG | 88 |
| *GmGATA12* | GGAACATCTCAGCAGGAGAACG | CCATTATTGGAAACACTTGCTGC | 100 |
| *GmGATA13* | TACAAGTCTGGGCGGCTGG | TGATGCTGCTGGTGCTGGAC | 143 |
| *GmGATA14* | GCAAAGTTCTCGTCAATACAAGAGC | AATGCAACTGTACGAGAGTAAAGCA | 143 |
| *GmGATA15* | AGAAGCAGTGATACATACCCGAGAC | GGAACAGCAGTGCTTCTTGACAC | 114 |
| *GmGATA16* | GGAAGACATTAAAAAGGCTTGCG | TGCTCACGCATTCTCTTGGC | 166 |
| *GmGATA17* | GGAAACGTTCACGCGTAGTACG | TCTTCTGATTCTTGGGATGACTGAT | 118 |
| *GmGATA18* | GCAGCCCTAAGATTCCTACGAG | GACTCATTCGAGTTCCCGTAATC | 121 |
| *GmGATA19* | ACGGAATAGGAAGCCCAACAC | CGCCGCTGAATCTGGACTC | 138 |
| *GmGATA20* | TTGTCCTCTCTCCCAACACCAC | TGCGGGGTCTTGTCCGTG | 148 |
| *GmGATA21* | CGAAGAAGACGGCGGCG | ATCGTGAGCTGGCTAGAATCGTT | 87 |
| *GmGATA22* | TGGTTGTCGTCTTCTCTATG | AGGCTATTGGGATATGATGATG | 102 |
| *GmGATA23* | CCGATGGGACCGAAGACG | TGCTGGTGCTGCTGTCGC | 179 |
| *GmGATA24* | TCACACTCACAACCACCACGAT | TGATGTAGATTACCAGCATTTCTCC | 175 |
| *GmGATA25* | CAGGTTGACATTGATGATTCCAAC | ATTTGTTTCCATTGTGAACAGACC | 99 |
| *GmGATA26* | GGAAAAGGATCGGAGATTGAAGTC | CTGAGAAATGGAGGGTGAACTGAC | 183 |
| *GmGATA27* | AGGTTGACATTGATGATGCCAAT | TTTGTTTCCATTGTGAACACAAATC | 99 |
| *GmGATA28* | CTAAAAAACACTTTGGCCCCAT | GTTTGCTTCCTCTTTCTCTCCATC | 116 |
| *GmGATA29* | TGAGCCTGAGCTTGAGTCCG | TTGTCGCTGTCACCATTAGTTGTAG | 78 |
| *GmGATA30* | CCGATGGGACCGAAAACTCTA | AACTTTGACTCATCAACTGCTGATG | 199 |
| *GmGATA31* | GGTGATACTAACTGTGGTGGAAGTG | CACGGAGTCAAAGACATAAACCTG | 162 |
| *GmGATA32* | TGGGAGCAAAATCCCTTCAAG | AAGAATGGCTCCAAGTCCTGC | 184 |
| *GmGATA33* | GAAGCGCAAACTTGGAGCG | AGGGAAGACTTTTTGGAGAGCC | 100 |
| *GmGATA34* | AAGGAAGAAAGGATAAAATGCTGTC | ATGCAGCCACCAATTAGTCAAG | 142 |
| *GmGATA35* | TGTACCACTAAGGAGGACTCGGTC | CTCTGGACTGCGAGGTGGC | 117 |
| *GmGATA36* | GAGGCATATTCTTGTTCTGGTGG | ACTTTTAAGTTGTTCCTGCAGCAC | 129 |
| *GmGATA37* | CAAGACCAACAAGCAAGC | ATCATAACATAACCATCGCAATC | 82 |
| *GmGATA38* | GTCCTCTTTTACGGTGTTTG | CTCCATTGCGGTGTTTTC | 147 |
| *GmGATA39* | CTGGGGAATTATTATGCAAGTTCA | TCTTCATCTCAATTTTGTTAGTCGG | 81 |
| *GmGATA40* | GAGAAGAGCGAGTGCAGCCAC | CGTTATCGGCGGTAACTCTATCAG | 104 |
| *GmGATA41* | GCTGGTTTGGTATTTGTTCC | TCTCCTAATGTCCGTTATCAC | 87 |
| *GmGATA42* | GGAAAAAGGATATTCCCATGCTAG | CCTCACAATGTAAGCATTTCCTGA | 96 |
| *GmGATA43* | AAAGTTGTTGGTTGAGAATGGTATG | ACTCTTTAGCTCCACAGCAATGC | 116 |
| *GmGATA44* | GTCGCTTTGCAATGCCTGC | GCTTCCACAATAACTGCTCCATC | 102 |
| *GmGATA45* | CTTAATTCGCCGGCTATGCA | TTCTGGGGGAACCTGACTCC | 181 |
| *GmGATA46* | AGCCCTCGTAACAACAACGG | ACCATTTGCAGCTTCCGC | 168 |
| *GmGATA47* | CCGACAACCTCATCGTCCTC | CACTGCCGTTGCCATCG | 133 |
| *GmGATA48* | TGCCAAAGCCAAAGGATAACA | ACTCCACATGCTTTGCATAGTGTT | 145 |
| *GmGATA49* | CAGGTTGATGAAGGTAATGACTCG | ATTATCATGCTCTGAGAAAGCAGG | 90 |
| *GmGATA50* | GCCTCTGTCTCCTTTACACCAGC | CTCTGCTTAGATTCTGCACCTCG | 83 |
| *GmGATA51* | GCTGCAACGTCAAACGGAAC | CTATACCTGGCTCCGAGTTTACG | 146 |
| *GmGATA52* | CATGCCAAAGCCAAAGGATAAG | GCACCTTCTTGGCATTGGTTG | 166 |
| *GmGATA53* | ACAACCCCGACAACTTCATCC | GTTTTCTGTACTCCGCAGTGATTG | 149 |
| *GmGATA54* | TGGTACGCCGCACACTCG | CCGTTATTCTCCGAATCCCTG | 98 |
| *GmGATA55* | TTGTGATCCAAATCTAAGTCGTGG | CCCTCGATGGTATATCTGTTTCC | 123 |
| *GmGATA56* | ACCTCCAAGGCATCATCGG | CGCTCTTGACCTGATGGGAAC | 125 |
| *GmGATA57* | GGGAATCAACAAGGGGAGCAC | ATCGGTGCAACAACACTTCCC | 116 |
| *GmGATA58* | CGGAAGATGTTGGTGTCGG | TTGAATAGTTGTTGCTGCTGCTG | 129 |
| *GmGATA59* | TTTGGGTTCTACTGCTATGGAATCA | CCCTTTTGGGGCTCCTCATC | 119 |
| *GmGATA60* | GGACAGTAGCAGCAGTGACGGA | CGTTGCAGAGAGTCTTCGGG | 122 |
| *GmGATA61* | CAAGCAACAGCAACAAGTCGTC | TGAAGACATCCATTTGTTCCCAG | 170 |
| *GmGATA62* | AGGATGGCTGTTTTCTCCACC | TCAATCATAGCATTAAGCACTCTCC | 171 |
| *GmGATA63* | CTTCTGAGGAATTGAATGTGAACAG | TCAACTCTCCCTTCCTCCAACAT | 159 |
| *GmGATA64* | ATGTACCACCAAGGAGGACTCAGTA | GGGATGTAAATCTCTGGACTGCTTA | 129 |
| *ACT11* | ATCTTGACTGAGCGTGGTTATTCC | GCTGGTCCTGGCTGTCTCC | 126 |
| *GmGATA44** | ATGCGGAAGATGTTGGTGTC | ATCGCAGCCTCCTTCTCG | 542 |
| *AtGNC** | GCGTGATTAGGGTTTGTTCG | GCCACCATTGGAGGAGAGT | 268 |
| *UBQ10** | GGCTGATTACAATATCCAGAAGG | ATCCTCCAACTGCTTTCCG | 217 |

Asterisk indicates primers for semi-quantitative RT-PCR anlaysis.
