# Supplementary material for: Genome-Wide Survey of the Soybean GATA Transcription Factor Gene Family and Expression Analysis under Low Nitrogen Stress
Source: PLoS One. 2015 Apr 17;10(4):e0125174. doi: 10.1371/journal.pone.0125174 (PMC4401516; doi:10.1371/journal.pone.0125174)
Supplement: S2 Table — (DOC) [file pone.0125174.s006.doc]

**S2 Table. Primers used for the real-time PCR of some nodulation and nitrogen metabolism-related genes.**

| **Gene** | **Forward primer (5′–3′)** | **Reverse primer (5′–3′)** | **Accession No.** | **Size (bp)** | |
| --- | --- | --- | --- | --- | --- |
| *ENOD40* | GAGAAAGGGGTGTGAGAGGAGAG | CCGCCACTCAAGAAAGAATGTT | X69154 | | 129 |
| *INR1* | GGTGGAAGAATGGTGAAATGGC | CCACCAACCTTCGTCATTAGCA | NM_001251161 | | 135 |
| *INR2* | CTACCGACCAATCTCAAAACTATTACC | CACGGAGTTTATGTTAAGCTCGTTG | NM_001251221 | | 140 |
| *NiR* | AGCCGACGAATACGCCACC | ACCAGTGCATGCCACCAGAG | KF542817 | | 169 |
| *NRT1-2* | GATCTCAATAAGGGGAGATTGGATTA | TGAAGGTCACCACTGCTGCTAGA | NM_001251041 | | 137 |
| *NRT2* | GTTGGCGACGTTTGTGTCG | AGTAGCCCCAGCTCTTCTGATAAG | AF047718 | | 166 |
| *GS1* | GTTGCTGAAGTGCCATGGTACG | AGTGTGCGTCAACAATGTCACG | AF301590 | | 169 |

ENOD: early nodulin gene, INR: inducible nitrate reductase, NiR: nitrite reductase, NRT: nitrate transporter, GS: glutamine synthetase.
