# Supplementary material for: Genome-Wide Survey of the Soybean GATA Transcription Factor Gene Family and Expression Analysis under Low Nitrogen Stress
Source: PLoS One. 2015 Apr 17;10(4):e0125174. doi: 10.1371/journal.pone.0125174 (PMC4401516; doi:10.1371/journal.pone.0125174)
Supplement: S3 Table — (DOC) [file pone.0125174.s007.doc]

**S3 Table. Primers for the real-time PCR of some chlorophyll biosynthesis-related genes.**

| **Gene** | **Forward primer (5′–3′)** | **Reverse primer (5′–3′)** | **Accession No.** | **Size (bp)** |
| --- | --- | --- | --- | --- |
|  | **Methylerythritol phosphate pathway** |  |  |  |
| *DXS* | AACTTACTTTGCGGAGGCATTAG | CATCTTGTTGGGAAGCGACG | At4g15560 | 123 |
| *DXR* | GCCTGATATGCGTTTACCGATTC | AGAACTCCAGTCATTGTGCCTCC | At5g62790 | 195 |
|  | **Tetrapyrrole pathway** |  |  |  |
| *HEMA1* | CACGGGTTTACAATGTGGACG | CCCTCCATGCTTCAAACTGTG | At1g58290 | 126 |
| *HEMA3* | GACAAAAAGACGAGGGAAGCAG | GCTGCTACCAGTCCCATCGTA | At2g31250 | 105 |
| *GSA1* | ATACTTTGGAATCACGCCTGATC | ATCTCCATAATATCACGTCTACCACC | At5g63570 | 99 |
| *GSA2* | GACCGATGTATCAAGCTGGTACG | AAGTATTCATATGTCCCTGGCTGAC | At3g48730 | 103 |
| *PPX І1* | TGAGCATGAAAGCAGCGTTTG | AAGTCCCTTCCTGAAAGAACCAA | At4g01690 | 173 |
| *PPX І2* | CAACTTTTATTGGTGGGAGTAGGAA | TGGTTGACAGACACGGGTTCA | At5g14220 | 127 |
| *CHLI1* | AGACATAGTGACTAACAGAGCAGCAA | AGCAGTTAGGGATAACGGTTGC | At4g18480 | 98 |
| *CHLI2* | GACGCCGAGCTGAGAGTTAAGA | CTTCTTGCAGTTGTAATCTGCTCC | At5g45930 | 125 |
| *CHLD* | CCGATGGTCGAGCCAACATTA | TTGGACGTGGGTCTAGGAGCA | At1g08520 | 82 |
| *CHLH* | ACACCAATCCCAACTCCTTCAG | TCAGCGGAAGTGTCCCAGTAG | At5g13630 | 82 |
| *CHLM* | TGAGATGAAGGCAAAGGCACA | GTCTGCTTTGTTCTGCGGGTA | At4g25080 | 139 |
| *CHL27* | GATTTCGCCGAGTTTGAGCC | AAATCAAGAACCAGCCGTAAACTC | At3g56940 | 80 |
| *PORA* | TGTATTGGAGCTGGAACAAGACC | CCAAAGGTTGAAACACCGAGG | At5g54190 | 162 |
| *PORB* | AAGGCTCGTAAAGTGTGGGAGA | TCGATTGGTACCGAGAGGTGTC | At4g27440 | 99 |
| *PORC* | ACACATACCGCTGTTTCGGC | CAATACACTCCTGACTTCCCAAGA | At1g03630 | 138 |
| *CHLP* | GATGCTGGTGATTACGACTACGC | CACTTAGGGAACACCCAACCATAG | At1g74470 | 146 |
| *CHLG* | CTCTTACGCCAGATGTTGTTGTTC | TGCCAAAAGCTACTGGGAGAGA | At3g51820 | 135 |
| *GAPDH* | CTTGGAAGGAGCTAGGAATTGACA | ATGTGTTTCCCTGCACCTTCTC | At3g26650 | 79 |

DXS: 1-deoxy-D-xylulose-5-phosphate synthase, DXR: 1-deoxy-D-xylulose 5-phosphate reductoisomerase, HEMA: glutamyl-tRNA reductase, GSA: glutamate-1-semialdehyde aminotransferase, PPX: protoporphyrinogen oxidase, CHLI: magnesium chelatase I subunit, CHLD: magnesium chelatase D subunit, CHLH: magnesium chelatase H subunit, CHLM: magnesium-protoporphyrin IX methyltransferase, CHL27: magnesium-protoporphyrin IX monomethyl ester cyclase, POR: protochlorophyllide reductase, CHLP: geranylgeranyl diphosphate reductase, CHLG: chlorophyll synthase, GAPDH: glyceraldehyde-3-phosphate dehydrogenase A subunit.
